# Controversial Roles of the Renin Angiotensin System and Its Modulators During the COVID-19 Pandemic

**DOI:** 10.3389/fphys.2021.624052

**Published:** 2021-02-22

**Authors:** Simon B. Gressens, Georges Leftheriotis, Jean-Claude Dussaule, Martin Flamant, Bernard I. Levy, Emmanuelle Vidal-Petiot

**Affiliations:** ^1^Department of Infectious and Tropical Diseases, Assistance Publique-Hôpitaux de Paris, Bichat-Claude Bernard University Hospital, Paris, France; ^2^Laboratory of Molecular Physiology and Medicine, Université Cote d’Azur, Nice, France; ^3^Sorbonne Université, INSERM, Unité des Maladies Rénales Fréquentes et Rares: des Mécanismes Moléculaires à la Médecine Personnalisée, AP-HP, Hôpital Tenon, Paris, France; ^4^Faculty of Medicine, Sorbonne University, Paris, France; ^5^Department of Physiology, Assistance Publique-Hôpitaux de Paris, Bichat-Claude Bernard University Hospital, Paris, France; ^6^Inserm U1149, Centre for Research on Inflammation, Université de Paris, Paris, France; ^7^Inserm U970, PARCC, Paris, France

**Keywords:** SARS-CoV-2, COVID-19, renin-angiotensin-aldosterone system, RAAS blockers, angiotensin converting enzyme inhibitors, angiotensin receptor blockers

## Abstract

Since December 2019, the coronavirus 2019 (COVID-19) pandemic has rapidly spread and overwhelmed healthcare systems worldwide, urging physicians to understand how to manage this novel infection. Early in the pandemic, more severe forms of COVID-19 have been observed in patients with cardiovascular comorbidities, who are often treated with renin-angiotensin aldosterone system (RAAS)-blockers, such as angiotensin-converting enzyme inhibitors (ACEIs) or angiotensin receptor blockers (ARBs), but whether these are indeed independent risk factors is unknown. The cellular receptor for the severe acute respiratory syndrome coronavirus 2 (SARS-CoV-2) is the membrane-bound angiotensin converting enzyme 2 (ACE2), as for SARS-CoV(-1). Experimental data suggest that expression of ACE2 may be increased by RAAS-blockers, raising concerns that these drugs may facilitate viral cell entry. On the other hand, ACE2 is a key counter-regulator of the RAAS, by degrading angiotensin II into angiotensin (1-7), and may thereby mediate beneficial effects in COVID-19. These considerations have raised concerns about the management of these drugs, and early comments shed vivid controversy among physicians. This review will describe the homeostatic balance between ACE-angiotensin II and ACE2-angiotensin (1-7) and summarize the pathophysiological rationale underlying the debated role of the RAAS and its modulators in the context of the pandemic. In addition, we will review available evidence investigating the impact of RAAS blockers on the course and prognosis of COVID-19 and discuss why retrospective observational studies should be interpreted with caution. These considerations highlight the importance of solid evidence-based data in order to guide physicians in the management of RAAS-interfering drugs in the general population as well as in patients with more or less severe forms of SARS-CoV-2 infection.

## Introduction

Since December 2019, the coronavirus disease 2019 (COVID-19) pandemic has rapidly spread and overwhelmed healthcare systems worldwide. Physicians and scientists urgently attempted to decipher the pathophysiology of the disease in order to define appropriate prevention of viral transmission and management of infected patients. The discovery that the viral receptor was a key enzyme of the renin-angiotensin-aldosterone system (RAAS), namely angiotensin-converting enzyme 2 (ACE2) (Chen Y et al., 2020; Hoffmann et al., 2020), shed light on the potential interactions between the novel coronavirus and the RAAS.

**FIGURE 1:**
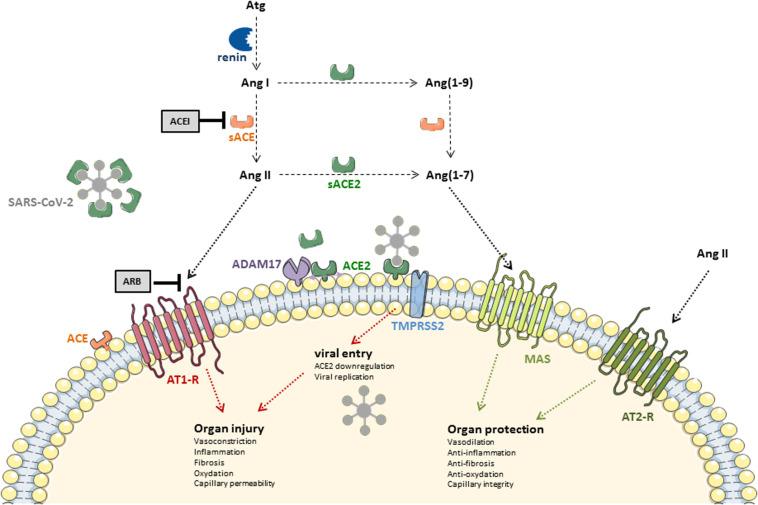
ACE-angiotensin II/ACE2-angiotensin (1-7) homeostasis: general overview. Atg, angiotensinogen; Ang I, angiotensin I; Ang II, angiotensin II; Ang(1-9), angiotensin (1-9); Ang(1-7), angiotensin (1-7); ACEI, ACE inhibitor; ARB, Ang II receptor blocker; ACE, angiotensin converting enzyme; ACE, angiotensin converting enzyme 2; sACE, soluble ACE; sACE2, soluble ACE2; AT1-R, Ang II receptor type 1; AT2-R, Ang II receptor type 2; MAS, G protein-coupled MAS receptor; TMPRSS2, transmembrane protease serine 2; SARS-CoV-2, severe acute respiratory syndrome coronavirus 2; ADAM17, A disintegrin and metalloproteinase 17.

ACE2 is a mono-carboxypeptidase discovered by Donoghue et al. (2000), expressed in the epithelium of the respiratory tract, but also in the intestine, the central nervous system, the heart, the vessels (on endothelial cells), the kidney, and the testicle (Harmer et al., 2002; Hamming et al., 2004; Lee I. T. et al., 2020). In polarized epithelia of the lung, kidney, intestine, ACE2 is found at the apical membrane. In the lung, ACE2 is primarily found on a subset of epithelial cells (type II pneumocytes) facing the airspace, while the angiotensin-converting enzyme (ACE) is expressed on the endothelium, facing the blood. ACE2 had previously been shown to be the receptor for SARS-CoV, but the novel coronavirus has a higher affinity for ACE2 (Wrapp et al., 2020). ACE2 displays significant homology with ACE and is considered as a key counter-regulator of the RAAS, by degrading the octapeptide angiotensin (Ang) II into Ang (1-7) (Chappell, 2016). It also allows the transformation of Ang I into Ang (1-7), via the synthesis of an intermediate metabolite, Ang (1–9), although this pathway is quantitatively marginal. Ang (1-7) binds to the G protein-coupled Mas receptor and induces effects not only on the cardiovascular system, but in fact far beyond, generally “protective” because promoting vasodilation and limiting inflammation, fibrosis, coagulation, and capillary leakage (Kreutz et al., 2020). Biological effects of Ang (1-7) are indeed opposite to those induced by Ang II after binding to its type 1 (AT1) receptor (Figure 1). Importantly, angiotensin converting enzyme inhibitors (ACEIs) do not inhibit ACE2 (Tipnis et al., 2000). In times of emerging infectious threat, the interest toward what appears as a molecular cornerstone in the disease has grown rapidly.

Since early in the pandemic, more severe forms of COVID-19 have been observed in patients with cardiovascular comorbidities such as hypertension, diabetes and coronary heart disease (Wang D. et al., 2020; Wu Y. et al., 2020; Zhou et al., 2020b). Several authors hypothesized that that the link between cardiovascular conditions and severity of the disease might be related to the frequent use of RAAS blockers in these patients. The underlying rationale came from experimental studies in rodents suggesting that RAAS blockers may be responsible for an increased expression of ACE2 (Ferrario et al., 2005a, b; Soler et al., 2009).

While concerns about the safety of these widely used drugs rapidly spread from the medical literature (Aronson and Ferner, 2020; Diaz, 2020; Esler and Esler, 2020; Fang et al., 2020) to general and social media, other authors suggested that modulating ACE-Ang II versus ACE2-Ang (1-7) homeostasis in favor of the latter, using RAAS blockers, might actually be beneficial in patients with COVID-19 (Hanff et al., 2020; Vaduganathan et al., 2020).

Consequently, the management of RAAS-blockers, especially ACEIs and angiotensin receptor blockers (ARBs) in patients at risk of infection or infected with severe acute respiratory syndrome coronavirus 2 (SARS-CoV-2), has been a matter of controversy (South et al., 2020). Many observational studies have attempted to elucidate whether these drugs increased the risk to become infected with COVID-19 and/or modified the course and prognosis of the disease in infected patients while several randomized clinical trials are ongoing to establish whether RAAS blockers should be maintained, discontinued, or even introduced *de novo* in patients with COVID-19.

This review aims to analyze existing data supporting each side of the debate and to describe the available clinical evidence in order to help clinicians understand the underlying pathophysiology and manage prescription of these drugs in the context of the COVID-19 pandemic. The closely related issue of mineralocorticoid receptor antagonists has been less widely debated although it would deserve a dedicated review in itself. The potential role of mineralocorticoid receptor antagonists is beyond the scope of the present review and is only briefly mentioned in this manuscript.

## RAAS Blockers and SARS-CoV-2 Infection: Reasons for Concern

Early concerns regarding the potential deleterious role of RAAS blockers mainly relied on animal data suggesting that ACE2 expression or activity is increased by these drugs, thereby facilitating viral cell entry. However, as reviewed below, this observation is inconsistent in various animal models, is not established in humans, and most importantly, there are no data showing an increased expression of the transmembrane ACE2 protein in the lung. In addition, cardiovascular conditions themselves, independent of RAAS blockade treatment, may also, and probably to a larger extent, influence the expression of ACE2.

### ACE2 Expression and RAAS Blockers – Animal Data

Since the discovery that the ACE2-Ang (1-7)-Mas receptor pathway was a key counter-regulatory system of the ACE-Ang II-AT1 receptor pathway through the past two decades, the role, tissue expression and regulation of ACE2 have been extensively studied and described (Santos et al., 2018), but remain incompletely understood, in part due to discrepant results. In particular, the influence of the pharmacological blockade of ACE and of the AT1 receptor on ACE2 is an unsettled issue.

As reviewed in Supplementary Tables 1A,B, animal studies investigating the effect of ACEIs and/or ARBs on the expression of ACE2 have relied on different animal species, disease models, and readouts (mRNA versus protein level, or enzyme activity). In the early publications warning against the use of RAAS blockers, a widely cited study was that by Ferrario et al. published in 2005. The authors studied the effects of a 12-day treatment of wild-type rats with an ACEI, an ARB, or their combination on expression and activity of the components of the RAAS, in plasma and cardiac tissue. Both losartan (an ARB) and lisinopril (an ACEI) increased the level of ACE2 mRNA in the heart compared to vehicle-treated animals. Losartan, but not lisinopril, also increased ACE2 activity in cardiac membranes [assessed from the rate of conversion of Ang II to Ang (1-7)] and cardiac tissue concentrations of Ang (1-7), while both drugs increased plasma levels of Ang (1-7) (Ferrario et al., 2005a). The same team found somewhat different results in the rat kidney, where lisinopril and losartan did not modify ACE2 gene expression but increased ACE2 enzyme activity in membranes of renal cortex, as well as plasma and urine levels of Ang (1-7) (Ferrario et al., 2005b).

The putative mechanisms underlying an upregulation of ACE2 in the presence of RAAS blockers are partially elucidated. Ang II has been shown to inhibit ACE2 expression through an AT1 receptor-mediated extracellular regulated (ERK)1/2 and p38 mitogen-activated protein (MAP) kinases, both *in vitro* in different cellular models and *in vivo* (Gallagher et al., 2006, 2008a,b; Koka et al., 2008). In addition, Ang II also down-regulates ACE2 at the post-transcriptional level. It has been shown that ACE2 and the AT1 receptor interact on the cell surface and that the binding of Ang II to its AT1 receptor leads the reduction of these AT1 receptor-ACE2 complexes, and to the internalization and ubiquitination of ACE2 (Deshotels et al., 2014). Therefore, the alleged underlying mechanism for an increased expression of ACE2 in the presence of RAAS blockers results from the alleviations of these AT1-receptor mediated inhibiting pathways. According to this hypothesis, both ACEIs (by inhibiting Ang II synthesis) and ARBs (by blocking the AT1-mediated effects of Ang II) are expected to upregulate ACE2, although ARBs interfere more directly with the involved pathway and are thereby potentially expected to have a stronger effect. In line with this, studies seem to have more consistently shown an up-regulation of ACE2 protein concentration in tissues of interest when animals are treated by ARBs (Ishiyama et al., 2004; Igase et al., 2005; Agata et al., 2006; Jessup et al., 2006; Takeda et al., 2007; Soler et al., 2009; Han et al., 2010; Sukumaran et al., 2011, 2012; Zhang et al., 2014), than in studies using ACEIs, which produced more discrepant results (Hamming et al., 2008; Burchill et al., 2012; Burrell et al., 2012; Yang et al., 2013; Zhang et al., 2014; Wang G. et al., 2016), as detailed in Supplementary Tables 1A,B.

However, the biological actions of ACEIS and ARBs on ACE2 in healthy animals remain unclear as not all studies have found similar results, even with each separate class of drug (Supplementary Table 1A). Although many studies have confirmed an increase in ACE2 expression, either at the mRNA and/or protein level, or an increased activity of the enzyme (Ocaranza et al., 2006; Soler et al., 2009; Li et al., 2015), others have shown no effect of RAAS blockers on ACE2 expression or activity (Han et al., 2010; Velkoska et al., 2010; Wösten-van Asperen et al., 2011; Wu C. et al., 2020).

More importantly, although of high relevance in the context of SARS-CoV-2 infection, data on the influence of RAAS blockers on the expression of ACE2 in the lungs are scarce. In ventilated rats with no other cause of lung injury, losartan did not influence ACE2 activity in bronchoalveolar lavage fluid (Wösten-van Asperen et al., 2011), while captopril was found to increase the expression of ACE2 in the lungs of control rats breathing spontaneously (Li et al., 2015). However, variations of ACE2 in lavage fluid may not reflect changes in tissue expression within the lung epithelium. In a recent publication, Wu C. et al. (2020) analyzed the effect of a 14-day infusion of enalapril or losartan on the expression on ACE2 in various tissues (ileum, kidney, heart, and lungs) of wild-type male C57BL/6J mice. Although plasma renin increased by over 100-fold with both drugs, confirming potent RAAS inhibition, neither enalapril nor losartan changed the abundance of ACE2 mRNA in any of the tissues, including the lungs. Likewise, in a recent publication, Wysocki et al. (2020) used kidney and lung lysates to examine the effect of captopril and telmisartan, administered for 2 weeks, on ACE2 expression in mice. Interestingly, in lysates of kidney cortex, ACE2 activity and protein did not differ significantly in captopril- or losartan-treated animals compared with vehicle-treated animals. However, in isolated kidney membranes, there was a profound decrease in kidney ACE2 protein. This decrease in membrane-bound ACE2 was associated with a significant increase in cytosolic ACE2 protein, interpreted as a possible internalization of the protein. In lung tissue, attempts to perform Western blots for ACE2 with either total lysates or isolated membranes did not yield a signal, in agreement with the low physiological expression of the enzyme in type 2 pneumocytes. Conversely, ACE2 activity was low but consistently detected and was not significantly modified by either captopril or telmisartan, both in lung total lysates and lung membranes. Overall, this study showed that ACE2 is not increased by RAAS blockers in these two organs (lung and kidney) that are potential key target sites for SARS-CoV-2 infection.

One potential explanation for these discrepant results is that ACE2 activity does not always correlate with mRNA and protein expression levels. Indeed, several authors have shown uncoupled variations between mRNA, the protein and its activity (Ferrario et al., 2005a, b; Wysocki et al., 2006; Hamming et al., 2008; Yang et al., 2013), revealing potential strong post-transcriptional and post-translational regulations of ACE2. Consequently, based on the readout, conclusions on the impact of RAAS-blockers on ACE2 vary markedly. In addition, as viral entry requires the transmembrane expression of ACE2 in the respiratory tract, any conclusions drawn from mRNA levels, or even from protein level or enzymatic activity of the circulating enzyme is highly speculative. As outlined by Wysocki et al. (2020), when assessing the relative quantities of ACE2 that can act as the SARS-CoV-2 receptor, what matters most is the abundance of full-length, membrane-bound ACE2, which was either not modified or decreased in their well-conducted study with appropriate experimental methodology. Furthermore, specific properties of each molecule within each drug-class may also explain discrepant results which may not be class-related. For instance, captopril is a member of a sulfhydryl class of ACEI that have direct antioxidant properties (Liu et al., 2006).

Other potential explanations for discrepant results between laboratories include the major experimental difficulties associated with the biochemical assessment of the various components of the RAAS, due to the poor specificity of antibodies and of synthetic substrates or inhibitors of ACE2, and to the poor availability of the most accurate assays of ACE2 activity such as high-performance liquid chromatography/mass spectrometry, as reviewed elsewhere (Chappell, 2016; Sparks et al., 2020).

Moreover, the observed effect of RAAS-blockers has been shown to differ between organs, unraveling the complexity of the communications between the local RAAS amongst different tissues. As detailed above, the same team, with similar methods and in the same animal model, found different effects of RAAS blockade in heart and kidney (Ferrario et al., 2005a, b). Effects may also vary within each organ. Burrell et al. (2012) showed that ramipril (an ACE inhibitor) restored ACE2 activity in the renal cortex, but not in the renal medulla after partial nephrectomy. In addition, many experimental studies used very high doses of RAAS blockers (Sriram and Insel, 2020), limiting their physiological relevance. Furthermore, duration (from a few days to several months) and route of administration of treatment are not homogeneous between studies, which may also explain discrepancies. This heterogeneity, combined with the lack of long-term effect evaluation of these drugs in animal models, further limits generalization to human settings. Finally, not only is the membrane bound form of ACE2 required for SARS-CoV-2 binding and infection, but other peptidases are also requisite on the host cells including furin and the transmembrane protease serine 2 (TMPRSS2), which further limits the interpretation of ACE2 expression as a direct explanation for facilitated viral entry (Hoffmann et al., 2020; Wrapp et al., 2020).

Another crucial difference between all experimental models is that although some of them specifically analyzed the impact of RAAS blockade on ACE2 in wild type animals or control conditions (Supplementary Table 1A), others used animal models of pathological conditions such as hypertension, heart failure, or organ injury, in order to analyze their effect in conditions for which they are commonly used in humans (Supplementary Tables 1A,B). However, these disease-models have complex and sometimes strong influences on ACE and ACE2 expressions and activities themselves. Indeed, animal models for diseases such as hypertension (Agata et al., 2006; Takeda et al., 2007; Yang et al., 2013) heart failure (Karram et al., 2005), or lung injury (Asperen et al., 2010; Wösten-van Asperen et al., 2011; Li et al., 2015) were associated with a decreased ACE2 protein expression in most observed tissues compared to wild type or control animals, when other models such as myocardial infarction mostly showed an increased ACE2 expression in the heart (Burrell et al., 2005; Ocaranza et al., 2006; Burchill et al., 2012). In the vast majority of these studies, RAAS blockers tended to restore a physiological level of ACE2 (Supplementary Tables 1A,B).

In summary, animal data on the expression of ACE2 in the presence of RAAS blockers yielded inconsistent results and it is highly uncertain that these treatments actually increase the expression of ACE2 in target organs of SARS-CoV-2. Interpretation of experimental data is all the more complex as (1) the effect differs depending on the animal model, (2) the effects of ACEIs, ARBs, or both combined, may differ, (3) the observed modifications of ACE2 expression vary depending on the tissue examined, (4) the effect on mRNA does not always correlate with the expression on the protein and/or its activity, (5) the level on soluble ACE2, often examined in models, does not reflect the level of transmembrane ACE2 (the only relevant one for viral cell entry), (6) biochemical evaluation of components of the RAAS is technically challenging, (7) other peptidases on the host cells are also requisite for viral entry, and (8) last and most importantly, these are only animals models, and these putative effects and mechanisms need to be confirmed in humans.

Of note, mineralocorticoid receptor antagonists may increase ACE2 expression to a larger extent than ACEIs and ARBs (Keidar et al., 2005; Kreutz et al., 2020).

### ACE2 Expression and RAAS Blockers – Human Data

In human, circulating ACE2 is found at very low levels in healthy subjects (Lew et al., 2008), is higher in men than in women (Soro-Paavonen et al., 2012; Ramchand et al., 2018, 2020), and increases with age (Rice et al., 2006). Plasma ACE2 has been shown to be increased in cardiovascular diseases such as heart failure (Epelman et al., 2009; Uri et al., 2016), coronary artery disease (Ortiz-Pérez et al., 2013; Ramchand et al., 2018), aortic stenosis (Ramchand et al., 2020), and atrial fibrillation (Walters et al., 2016). In addition, the level of plasma ACE2 is a biomarker of poor outcome in these conditions (Epelman et al., 2008, 2009; Ramchand et al., 2018; Ramchand and Burrell, 2020) as well as in the general population (Narula et al., 2020).

Human data on the influence of RAAS-blockers on ACE2 expression, independently of the effect of underlying diseases, is even sparser than animal data and summarized in Supplementary Table 2.

Although transmembrane ACE2 is the form of interest regarding viral entry into target cells, due to obvious difficulties to obtain tissue samples, the influence of RAAS blockers on protein expression of ACE2 in tissues of interest has rarely been studied in humans. In particular, human data on potential modifications of ACE2 expression in the lungs with RAAS-blockers are scarce. Very recently, using a panel of banked human tissue, Lee I. T. et al. (2020) reported that ACE2 robustly localizes within the motile cilia of airway epithelial cells. Interestingly, ACE2 expression was not increased in the nasal airway of ACEI or ARB users, and may even be slightly reduced in patients taking ACEIs. Three studies have described mRNA or protein expression of ACE2 in the kidney (Lely et al., 2004; Reich et al., 2008; Jiang et al., 2020). Neither ACE2 mRNA nor ACE2 protein expression were modified by the use of these drugs among patients with diabetes or membranous glomerulopathies (Lely et al., 2004). However, tubular ACE2 mRNA expression was found to be increased by ACEIs and ARBs in biopsies from patients with chronic allograft nephropathy or primary focal segmental glomerulosclerosis (Reich et al., 2008). In a recent study of more than 400 patients whose kidney transcriptomes were characterized by RNA-sequencing, no association between renal expression of ACE2 and RAAS-blockers was found. Interestingly, age was positively correlated with an increased mRNA expression in both kidney tissues and lung samples from the Genotype-Tissue Expression project (Jiang et al., 2020). In a study exploring the expression of ACE2 in the human intestine, ACEI users (*n* = 9) were shown to have increased ACE2 mRNA levels in duodenum biopsies (Vuille-dit-Bille et al., 2015) compared to non-users (*n* = 22), whereas ARBs were not associated with any modification in the expression of ACE2. Recently, in human myocardial samples, Stegbauer et al. (2020) showed that ACE2 protein level was increased in patients with severe aortic stenosis (but not in patients with severe mitral valve regurgitation) compared to control patients, and that ACEIs, but not ARBs, were associated with increased ACE2 expression (Stegbauer et al., 2020).

In most human studies, ACE2 expression was measured in plasma or urine samples (Supplementary Table 2). To our knowledge, only two studies reported an increased plasma ACE2 activity associated with RAAS blockers; Soro-Paavonen et al. (2012) showed an increase in plasma ACE2 activity among diabetic patients treated with ACEIs (a similar effect with ARBs was only observed in women), while Anguiano et al., reported that ARBs, but not ACEIs, increased plasma ACE2 activity in some subgroups of patients with chronic kidney disease (including those on dialysis). As reviewed in Supplementary Table 2, the reported effects of RAAS-blockers in all other studies were either an unmodified or decreased circulating ACE2. Indeed, several studies have examined the effect of RAAS blockers on plasma ACE2 in patients with heart failure, and consistently shown that the protein concentration and/or activity of the enzyme was either unchanged (Epelman et al., 2008; Uri et al., 2016; Chirinos et al., 2020), or decreased [in one cohort (Sama et al., 2020)], but never increased by these drugs. Similar results were found in chronic (Ramchand et al., 2018) or acute (Ortiz-Pérez et al., 2013) coronary artery disease, as well as in patients with aortic stenosis (Ramchand et al., 2020). A recent large study assessed the potential determinants of ACE2 levels in the general population within a subset of PURE (Prospective Urban Rural Epidemiology) participants. In 5216 subjects with hypertension, the authors found no associations between plasma ACE2 levels and ACEIs or ARBs (Narula et al., 2020).

Therefore, there is a large body of evidence suggesting that RAAS inhibitors do not upregulate plasma ACE2 in human. Of note, methodological considerations are again very important when interpreting studies reporting ACE2 activity, and may explain discrepancies. Indeed, as ACE can cleave the ACE2 fluorescent substrate commonly used to measure ACE2 activity, patients on ACEI may appear to have reduced ACE2 activities compared to controls when an ACE inhibitor is not included in the assay (Chappell, 2016).

Data obtained in urine samples are somewhat discrepant but overall do not support an increased ACE2 expression more than those obtained with plasma samples. Among diabetic patients, Liang et al. (2015) showed a decrease in urine ACE2 concentration with RAAS-blockers, when another study by Mariana et al. (2016) reported no effect of these drugs in patients with preserved renal function. Furuhashi et al. (2015) studied the effect of different anti-hypertensive medications among 100 hypertensive patients compared to 101 healthy controls. An increased urinary ACE2 protein concentration was found with the ARB olmesartan (received by 13 patients), but no significant change was observed with any other ARB (including losartan, telmisartan, valsartan, and candesartan) or with the ACEI enalapril. Of note, a distinct biological effect of olmesartan on ACE2 has not been reported elsewhere to our knowledge.

Very importantly, the level of expression of soluble ACE2 in plasma and urine may not reflect the tissue expression of transmembrane ACE2. Plasma ACE2 originates in part from shedding of the cell surface in tissues in which ACE2 is expressed, mainly in endothelial cells. ACE2 is cleaved from the cell membrane by ADAM17 (A disintegrin and metalloproteinase 17), and the regulation of this process is still poorly elucidated. In a recent study, Ramchand et al. (2020) analyzed plasma and myocardial expression of ACE2 in 22 patients with aortic stenosis and reported that higher circulating ACE2 levels were found in patients with reduced myocardial ACE2 gene expression, suggesting that increased levels of plasma ACE2 might actually reflect downregulation of the enzyme in tissues (Ramchand et al., 2020). Of note, a similar observation of opposite trends for myocardial and plasma activities of ACE2 was reported in dogs with heart failure (Larouche-Lebel et al., 2019). Regarding ACE2 expression in urine samples, ACE2 is unlikely to be physiologically filtered through the glomerulus due its size, so that urine ACE2 probably reflects its tubular expression (mainly in the apical membrane of the proximal tubule), after an ADAM17-mediated cleavage. Altogether, biological mechanisms underlying circulating ACE2 concentration/activity remain mostly hypothetical, as a modified ACE2 plasma concentration could reflect either a dysregulation of shedding or a modified tissue ACE2 expression.

Of note, even if an upregulation of circulating ACE2 was pharmacologically induced, this could actually be expected to be beneficial against SARS-CoV-2 viral infection by the binding of ACE2 to viral particles, preventing them to reach their target cells. This mechanism is the rationale for the therapeutic use of recombinant soluble ACE in the early phase of SARS-CoV-2 infection (Batlle et al., 2020; Monteil et al., 2020).

Overall, data regarding the expression of ACE2 in patients treated with RAAS blockers is scarce, is not in favor of an upregulation, and most importantly, there are no data showing an increased expression of the transmembrane ACE2 protein in the lung or upper respiratory tract (Kreutz et al., 2020), while an increase in the circulating form of the enzyme would not necessarily be deleterious and may even be protective. In addition, the binding of the viral spike protein to transmembrane ACE2 allows attachment of SARS-CoV-2 to the surface of target cells but is not the unique necessary step for viral cell entry. Penetration of the viral particle inside the cell requires the so-called priming of the spike protein by the cellular serine protease TMPRSS2, which then allows fusion of viral and cellular membranes (Hoffmann et al., 2020). In the murine study by Wu et al., mentioned above, the abundance of TMPRSS2 mRNA was not modified in any tissue after enalapril or losartan infusion.

Therefore, there is no solid experimental evidence supporting the concern that RAAS blockers may increase the expression of the transmembrane viral receptor and thereby facilitate its entry into the cell.

### Cardiovascular Comorbidities and COVID-19

As explained above, whereas soluble ACE2 is expressed at very low levels in healthy subjects (Lew et al., 2008), it is markedly increased in cardiovascular disease. Therefore, although the impact of RAAS blockers on the expression of ACE2 remains highly speculative, the underlying conditions for which patients receive RAAS blockers on ACE2 may actually have a much more pronounced impact on ACE2 than these drugs. The reported increased risk of SARS-CoV-2 infection or severe disease in these conditions may indeed be mediated by modulations of ACE2, but these are more likely to be induced by the disease itself. The role of cardiovascular conditions as risk factors for COVID-19 deserves to be discussed and clarified.

The main reason for the initial concern regarding a potential deleterious role of RAAS blockers in COVID-19 arose from the early observation that more severe infections occurred in patients with cardiovascular comorbidities (Wu C. et al., 2020; Zhou et al., 2020b). An early and widely discussed risk factor was hypertension. In the first fairly large case-series published, describing patients in Wuhan (China), hypertension was highly prevalent in hospitalized-COVID-19 cases, and even more frequent among intensive care unit (ICU)-admitted patients (Wang D. et al., 2020). However, it was quickly noted that the vast majority of these results were not adjusted, even for age, whereas the prevalence of hypertension markedly increases with age (Forouzanfar et al., 2017; Wang et al., 2018). Indeed, although nearly all published studies reported an increased crude risk of mortality, ICU admission or severe disease among patients with hypertension, this association did not remain significant after adjusting for the main covariates, especially age and sex in the majority of the studies (Boulle et al., 2020; Bravi et al., 2020; Chen J. et al., 2020; Fried et al., 2020; Gupta et al., 2020; Iaccarino et al., 2020; Ioannou et al., 2020; Kim et al., 2020; Williamson et al., 2020; Yu et al., 2020).

However, a few large and properly adjusted studies still found a significant association between hypertension and mortality (Albitar et al., 2020; Berenguer et al., 2020; Cunningham et al., 2020; Giorgi Rossi et al., 2020; Hernández-Galdamez et al., 2020; Pan et al., 2020; Parra-Bracamonte et al., 2020; Reilev et al., 2020). A potential explanation for these partially discrepant results is that these studies included rather young patients, with a mean or median age below 50 years (Albitar et al., 2020; Cunningham et al., 2020; Hernández-Galdamez et al., 2020; Parra-Bracamonte et al., 2020). A study among very young hospitalized adults (18–34 years old) across the United States (USA) reported an adjusted odds ratio (OR) for death or mechanical ventilation of 2.36 among hypertensive adults (Cunningham et al., 2020). Accordingly, a large study conducted in the United Kingdom based on the National Health Service surveillance system which accounts for approximately 40% of the patients in the country evaluated the factors associated with COVID-19-related deaths compared to the general population (Williamson et al., 2020). The authors showed a strong and significant interaction between hypertension and age, hypertension being associated with a higher risk of mortality up to the age of 70 years, and a lower risk in older patients. Another potential explanation for discrepant results regarding hypertension is that obesity is very important confounder (Lighter et al., 2020; Mechanick et al., 2020; Simonnet et al., 2020) which was not always included in the models, especially as body mass index is a frequent missing data in databases.

Besides hypertension, diabetes mellitus, chronic kidney disease, and ischemic heart disease are other conditions frequently treated with RAAS blockers which were reported to be associated with poor outcome early in the pandemic.

Diabetes mellitus was independently associated with death in several studies. Petrilli et al. (2020) reported an adjusted hazard ratio (HR) of 1.24 (95% confidence interval [95% CI] 1.03; 1.5) for mortality among 5279 COVID-19 patients including 35% with diabetes in the United States. Similar results were reported in other occidental cohorts (Bravi et al., 2020; Giorgi Rossi et al., 2020; Iaccarino et al., 2020; Kim et al., 2020; Reilev et al., 2020; Sands et al., 2020; Williamson et al., 2020) and in the two Mexican case-series (Hernández-Galdamez et al., 2020; Parra-Bracamonte et al., 2020). In cohorts from China, results regarding diabetes have been more variable (Cen et al., 2020; Chen J. et al., 2020; Pan et al., 2020).

Chronic kidney disease was reported to be associated with an increased risk for mortality, independently of potential confounder, in multiple large-scaled studies across different regions worldwide (Berenguer et al., 2020; Boulle et al., 2020; Cariou et al., 2020; Chen J. et al., 2020; Fried et al., 2020; Hernández-Galdamez et al., 2020; Iaccarino et al., 2020; Ioannou et al., 2020; Kim et al., 2020; Mikami et al., 2020; Parra-Bracamonte et al., 2020; Perez-Guzman et al., 2020; Reilev et al., 2020; Williamson et al., 2020). Of note, a few other studies reported that the increased crude risk among chronic kidney disease patients was no-longer significant after adjusting for confounders (Cen et al., 2020; Chen L et al., 2020; Petrilli et al., 2020; van Gerwen et al., 2020). The proportion of patients with chronic kidney disease, and etiology of kidney injury varied markedly between all these studies, which may in part explain these differences. Interestingly, in a French multicenter study of 1,317 diabetic patients (CORONADO study), Cariou et al. (2020) reported adjusted odd ratios (ORs) of 2.14 for mortality [95% confidence interval (CI) 1.16; 3.94] for chronic kidney disease and 2.54 [95% CI 1.44; 4.50] for coronary artery disease.

Overall, in large-scale properly adjusted studies, diabetes mellitus and chronic kidney disease appear to be more frequently associated with mortality, and with higher adjusted risk, than hypertension (Chen J. et al., 2020; Williamson et al., 2020; Yu et al., 2020). Results regarding chronic heart disease are more discrepant (Giorgi Rossi et al., 2020; Ioannou et al., 2020; Kim et al., 2020; Parra-Bracamonte et al., 2020; Reilev et al., 2020), in part because most studies did not differentiate properly the underlying baseline cardiac comorbidities.

Results of studies cited in this paragraph which included more than 500 patients are summarized in Supplementary Table 3 which gives a large, although not exhaustive, overview of studies on the associations between cardio-metabolic comorbidities (in which RAAS blockers are frequently indicated and prescribed) and unadjusted and adjusted risk of adverse outcome in COVID-19.

## The Reverse Hypothesis: RAAS Blockers Might Be Protective in SARS-CoV-2 Infection

While some authors were warning against the potential deleterious role of ACE2 and therefore of RAAS blockers, others have been claiming that restoring a disrupted ACE2-Ang1-7/ACE-Ang II homeostasis, for instance by using RAAS blockers, might actually be beneficial in COVID-19.

### SARS-CoV and SARS-CoV-2 Share a Common Receptor

SARS-CoV-2, a betacoronavirus belonging to the 2B group, shares 70–80% genetic homology with SARS-CoV(-1) (Lu et al., 2020). When ACE2 was identified as the receptor for SARS-CoV in 2003 (3 years after its discovery), both *in vitro* (Li et al., 2003) and *in vivo* (Kuba et al., 2005), this led to investigate the role of ACE2 signaling in respiratory distress syndromes. In the lung, ACE2 is primarily expressed by type II alveolar epithelial cells, endothelial cells, and vascular smooth muscle cells (Wiener et al., 2007) and appears to play a crucial role in the pathophysiology of lung injury from various origins, even when not induced by SARS-CoV (Gu and Korteweg, 2007).

Early in the COVID-19 pandemic, *in vitro* studies have shown that SARS-CoV-2 and SARS-CoV shared the same receptor (Chen Y et al., 2020; Hoffmann et al., 2020). However, the affinity of SARS-CoV-2 for ACE2 was shown to be much higher than that of SARS-CoV (Chen Y et al., 2020; Wrapp et al., 2020). Shortly after, the pathogenicity of SARS-CoV-2 *via* ACE2 was confirmed *in vivo* in a murine model. Intranasally inoculated SARS-CoV-2 allowed viral replication accompanied by interstitial pneumonia and infiltration of inflammatory cells in the lung of transgenic mice bearing the human ACE2 gene, but not in wild-type mice (Bao et al., 2020).

The many clinical and biological resemblances between SARS-CoV (Lew, 2003; Lam et al., 2004) and SARS-CoV-2-related infections, and most importantly, their common receptor, have renewed interest in previous data generated in the years following the SARS outbreak in 2003.

### ACE2-Angiotensin (1-7)/ACE-Angiotensin II Homeostatic Balance and Lung Injury

As summarized in Table 1, the protective role of the ACE2-Ang (1-7)-Mas receptor pathway and, conversely, the deleterious role of the ACE-Ang II-AT1 receptor pathway, have been extensively demonstrated in multiple murine models of acute lung injury.

**TABLE 1:** Summary of the main experimental studies supporting a role of the ACE-Ang II/ACE2-Ang(1-7) homeostasis in lung injury.

Publication	Model/Strain	Intervention	Main findings/conclusions
Imai – 2005 Nature (Imai et al., 2005)	Mouse	Different models of lung injury: acid aspiration, sepsis (caecal ligation perforation)	ACE2 protein expression was downregulated in lung injury. ACE2 (−/−) knockout mice: worsening of lung injury. Recombinant human ACE2 rescued phenotype. ACE (−/−) knockout mice and Losartan (ARB) decreased lung injury → deleterious ACE-Ang II effects mediated through AT1R; ACE2 is protective
Kuba – 2005 Nat Med (Kuba et al., 2005)	Mouse and cell lines	SARS-CoV infection of wild-type or ACE2−/− knock out mice Recombinant SARS-CoV Spike protein ± acid aspiration	ACE2 is a crucial *in vivo* SARS-CoV receptor required for effective viral replication. SARS-CoV infection of wild-type mice resulted in reduced ACE2 expression in the lungs. Spike-Fc binding to ACE2 induced downregulation of ACE2 in cell lines. Treatment with Spike-Fc in mice enhanced acid-induced lung injury, downregulated ACE2 protein expression in lungs, and further (more than acid aspiration) increased Ang II levels. SARS-CoV spike+acid–mediated lung failure was rescued by an ARB.
Wösten-van Asperen – 2010 Am J Pathol (Asperen et al., 2010)	Rat (Sprague-Dawley)	LPS+mechanical ventilation	LPS+MV increased ACE activity, Ang II levels, and inflammation. Captopril (ACEI) attenuated the lung inflammatory response, and the protective effect of Losartan (ARB) was even greater.
Wösten-van Asperen – 2011 J Pathol (Wösten-van Asperen et al., 2011)	Rat (Sprague-Dawley)	LPS+mechanical ventilation	In bronchoalveolar lavage fluid of ventilated LPS-exposed animals, ACE activity was enhanced, and Ang II levels increased, while ACE2 activity was reduced and Ang (1-7) levels decreased. A cyclic form of Ang-(1-7), and to a lesser extent the ARB losartan, restored the Ang (1-7)/Ang II ratio, attenuated the inflammatory response, markedly decreased lung injury, and improved lung function.
Wong – 2012 Am J Resp Cell Mol Biol (Wong et al., 2012)	Rat (Sprague-Dawley) and primary alveolar T1 cells	LPS-induced lung injury	Alveolar type I cells from LPS-instilled rats, as well as primary TI cells treated with LPS, produced cytokines. ACE2 mRNA was decreased in TI cells of LPS-injured animals. In vitro, ACE2 and losartan (ARB) partially inhibited cytokine production → ACE2 modulates pro-inflammatory cytokines production from pneumocytes I through AT1R.
Zou – 2014 Nat Comm (Zou et al., 2014)	Mouse Human (adults)	Avian influenza A H5N1–induced lung injury	H5N1-infected patients exhibited markedly increased serum levels of Ang II. In mice: protein ACE2 expression in lung was decreased and plasma Ang II levels increased among H5N1 infected mice. ACE2(−/−) knock out mice: aggravated lung injury. Recombinant human ACE2 reduced the severity of lung injury.
Li - 2015 Shock (Li et al., 2015)	Rat (Sprague-Dawley)	LPS-induced lung injury	ACEI captopril pretreatment: - significantly attenuated LPS-induced lung injury - inhibited secretion of tumor necrosis factor α and interleukin 6 (IL6) - reduced the Ang II / Ang (1-7) ratio - reversed the increased ACE/ACE2 ratio.
Yan – 2015 Sci China Life Sci (Yan et al., 2015)	Mouse (C57/B6)	Avian influenza A H5N1- induced-lung injury	The ARB losartan: - improved the severity of lung injury and survival rate decreased IL6 mRNA expression - increased ACE2 protein expression in lungs.
Yang – 2015 Sci Rep (Yang et al., 2015)	Mouse (C57BL/6) Adult patients	Avian-origin influenza A (H7N9) -induced lung injury	H7N9 infection down-regulated ACE2 protein expression in lungs and increased plasma Ang II levels, Ang II levels were also increased in 6 patients with H7N9 pneumonia. ACE2 deficiency in mice (ACE2-/- knock out mice) aggravated lung injury and reduced survival. Inhibiting AT1 receptor (with losartan, an ARB) alleviated the severity of lung injury in wild-type and ACE2 knock out mice (no effect with AT2R blocker).
Gu – 2016 Sci Rep (Gu et al., 2016)	Mouse (C57/B6) Human (34 children with RSV pneumonia and 20 healthy children)	Respiratory syncytial virus (RSV)-induced lung injury	RSV-infected children and had increased plasma Ang II levels (particularly in the early phase). Mice infected with RSV had decreased ACE2 protein expression in lung and increased plasma Ang II levels. ACE2 deficiency (knock out versus wild-type mice) aggravated, and ARB (losartan) administration markedly attenuated RSV-induced lung injury. Recombinant hACE2 alleviated the severity of RSV-induced lung injury.
Li – 2016 Sci Rep (Li et al., 2016)	Rat (Sprague-Dawley)	LPS-induced lung injury Lentiviral packaged ACE2 cDNA or ACE2 shRNA was intratracheally administrated two weeks before lung injury	ACE2 overexpression prevented (and ACE2 shRNA deteriorated) LPS-induced lung injury and inflammatory response. ACE2 overexpression reversed Ang II/Ang-(1-7) ratio in the bronchoalveolar fluid lavage. ACE2 suppressed the MAPKs (p38-ERK1/2) and NF-kB pathways that mediate LPS-induced lung injury.
Wang – 2016 Am J Transl Res (Wang L. et al., 2016)	Mouse (BALB/c)	Bleomycin-induced acute lung injury	ACE2 diminished lung injury. ACE2 injection antagonized the effects of PLGF (placental growth factor, member of VEGF family) on increase of lung vessel permeability, resulting in improvement of lung function
Zhang – 2018 Am J Physiol Lung Cell Mol Physiol (Zhang et al., 2018)	Rat (Sprague-Dawley)	Seawater aspiration-induced lung injury	Endoplasmic reticulum stress induced by seawater aspiration led to apoptosis in lung tissue This was inhibited by an ARB and by the addition of Ang (1-7).
Fang – 2019 QJM (Fang et al., 2019)	Mouse (BALB/c)	Hyperoxic (95% O_2_ for 72 hours) lung injury (HLI)	HLI decreased lung ACE2 expression/activity and increased the Ang II/Ang-(1-7) ratio. Lung injury, inflammation and oxidative stress were attenuated by an ACE2 agonist, and aggravated by an ACE2 inhibitor → Activation of ACE2 can reduce the severity of hyperoxic lung injury by inhibiting inflammatory response and oxidative stress. NK-kB and Nrf2 pathways are involved.
Ye – 2020 Exp Mol Pathol (Ye and Liu, 2020)	Mouse (C57BL/6)	Intravenous LPS-induced acute lung injury	LPS administration decreased expression of ACE2, and induced lung injury and inflammation. Lung injury was improved by injection of ACE2. Similar results were found *in vitro*. ACEI and ARB treatments alleviated LPS-induced lung injury.

*ACE, angiotensin converting enzyme; ACE2, angiotensin converting enzyme 2; Ang II, angiotensin II; AT1R, angiotensin II receptor type 1; AT2R, angiotensin II receptor type 2; Ang-(1–7), angiotensin (1-7); ACEI, ACE inhibitor; ARB, angiotensin II receptor blocker; LPS, lipopolysaccharide; ERK, extracellular signal-regulated kinase; NfKB, nuclear factor-kappa B; PLGF, placental growth factor; VEGF, vascular endothelial growth factor; Nrf2, nuclear factor erythroid 2-related factor 2.*

In mouse models of severe lung injury induced by acid aspiration or sepsis, Imai et al., have shown that lung injury was worsened in *ACE2*−/− *knock out* mice compared to wild-type mice, and that recombinant human ACE2 protein partially rescued the phenotype. Conversely, the severity of acute lung injury was attenuated in *ACE*−/− *knock out* mice, in mice lacking the AT1 receptor, as well as in wild-type mice treated with the ARB losartan (Imai et al., 2005).

This crucial role of a disrupted ACE2-Ang (1-7)/ ACE-Ang II homeostasis balance has been confirmed by multiple other studies, in many other murine models of lung injury such as those induced by lipopolysaccharide (LPS) administration (Asperen et al., 2010; Wösten-van Asperen et al., 2011; Li et al., 2015; Ye and Liu, 2020), avian influenza A H5N1 (Yan et al., 2015), H7N9 (Yang et al., 2015), or respiratory syncytial virus (Gu et al., 2016) infections, seawater aspiration (Zhang et al., 2018), bleomycine administration (Wang L. et al., 2016), or hyperoxia (Fang et al., 2019). All these experimental models of acute lung injury have consistently shown a decreased lung expression of ACE2, and/or increased levels of plasma Ang II (Imai et al., 2005; Asperen et al., 2010; Wösten-van Asperen et al., 2011; Zou et al., 2014; Li et al., 2015; Yang et al., 2015; Gu et al., 2016; Fang et al., 2019), as well as a protective and deleterious roles of ACE2-Ang(1-7) and ACE-Ang II, respectively.

Regarding the specific pathogenicity of SARS-CoV, a series of experiments on lung injury induced by SARS-CoV or by the spike protein of the virus (Kuba et al., 2005) confirmed the crucial role of ACE2 as a receptor for the virus by showing that *ACE2*−/− *knock out* mice were markedly protected: viral replication as well as pulmonary lesions were strongly diminished compared with wild type mice. The authors also showed that upon SARS-CoV infection in wild-type mice, ACE2 protein expression in the lung was drastically reduced (Kuba et al., 2006; Gu and Korteweg, 2007). More specifically, binding of the spike protein of SARS-CoV to its receptor downregulated the latter both *in vitro* on cell lines and *in vivo* in the mouse. ACE2 downregulation disrupted the balance between ACE and ACE2 in the lung and increased levels of Ang-II, which then played a key role in lung injury. Downregulation of ACE2 induced by epithelial cell injury during acute respiratory distress syndrome might thereby be amplified by SARS-CoV-2 infection through endocytosis of ACE2 alongside viral particles.

In these models of lung injury, either induced by SARS-CoV or fromother origins, the ARB losartan (Imai et al., 2005; Kuba et al., 2005; Asperen et al., 2010; Wösten-van Asperen et al., 2011; Wong et al., 2012; Yan et al., 2015; Gu et al., 2016; Zhang et al., 2018; Ye and Liu, 2020) and to a lesser extent the ACEIs captopril (Li et al., 2015) or enalapril (Ye and Liu, 2020) have been shown to restore the ACE/ACE2 balance and to attenuate lung lesions and inflammation. Likewise, the administration of recombinant ACE2 (Imai et al., 2005; Zou et al., 2014; Gu et al., 2016; Wang L. et al., 2016), of ACE2 agonists (Fang et al., 2019), or of synthetic Ang (1-7) (Wösten-van Asperen et al., 2011; Zhang et al., 2018) have also been shown to attenuate lung injury. Interestingly, the ability of RAAS blockers to restore the ACE2-Ang (1-7)/ ACE-Ang II balance was not only demonstrated in models of lung injury but also in different models of cardiovascular disease (for instance in a pig model of cardiac arrest), as reviewed in Supplementary Tables 1A,B (Wang G. et al., 2016).

However, as outlined above for studies on pharmacologically induced modifications of ACE2 expression, it is very important while interpreting these studies to carefully consider the limitations of the biochemical assays to quantify the components of the RAAS (Chappell, 2016; Sparks et al., 2020; Chappell et al., 2021).

Importantly, although detailed mechanisms are beyond the scope of this review, the imbalance between the ACE-Ang II and ACE2-Ang (1-7) pathways also likely contributes to the endothelial dysfunction, to the inflammation and cytokine storm, and to the pro-thrombotic state observed in patients with severe forms of the disease. For instance, animal studies of LPS- or viral-induced lung injury have also shown that pharmacological blockade of the ACE-AngII-AT1 receptor pathway, or recombinant ACE2, two ways of restoring the ACE/ACE2 homeostasis, were associated with a decrease of cytokines such as interleukin-6 (IL-6) and tumor necrosis factor alpha (TNF-alpha) (Wong et al., 2012; Yan et al., 2015), and inhibited pivotal inflammatory mediators such as Toll-like receptor 4 (TLR4) (Ye and Liu, 2020), or NF-κB signaling pathways (Li et al., 2016; Fang et al., 2019). Reviews of these other crucial roles of ACE2 deregulation upon SARS-CoV-2 infection can be found elsewhere (Du et al., 2020; Pons et al., 2020; Zhang J. et al., 2020).

Overall, there are solid experimental data supporting a potential protective role of RAAS-blockers in SARS-CoV-2 pneumonia, through a restored ACE2/ACE balance. This is well-illustrated by the very large numbers of studies implemented early in the pandemic to examine whether ARBs such as losartan may be beneficial in patients with COVID-19, as detailed below.

### RAAS and Lung Injury in Human

In line with these solid experimental results, there are some data supporting a similar role for the RAAS, and more specifically protective and deleterious roles for the ACE2-Ang (1-7)-Mas receptor and ACE-Ang II-AT1 receptor pathways, respectively, in human pneumonia and acute respiratory distress syndrome.

Interestingly, a study from China showed increased levels of Ang II in patients with COVID-19 (Liu Y. et al., 2020). In pneumoniae of other origins, such as infections with respiratory syncytial virus in children (Gu et al., 2016), H5N1 (Zou et al., 2014) or H7N9 (Huang et al., 2014; Yang et al., 2015) in adults, small-scaled studies had previously shown increased levels of Ang II in the serum of infected patients, especially in the acute phase of the disease, and that Ang II levels may be associated with disease progression. Of note, in some of these studies (Huang et al., 2014; Zou et al., 2014), the range order of reported Ang II concentrations, including in control subjects (from 1,000 to 10,000 pg/mL, highly variable, and much higher than expected concentrations) questions the methodology used to measure Ang II (Chappell et al., 2021). Importantly, these results obtained in pneumoniae of various origins show that the fact that ACE2 is the viral receptor may amplify disruption of the ACE2/ACE balance, but is not required for this phenomenon.

About half of the variance in plasma ACE activity is explained by the insertion/deletion (I/D) polymorphisms of the enzyme, the D allele being associated with a higher activity. In a population of 96 patients with acute respiratory distress syndrome, the DD genotype frequency was found to be higher than in several control cohorts (Marshall et al., 2002). Similarly, in a cohort of 44 Vietnamese patients with SARS in 2003, the frequency of the D allele was significantly higher in the hypoxemic group than in the non-hypoxemic group (Itoyama et al., 2004).

However, data supporting a protective role of RAAS blockers in human pneumonia are a lot scarcer than in experimental models. In a meta-analysis, Caldeira et al. (2012) have shown that patients treated with ACEIs had a lower incidence of pneumonia (OR 0.66, 95% CI 0.55–0.80 in a total of 19 studies), although a similar effect was not observed for ARBs. In the case of SARS-CoV-2 and COVID-19, numerous observational studies that have examined the link between these treatments and severity of the disease are summarized in the third part of this review.

## Evidence From Clinical Studies

Very early in the pandemic, most scientific societies took position (Bavishi et al., 2020), all in favor of a continued use of both ACEIs and ARBs in patients taking these medication as part of their chronic treatment (Table 2). These recommendations were based on the theoretical considerations and experimental data detailed above, on the clear cardiovascular and renal benefits associated with these medications (Vaduganathan et al., 2020), and on the well-documented risk associated with the discontinuation of RAAS blockers — at least in some indications such as heart failure (Halliday et al., 2019). However, these recommendations were issued before any clinical evidence was available to directly support them, and since then, many studies, mostly observational, have been published to address this issue and are summarized below and in Supplementary Tables 4A,B. However, as recently reviewed (Cohen et al., 2020a), observational studies designed to analyze the effects of RAAS blockers in the COVID-19 pandemic suffer methodological flaws and need to be interpreted with great caution. Many clinical trials have been implemented to provide definite answers regarding the management of these drugs in the context of COVID-19 (Supplementary Table 5).

**TABLE 2:** Recommendations from scientific societies regarding the use of RAAS blockers in the COVID-19 pandemic.

Society	Recommendation
European Society of Hypertension March 12, 2020	Recommend pursuing ACEIs/ARBs due to lack of evidence to support differential use in COVID-19 patients. In those with severe symptoms or sepsis, antihypertensive decision should be made on a case-by-case basis taking into account current guidelines
British and Irish Hypertension Society March 16, 2020	Recommend pursuing ACEI/ARB
Australian Diabetes Society March 29, 2020	Recommend pursuing anti-hypertensive drugs (including ACEI/ARB)
High Blood Pressure Research Council of Australia March 18, 2020	Recommend pursuing ACEI/ARB in the absence of data
European Renal Association – European Dialysis and Transplant Association March 13, 2020	Based on current evidence, recommend not to stop ARB or ACEI
American Heart Association March 17, 2020	Patients taking ACEI and ARB who contract COVID-19 should continue treatment, unless otherwise advised by their physician
Spanish Society of Hypertension March 16, 2020	Recommend that ACEI/ARB should not be empirically stopped in patients who are already taking them; in seriously ill patients, changes should be made on a case-by-base basis
American College of Physicians March 16, 2020	Recommend pursuing ACEI/ARB (no evidence linking them to COVID-19 severity and potential harm of stopping them)
The Renal Association (UK) March 15, 2020	Based on conflicting evidence from basic science studies about the likely effect that modulation of the renin-angiotensin system would have on infection, advise people taking ACEI/ARB to continue to take them
Canadian Cardiovascular Society March 15, 2020	Patients with confirmed or suspected COVID-19 infection should not stop taking an ACEI/ARB/ARNi unless there is a compelling reason to do so (such as symptomatic hypotension or shock, acute kidney injury, or hyperkalemia)
European Society of Cardiology March 13, 2020	Strongly recommend that physicians and patients should continue treatment with their usual anti-hypertensive therapy because there is no clinical or scientific evidence to suggest that treatment with ACEI or ARBs should be discontinued because of the Covid-19 infection
Hypertension Canada March 13, 2020	Strongly encourage continuing ACEI/ARB and Angiotensin Receptor Neprilysin Inhibitors due to a lack of clinical evidence to support withdrawal of these agents
International Society of Hypertension March 16, 2020	There is no good evidence to change the use of ACE-inhibitors or ARBs for the management of raised blood pressure in the context of avoiding or treating COVID-19 infection

### Observational Studies: Association Between Chronic Use of RAAS Blockers and a Positive COVID-19 Test

Several studies have evaluated whether the chronic use of RAAS blockers was associated with an increased risk to contract a SARS-CoV-2 infection (Supplementary Table 4A). Mancia et al. (2020) conducted a population-based case-control study in the Lombardy region of Italy including a total of 6,272 patients with a confirmed SARS-CoV-2 infection matched with 30,759 beneficiaries of Regional Health Service as controls. Even though the use of ACEIs and ARBs was more frequent among patients than controls, this was driven by their higher prevalence of cardiovascular diseases. After adjustment, neither ACEIs (adjusted OR 0.96 and 95% CI 0.87–1.07) nor ARBs [0.95 (0.86–1.05)] were associated with COVID-19 or with a severe or fatal course of the disease. Similar conclusions were reported in two studies conducted in the United States. Mehta et al. (2020) analyzed data from 18,742 patients tested for COVID-19 in Ohio and Florida, of whom 2,285 were taking an ACEI or an ARB, and 1,735 had a positive test. No significant association was found between the use of RAAS blockers and test positivity (overlap propensity score–weighted OR, 0.97; 95%CI, 0.81–1.15) (Mehta et al., 2020). Likewise, in 12,594 patients tested for COVID-19 (of whom 5,894 were positive) in the state of New-York, Reynolds et al. (2020) reported that the likelihood of a positive test was not increased in users of ACEIs/ARBs compared to matched patients, in the total population as well as in the subgroup of hypertensive patients. In a case-control analysis of 571 patients with COVID-19 and 5,710 age- and sex-matched controls, all with hypertension, in Denmark, Fosbøl et al. (2020) reported that ACEI/ARB use was not significantly associated with a higher incidence of COVID-19 compared with other antihypertensive drugs [adjusted HR, 1.05 (95%CI, 0.80–1.36)]. In a case-control study conducted in South Korea using data from the Korean National Health Insurance System, 950 COVID-19 cases among 16,281 subjects with hypertension were retrospectively matched with 1,897 not infected controls, and multivariable-adjusted logistic regression demonstrated the absence of a significant association between exposure to RAAS-blockers and risk of COVID-19 (adjusted OR 1.161 [0.958–1.407)] (Son et al., 2020). Finally, in a large Israeli dataset of 14,520 individuals tested for SARS-CoV-2, of whom 1,317 were found positive, although ACEIs/ARBs were more frequent in positive cases than in negative cases, a multivariable logistic regression model found not significant association between the use of these medications and a positive result (adjusted OR = 1.19; 95% CI 0.96–1.47) (Chodick et al., 2020). A study conducted in Spain had a different design as it aimed to analyze the association between chronic use of RAAS blockers (compared to other antihypertensive drugs) and the risk of COVID-19 requiring hospital admission in 1,139 cases admitted with COVID-19 in seven hospitals in Madrid versus 11,390 matched controls admitted in 2018. Compared with users of other antihypertensive drugs, users of RAAS inhibitors had an adjusted OR for COVID-19 requiring admission to hospital of 0.94 (95% CI 0⋅77–1⋅15). Similar results were found for fatal cases and patients admitted to intensive care units (de Abajo et al., 2020).

Overall, the vast majority of observational studies, in different regions of the world, with different designs and adjustment procedures, consistently concluded that long-term treatment with RAAS-blockers was not associated with an increased adjusted risk of SARS-CoV-2 infection (Supplementary Table 4A).

### Observational Studies: Association Between Chronic Use of RAAS Blockers and Outcome of the Disease in Infected Patients

Multiple observational studies had a different setting as they aimed at evaluating the association between chronic use of RAAS-blockers and outcome of the disease in patients with established COVID-19 infection and are listed in Supplementary Table 4B.

Most studies were conducted in hospitalized patients. Some had norestriction on hypertension and analyzed the association between RAASblockers and outcome in unselected inpatients with COVID-19 (Bean et al., 2020; Holt et al., 2020; Iaccarino et al., 2020; Lahens et al., 2020; Liabeuf et al., 2020; Shah et al., 2020). Except for onestudy which showed an increased risk of severe disease despite – potentially insufficient – adjustment (Liabeuf et al., 2020), and anotherstudy which found a negative association between RAAS blockerexposure and the composite of death or transfer to ICU(Bean et al., 2020), all other studies, and in particular all thosewhich analyzed mortality as an outcome, found no association betweenexposure and outcome after adjustment, although an increased risk was frequent in crude analyses. For instance, in an Italian cohort of 1,581 patients admitted for COVID-19 in 26 hospitals, Iaccarino et al. (2020) reported that neither ACEIs nor ARBS were associated with mortality after adjustment for confounders, although ACEIs were more frequently used in non-survivors. Similarly, in 531 African American patients hospitalized with COVID-19 in Georgia (United States), of whom 207 were on ACEIs/ARBs at baseline, after adjustment for covariates, there was no difference between users and non-users of these drugs in outcomes including mortality (Shah et al., 2020). Other studies restricted analyses to hypertensive patients (Gao et al., 2020; Pan et al., 2020; Tedeschi et al., 2020), or reported results in the subgroup of hypertensive patients (Andrea et al., 2020; Richardson et al., 2020; Trifirò et al., 2020). Similarly, the vast majority of these studies found no association between RAAS blockers and outcome, and in hypertensive patients this was true in unadjusted as well as adjusted analyses. Trifirò et al. (2020) analyzed the charts from 42,926 COVID-19 hospitalized patients by combining multiple Italian databases accounting for approximately a quarter of the Italian population. Almost 50% of the patients had at least one antihypertensive drug claim within 3 months prior to admission. Compared to calcium-channel blockers users, adjusted analyses showed no difference in the risk of death among ACEI (Hazard ratio (HR) 1.01, 95% CI [0.92; 1.12)] or ARB (HR 1.03, 95% CI [0.93; 1.14)] users (Trifirò et al., 2020).

A few studies included both inpatients and outpatients. Again, some had no restriction on hypertension (Fosbøl et al., 2020; Mehta et al., 2020) while others reported results in hypertensive patients (Bravi et al., 2020; Felice et al., 2020; Jung S.Y. et al., 2020; Reynolds et al., 2020; Son et al., 2020) or in patients with an indication for RAAS blockers (Giorgi Rossi et al., 2020). Overall, in most studies, findings were similar to those obtained in hospitalized patients, with no significant association between chronic RAAS blocker exposure and outcome of COVID-19 in adjusted analyses. In a nationwide population-based cohort study of 5,179 confirmed COVID-19 cases in South Korea, Jung et al., found that prior use of RAAS blockers was associated with an increased risk of in-hospital mortality in unadjusted analyses [OR 3.88, 95% CI (2.48; 6.05)], but this difference was ironed out when adjusted for age, sex, Charlson comorbidity index, immunosuppression and hospital type [adjusted OR: 0.88, 95% CI ([0.53; 1.44)]. In the subgroup of 1,157 hypertensive patients, there was no association between RAAS blockers and mortality, both in unadjusted [OR 0.74, 95% CI (0.43; 1.28)] and adjusted [0.71, 95% CI (0.40; 1.26)] analyses (Jung S.Y. et al., 2020). In a Danish retrospective cohort of 4,480 patients with COVID-19, of whom 895 were chronic ACEI/ARB users in a 6-month period prior to diagnosis, the unadjusted HR for mortality was 2.65 [95% CI (2.18; 3.23)] while the adjusted HR, after accounting for age and medical history, was 0.83 [95% CI (0.67; 1.03)] (Fosbøl et al., 2020). Reynolds et al. (2020) reported similar results among a cohort of 5,894 patients with COVID-19, of whom 2,573 with hypertension. After careful adjustment, no significant association was found between chronic-exposure to ACEIs/ARBs and severe illness, defined as a composite of intensive care admission, mechanical ventilation, or death, in all patients as well as in hypertensive patients (Reynolds et al., 2020).

Altogether, if some studies conducted in unselected population identified an association of RAAS blocker prescription with mortality in unadjusted analyses, once adjustment for age, sex and comorbidities was performed, chronic use of RAAS-blockers was not associated with worse outcome (in particular mortality) among patients with COVID-19, hospitalized or not. All these studies support the above-mentioned recommendations from scientific societies, not to discontinue these treatments despite the ongoing pandemic.

One randomized trial (CORONACION, NCT 04330300) had been designed to address this specific question in Ireland. The aim was to randomize patients with hypertension taking ACEIs/ARBs to either continue or switch to an alternative blood pressure medication and analyze COVID-19-related events. However, this trial has been interrupted due to a low incidence of COVID-19 in the study site.

Although potentially highly relevant, the specific role of mineralocorticoid receptor antagonists has been less studied, in part because these drugs are much less frequently prescribed than ACEIs and ARBs. In a recent very large observational study from a Swedish national registry, among 1,387,746 patients with a potential indication for RAAS blockers, 5.8% received a mineralocorticoid receptor antagonist. These medications were not associated with the risks of hospitalization for COVID-19 or mortality after adjustment for confounders (Savarese et al., 2020).

### Observational Studies: Association Between in-Hospital Use of RAAS Blockers and Outcome of the Disease in Infected Hospitalized Patients

Most studies which analyzed “in-hospital” (and not chronic) exposure to treatment showed a strong protective effect associated with the use of RAAS blockers after adjustment for baseline comorbidities (Cannata et al., 2020; Chaudhri et al., 2020; Meng et al., 2020; Yang et al., 2020; Zhang P et al., 2020; Zhou et al., 2020a). For instance, in a retrospective analysis of 1,128 COVID-19 patients with hypertension admitted in 9 hospitals in the epicenter region of the pandemic in Hubei, China, authors compared patients based on their in-hospital anti-hypertensive regimen. They recorded 188 patients receiving ACEI/ARB during hospitalization and all-cause mortality at 28 days was significantly lower among them. This effect remained significant in a mixed-effect Cox model (using site as a random effect, after adjusting for age, gender, comorbidities, and in-hospital medications), with an adjusted hazard ratio of 0.42 [0.19–0.92] and in a propensity score-matched analysis (adjusted HR, 0.37; 95% CI, 0.15–0.89) (Zhang P et al., 2020).

However, as outlined in a letter by Cohen et al. (2020b) to warn the reader against interpretation of the study by Zhang P et al. (2020), as demonstrated in a dedicated study from our team (Lahens et al., 2020), and as recently discussed in a review on the methodology of observational studies focused on the issue of RAAS blockers and COVID-19 (Cohen et al., 2020a), this protective effect is majorly biased. Indeed, our study showed that cessation of a chronic RAAS blocker exposure upon hospital admission is frequent and occurs in those with the worst outcomes, and conversely for treatment continuation (Lahens et al., 2020). Therefore, treatment discontinuation is related directly or indirectly to disease severity and mortality. This induces a phenomenon of reverse causality whereby severity of the disease causes treatment cessation, and not the reverse. Conversely, the continued treatment arm is prone to immortal-time bias (Suissa, 2008). Overall, this generates a “healthy user-sick stopper bias” explaining that studies based on in-hospital exposure (instead of chronic exposure) find a spurious protective association between RAAS blockers and outcome in COVID-19 (Supplementary Table 4B).

Observational pharmaco-epidemiological studies are not suited to analyze the association between in-hospital exposure to RAAS blockers and outcome of COVID-19. The answer to this issue can only be provided by interventional randomized trials.

It is very important to note that most meta-analyses to date meant to analyze the association between RAAS-blockers and outcome have incorporated studies based on in-hospital exposure (Baral et al., 2020; Flacco et al., 2020; Greco et al., 2020; Grover and Oberoi, 2020; Guo X. et al., 2020; Mackey et al., 2020; Pranata et al., 2020; Zhang X. et al., 2020), so that their conclusions should be interpreted with great caution.

### Clinical Trials: Randomized Studies on Discontinuation or Continuation of RAAS Blockers in Previously Treated Patients Hospitalized for COVID-19

As of January 2021, optimal management of ACEIs/ARBs in patients with a COVID-19 infection remains uncertain. Several trials randomizing COVID-19 patients previously treated with RAAS blockers and admitted to hospital for treatment continuation or discontinuation (ACORES-2 in France, NCT04329195; ACEI-COVID in Austria, NCT04353596; RASCOVID-19 in Denmark, NCT04351581; RAASCOVID in Canada, NCT04508985; SWITCH-COVID in Brazil, NCT04493359) are currently recruiting (Supplementary Table 5), while results have been published for two trials.

The BRACE-CORONA study (NCT04364893) enrolled 659 patients hospitalized with a confirmed diagnosis of COVID-19 from 29 sites in Brazil, with chronic use of ACEIs/ARBs and randomly allocated to continuing or stopping these treatments for 30 days (Lopes et al., 2020). Results were presented at the European Society of Cardiology Congress in September 2020 and published very recently (Lopes et al., 2021). No difference was reported in the number of days alive and out of hospital at 30 days (primary outcome) between the suspending ACEIs/ARBs group and the continuing group. There was no difference in all-cause mortality at 30 days either (HR 0.97 (95% CI [0.38; 2.52)].

An international randomized trial (REPLACE COVID, NCT04338009) was published in January 2021: 152 patients hospitalized for COVID-19 and receiving an ACEI or an ARB before admission were randomly assigned to continuation or discontinuation of this treatment. No difference in the primary endpoint assessing severity of disease course (a global rank score across four hierarchical tiers incorporating time to death, duration of mechanical ventilation, time on renal replacement or vasopressor therapy, and multiorgan dysfunction during the hospitalization) was observed between patients who continued or discontinued RAAS blocker therapy (Cohen et al., 2021). The authors concluded that RAAS blockers can be safely continued in patients with COVID-19 requiring hospital admission.

### Clinical Trials: Randomized Studies on the Use of ARBs in Patients Infected With COVID-19

An even larger number of trials are testing the hypothesis that RAAS blockers, and in particular ARBs, might be beneficial in patients with SARS-CoV-2 pneumonia. We have identified 21 such trials, listed in Supplementary Table 5. For instance, two trials sponsored by the University of Minnesota (United States) are testing the efficiency of losartan in patients with COVID-19 either requiring hospitalization (assessing the respiratory severity at day 7, NCT04312009) or not requiring hospitalization (assessing the rate of hospital admission within 15 days of randomization, NCT04311177). Other trials are focusing on elderly patients: two French trials are assessing the effectiveness of telmisartan in elderly hospitalized patients (COVID-Aging, coordinated in Strasbourg, evaluating the 2-week survival rate, NCT04359953) or in outpatients (COVERAGE, coordinated in Bordeaux, evaluating a composite of hospitalization or death at day 14, NCT04356495). Other ongoing trials worldwide are listed in Supplementary Table 5.

## Conclusion

**FIGURE 2:**
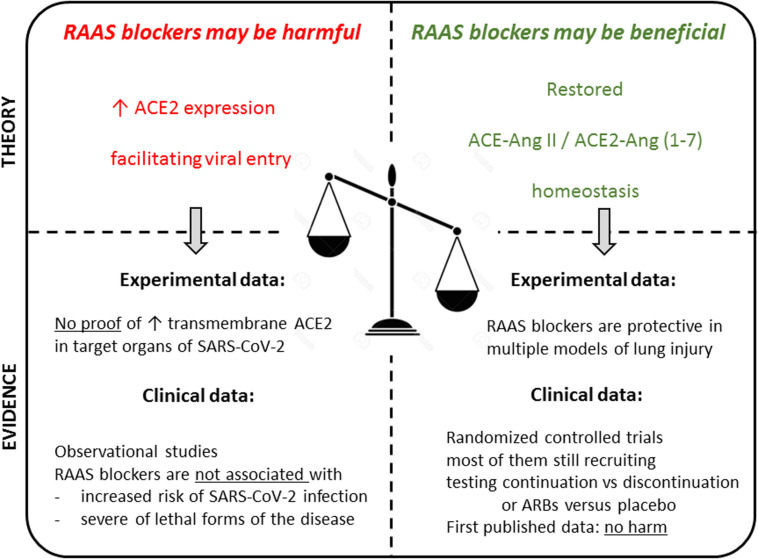
Summarizing illustration. Theoretical pros and cons in the debate on the use of RAAS blockers in the context of the COVID-19 pandemic, and available experimental and clinical evidence to date. Overall, there are no evidence-based data supporting the discontinuation of these medications in the general population, or in patients with COVID-19. In hospitalized patients, these drugs should be managed according to usual clinical practice, taking into account hemodynamics and kidney function.

Whereas an increased tissue expression of ACE2, either due to underlying conditions or to pharmacological treatment, might potentially facilitate SARS-CoV-2 infection and/or more severe forms of the disease on the one hand, on the other hand a higher ACE2 activity could be beneficial in infected patients by attenuating lung injury and inflammation. Because of these opposite potential effects of ACE2 on the disease (Wang K. et al., 2020), the role of RAAS blockers, which may modulate ACE2 expression and activity, is controversial (Figure 2).

Several large studies seem to have ruled out that chronic exposure to RAAS blockers may predispose patients to infection, while studies on the association between exposure to RAAS blockers and severity of the disease in patients with COVID-19 have yielded more discrepant results. However, these apparent discrepancies are largely explained by disparities in study design, exposure measurement, adjustment methodologies, and often small sample size. Properly designed studies are also reassuring and consistently found no significant association between chronic exposure to RAAS blockers and outcome in infected patients. Preliminary results from randomized clinical trials did not raise concern regarding the continued use of the treatments in hospitalized patients (Figure 2).

Overall, available data corroborate statements from scientific societies against the preventive discontinuation of RAAS blockers in the general population, while management of these medications in infected patients, especially in hospitalized patients, should be clarified by the results of ongoing studies.

Despite extensive worldwide research since the discovery of ACE2 in 2000, the SARS outbreak in 2003, and the COVID-19 pandemic since December 2019, many gaps in knowledge remain regarding the regulation of ACE2 and its implications in the pathogenesis of SARS-CoV-2. The discrepancies in the literature highlight the need for integrated translational research projects, from molecular biology to animal and human pathophysiology, especially as many potential therapeutic targets will be directly impacted by a better knowledge of ACE2, a double-edged sword against the virus.

## Author Contributions

EV-P conceived the concept of the manuscript, drew the figures, and revised the manuscript and tables. SBG wrote the first draft of the manuscript and tables. All authors contributed to the literature review, wrote iterative drafts of manuscript and tables, and approved the final version.

## Conflict of Interest

The authors declare that the research was conducted in the absence of any commercial or financial relationships that could be construed as a potential conflict of interest.

## Supplementary Material

Supplementary Table 1 Influence of pharmacological blockade of the renin-angiotensin system on the expression of ACE2: animal models.

Supplementary Table 2 Influence of the pharmacological blockade of the renin-angiotensin system on the expression of ACE2: human studies.

Supplementary Table 3 Representative selection of large-scaled studies on the association between cardiovascular or metabolic comorbidities and outcome in COVID-19.

Supplementary Table 4 Observational studies evaluating the impact of ACEIs/ARBs on the risk of a positive COVID-19 test **(A)** and on the course of the disease in infected patients **(B)**.

Supplementary Table 5 Ongoing clinical trials (last update: January 25th 2021).
