# Supplementary material for: Controversial Roles of the Renin Angiotensin System and Its Modulators During the COVID-19 Pandemic
Source: Front Physiol. 2021 Feb 22;12:624052. doi: 10.3389/fphys.2021.624052 (PMC7937723; doi:10.3389/fphys.2021.624052)
Supplement: Supplementary Table 1 — Influence of pharmacological blockade of the renin-angiotensin system on the expression of ACE2: animal models. [file Table_1.docx]

**Supplementary material**

**Controversial roles of the renin angiotensin system and its modulators**

**during the COVID-19 pandemic**

Simon B. Gressens, Georges Leftheriotis, Jean-Claude Dussaule, Martin Flamant, Bernard I. Levy and Emmanuelle Vidal-Petiot

**Content:**

[**Supplementary Table 1**. Influence of pharmacological blockade of the renin-angiotensin system on the expression of ACE2: animal models 2](#_Toc63356497)

[A. Studies reporting the effect RAAS blockers including in wild type or control animals 2](#_Toc63356498)

[B. Studies reporting the effect RAAS blockers only in disease models 7](#_Toc63356499)

[**Supplementary Table 2.** Influence of the pharmacological blockade of the renin-angiotensin system on the expression of ACE2: human studies 12](#_Toc63356500)

[**Supplementary Table 3.** Representative selection of large-scaled studies on the association between cardiovascular or metabolic comorbidities and outcome in COVID-19 18](#_Toc63356501)

[**Supplementary Table 4.** Observational studies evaluating the impact of ACEIs/ARBs on the risk of a positive COVID-19 test (A) and on the course of the disease in infected patients (B) 27](#_Toc63356502)

[A. Impact of ACEIs/ARBs on the risk of a positive COVID-19 test 27](#_Toc63356503)

[B. Impact of ACEIs/ARBs on the course of the disease in infected patients 31](#_Toc63356504)

[**Supplementary Table 5.** Ongoing clinical trials (*last update: January 25^th^ 2021)* 48](#_Toc63356505)

# Supplementary Table 1. Influence of pharmacological blockade of the renin-angiotensin system on the expression of ACE2: animal models

## Studies reporting the effect RAAS blockers including in wild type or control animals

| **Study** | **Species (strain)** | **Tissue(s)** | **Model / Disease** | **Disease versus control condition** | | | **Effect of ACEI^∇^ / ARB^⊗^** | | |
| --- | --- | --- | --- | --- | --- | --- | --- | --- | --- |
|  |  |  |  | **mRNA level** | **Protein**  **Expression** | **Enzyme**  **Activity.** | **mRNA level** | **Protein expression** | **Enzymatic activity** |
| Ferrario – 2005  Circulation  (Ferrario et al., 2005a) | Rat (Lewis) | Heart | WT | - | - | - | ACE ↔**^∇ ⊗^**  ACE2 ↑**^∇ ⊗^**  *Stronger effect with ACEI than ARB*  ACE2 ↔**^∇+⊗^** | - | ACE2 ↑**^⊗^**  ACE2 ↔**^∇^**  ACE2↑**^∇+⊗^** |
| Ferrario – 2005  Kidney Int  (Ferrario et al., 2005b) | Rat (Lewis) | Kidney | WT | - | - | - | ACE2 ↔**^∇ ⊗^** | - | ACE2 ↑**^∇^** **^⊗^**  ACE2↔**^∇+⊗^** |
| Ocaranza – 2006  Hypertension  (Ocaranza et al., 2006) | Rat (Sprague-Dawley) | Heart | Myocardial Infarction (LCA ligation) | At 8 weeks  ACE ↑  ACE2 ↓ | - | At 1 week  ACE ↑  ACE2 ↑  At 8 weeks  ACE ↑  ACE2 ↓ | At 8 weeks  In sham-model:  ACE ↔**^∇^**  ACE2↔**^∇^**  In disease-model:  ACE ↓**^∇^**  ACE2 ↑**^∇^** | - | At 8 weeks  In sham-model:  ACE ↓**^∇^**  ACE2 ↑**^∇^**  In disease-model:  ACE ↓**^∇^**  ACE2 ↑**^∇^** |
|  |  | Plasma |  | - | - | At 1 week  ACE ↑  ACE2 ↑  At 8 weeks  ACE ↔  ACE2 ↓ | - | - | At 8 weeks  In sham model:  ACE ↓**^∇^**  ACE2 ↑**^∇^**  In disease model:  ACE ↓**^∇^**  ACE2 ↑**^∇^** |
| Hamming – 2008  Expl Physiol  (Hamming et al., 2008) | Rat (Wistar) | Kidney | WT | - | - | - | 2% NaCl diet:  ACE ↔**^∇^**  ACE2 ↔**^∇^** | - | 2% NaCl diet:  ACE ↓**^∇^**  ACE2 ↔**^∇^** |
|  |  |  |  |  |  |  | Low sodium (0.05% NaCl) diet:  ACE ↔**^∇^**  ACE2 ↓**^∇^** | - | Low sodium (0.05% NaCl) diet:  ACE ↓**^∇^**  ACE2 ↔**^∇^** |
| Soler – 2009  Am J Physiol Renal Physiol  (Soler et al., 2009) | Mouse  (C57BLKS/J) | Kidney  (renal arteriole) | WT | - | - | - | ACE ↔**^⊗^**  ACE2 ↑**^⊗^**  ACE/ACE2 ↓**^⊗^** | ACE ↔**^⊗^**  ACE2 ↑**^⊗^**  ACE/ACE2 ↓**^⊗^** | - |
| Han – 2010  Toxicol Appl Pharmacol  (Han et al., 2010) | Rat (Sprague-Dawley) | Lung | Smoke exposure-induced pulmonary arterial hypertension | - | ACE ↑  ACE2 ↓ | - | - | In controls:  ACE ↔**^⊗^**  ACE2 ↔**^⊗^**  In disease model:  ACE ↔**^⊗^**  ACE2 ↑**^⊗^** | - |
| Velkoska – 2010  Clin Sci  (Velkoska et al., 2010) | Rat (Sprague-Dawley) | Kidney | Partial nephrectomy | ACE ↓  ACE2 ↓^1^ | ACE ↑ | ACE2 ↓ | In WT:  ACE ↔  ACE2 ↔  In disease model:  ACE ↔**^∇^**  ACE2 ↔**^∇^** | In controls:  ACE ↓**^∇^**  In disease model:  ACE ↓**^∇^** | In controls:  ACE2 ↔**^∇^**  In disease model:  ACE2 ↑**^∇^** |
| Wösten-van Asperen – 2011  J Pathol  (Wösten-van Asperen et al., 2011) | Rat (Sprague-Dawley) | Lung^2^ | Mechanical ventilation (MV) and LPS-induced lung injury | - | MV alone:  ACE ↑  ACE2 ↑  MV and Endotracheal LPS-induced ARDS:  ACE ↑  ACE2 ↑*  *: *may result from proteolytic degradation of ACE2 in lavage fluid* | MV alone:  ACE ↑  ACE2 ↔  ACE/ACE2 ↔  Endotracheal LPS alone:  ACE ↑  ACE2 ↔  ACE/ACE2 ↔  MV and Endotracheal LPS-induced ARDS:  ACE ↑ (*Stronger effect than with MV or LPS alone*)  ACE2 ↓  ACE/ACE2 ↑ | - |  | MV alone:  ACE ↔**^⊗^**  ACE2 ↔**^⊗^**  ACE/ACE2 ↔**^⊗^**  MV and Endotracheal LPS-induced ARDS:  ACE ↔**^⊗^**  ACE2 ↑**^⊗^**  ACE/ACE2 ↓**^⊗^** |
| Li – 2015  Shock  (Li et al., 2015) | Rat (Sprague-Dawley) | Lung | Intravenous LPS-induced acute lung injury | - | ACE ↑  ACE2 ↓  ACE/ACE2 ↑ | - | - | Non-LPS exposed:  ACE ↔**^∇^**  ACE2 ↑**^∇^**  LPS-exposed:  ACE ↓**^∇^**  ACE2 ↑**^∇^**  ACE/ACE2 ↓**^∇^** | - |
| Wu - 2020  Hypertension  (Wu et al., 2020) | Mouse (C57BL/6J) | Lung, ileum, kidney, heart | WT | - | - | - | ACE2 ↔**^∇⊗^** | ACE2 ↔**^∇⊗^** | ACE2 ↔**^∇^**  ACE2 ↔**^⊗^** |
| Wysocki – 2020  J Am Soc Nephrol  (Wysocki et al., 2020) | Mouse (C57BL/6J) | Kidney, Lung | WT | - | - | - | Kidney cortex lysates  ACE2 ↔**^∇⊗^** | Kidney cortex lysates  ACE2 ↔**^∇⊗^**  Kidney isolated membranes  ACE2 ↓**^∇⊗^**  Cytosolic expression  ACE2 ↑**^∇^**  *(suggesting internalization of the protein)* | Kidney cortex lysates  ACE2 ↔**^∇^**  Total lung lysates  ACE2 ↔**^∇⊗^**  Lung isolated membranes  ACE2 ↔**^∇⊗^** |

## Studies reporting the effect RAAS blockers only in disease models

| **Study** | **Species (strain)** | **Tissue(s)** | **Model / Disease** | **Disease versus control condition** | | | **Effect of ACEI^∇^ / ARB^⊗^** | | |
| --- | --- | --- | --- | --- | --- | --- | --- | --- | --- |
|  |  |  |  | **mRNA** | **Protein**  **Expression** | **Enzyme**  **Activity** | **mRNA** | **Protein expression** | **Enzymatic activity** |
| Burrell – 2005  Eur Heart J  (Burrell et al., 2005) | Rat (Sprague-Dawley) | Heart: infarcted myocardium and border | Myocardial Infarction (LCA ligation) | At day 1/3/28  ACE ↔/↑/↔  ACE2 ↔/↑/↔  *Each compared to previous timepoint* | At day 28  ACE ↑ | At day 28  ACE2 ↑ | At day 28  ACE ↓**^∇^**  ACE2 ↔**^∇^** | At day 28  ACE ↓**^∇^**  *Of note: human ischemic heart samples showed an increased in ACE and ACE2 protein expression compared to non-ischemic samples* | At day 28  ACE2 ↔**^∇^** |
|  |  | Heart: viable myocardium |  | At day 1/3/28  ACE ↔/↔/↑  ACE2 ↔/↔/↑  *Each compared to previous timepoint* | At day 28  ACE ↑ | At day 28  ACE2 ↑ | At day 28  ACE ↓**^∇^**  ACE2 ↔**^∇^** | At day 28  ACE ↓**^∇^** | At day 28  ACE2 ↔**^∇^** |
| Ishiyama – 2004  Hypertension  (Ishiyama et al., 2004) | Rat (Lewis) | Heart | Myocardial Infarction (LCA ligation) | ACE ↔  ACE2 ↔ | - | - | ACE ↔**^⊗^**  ACE2 ↑**^⊗^** | - | - |
| Igase – 2005  Am J Physiol Heart Circ Physiol  (Igase et al., 2005) | Rat (SHR) | Aorta | Hypertension | - | - | - | ACE2 ↑**^⊗^** | ACE2 ↑**^⊗^** | ACE2 ↑**^⊗3^** |
| Karram – 2005  Heart Circ Physiol  (Karram et al., 2005) | Rat (Wistar) | Heart | Heart failure (ACF) | - | At 2 weeks  ACE ↑  ACE2 ↓  At 4 weeks  ACE ↑  ACE2 ↓ | At 2 weeks  ACE ↔  ACE2 ↔ | - | At 2 weeks  ACE ↓**^⊗^**  ACE2 ↑**^⊗^**  At 4 weeks  ACE ↓**^⊗^**  ACE2 ↑**^⊗^** | At 2 weeks  ACE ↔**^⊗^**  ACE2 ↑**^⊗^** |
| Agata – 2006  Hypertension Res  (Agata et al., 2006) | Rat (SHRSP) | Heart | Hypertension | ACE2 ↔ | - | - | ACE2 ↑**^⊗^** | - | - |
|  |  | Kidney |  | - | ACE2 ↓ | - | - | ACE2 ↑**^⊗^** | - |
| Jessup – 2006  Am J Physiol Heart Circ Physiol  (Jessup et al., 2006) | Rat (Ren 2 transgenic Lewis) | Heart | Hypertension | - | - | - | ACE ↑**^∇⊗^**  ACE2 ↑**^∇⊗^** | - | ACE2 ↑**^∇⊗^** |
|  |  | Kidney |  | - | - | - | ACE ↑**^∇⊗^**  ACE2 ↑**^∇⊗^** | - | ACE2 ↑**^∇⊗^** |
| Takeda – 2007  Am J Hypertension  (Takeda et al., 2007) | Rat (Dahl) | Heart | Hypertension | High sodium diet (compared to low sodium diet):  ACE ↔  ACE2 ↓ | High sodium diet (compared to low sodium diet):  ACE ↔  ACE2 ↓ | - | High sodium diet (compared to low sodium diet):  ACE ↓**^⊗^**  ACE2 ↑**^⊗^** | High sodium diet (compared to low sodium diet):  ACE ↓**^⊗^**  ACE2 ↑**^⊗^** | - |
| Sukumaran – 2011  Int J Bio Sci  (Sukumaran et al., 2011) | Rat (Lewis) | Heart | Auto-immune Myocarditis | - | ACE2 ↓ | - | - | ACE2 ↑**^⊗^** | ACE2 ↑**^⊗4^** |
| Burrell – 2012  Exp Physiol  (Burrell et al., 2012) | Rat (Sprague-Dawley) | Kidney | Partial nephrectomy | ACE ↔  ACE2 ↓ | ACE ↓ | ACE2 ↓ | ACE ↓**^∇^**  ACE2 ↔**^∇^** | ACE ↓**^∇^** | ACE2 ↑**^∇^** (only in cortex) |
|  |  | Heart |  | ACE ↔  ACE2 ↔ | ACE ↔  ACE2 ↔ | ACE2 ↔ | ACE ↔**^∇^**  ACE2 ↔**^∇^** | ACE ↓**^∇^**  ACE2 ↔**^∇^** | ACE2 ↔**^∇^** |
| Burchill – 2012  Clin Sci  (Burchill et al., 2012) | Rat (Sprague-Dawley) | Heart^:^ infarcted myocardium and border | Myocardial Infarction (LCA ligation) | ACE ↑  ACE2 ↑ | ACE ↑  ACE2 ↑ | In plasma*:*  ACE ↔  ACE2 ↑ | ACE ↔**^∇⊗^**  ACE2 ↔**^∇⊗^** | ACE ↓**^∇^**  ACE ↔**^⊗^**  ACE2 ↔**^∇⊗^** | In plasma*:*  ACE ↓**^∇⊗^**  ACE2 ↓**^∇⊗^**  *Stronger effect with ACEI than ARB* |
|  |  | Heart^:^ viable myocardium |  | ACE ↔  ACE2 ↔ | ACE ↔  ACE2 ↑ |  | ACE ↔**^∇⊗^**  ACE2 ↔**^∇⊗^** | ACE ↓**^∇⊗^**  ACE2 ↔**^∇⊗^** |  |
| Sukumaran – 2012  Free Rad Res  (Sukumaran et al., 2012) | Rat (Lewis) | Heart | Auto-immune Myocarditis / Dilated Cardiomyopathy | ACE2 ↓ | ACE2 ↓ | - | ACE2 ↑**^⊗^** | ACE2 ↑**^⊗^** | ACE2 ↑**^⊗4^** |
| Yang – 2013  Arch Cardiovasc Dis  (Yang et al., 2013) | Rat (SHR) | Heart | Hypertension | ACE ↑  ACE2 ↓ | ACE ↑  ACE2 ↓ | - | ACE ↓**^∇^**  ACE2 ↑**^∇^** | ACE ↓**^∇^**  ACE2 ↔**^∇^** | - |
| Zhang – 2014  Chin J Physiol  (Yanling Zhang et al., 2014) | Rat (Sprague-Dawley) | Heart | Renal ischemia | ACE ↑  ACE2 ↓ | ACE ↑  ACE2 ↓ | - | ACE ↓**^∇^**  ACE ↔**^⊗^**  ACE2 ↑**^∇⊗^** | ACE ↓**^∇^**  ACE ↔**^⊗^**  ACE2 ↑**^∇⊗^** | - |
| Wang – 2016  Int J Mol Med  (Wang et al., 2016) | Pig (Landrace) | Heart | Cardiac resuscitation | ACE ↑  ACE2 ↑  ACE/ACE2 ↔ | ACE ↑  ACE2 ↑  ACE/ACE2 ↔ | - | ACE ↓**^∇^**  ACE2 ↔**^∇^**  ACE/ACE2 ↓**^∇^** | ACE ↓**^∇^**  ACE2 ↔**^∇^**  ACE/ACE2 ↓**^∇^** | - |
| Lezama-Martinez – 2018  J Cardiovasc Phar  (Lezama-Martinez et al., 2018) | Rat (SHR) | Aorta | Hypertension | ACE ↑  ACE2 ↑  ACE/ACE2 ↓ | - | - | ACE ↓**^∇⊗^**  ACE2 ↓**^∇⊗^**  ACE/ACE2 ↑**^∇<⊗^** | - | - |

When reported, the expression of ACE is also reported. In experimental models which are disease models, modifications of ACE2 and ACE expressions in disease versus control condition is also reported in the “disease versus control condition” column.

ACEI: ACE inhibitor, ARB: angiotensin II receptor blocker; ACE: angiotensin converting enzyme; ACF: aortocaval fistula, ARDS: acute respiratory distress syndrome, LCA: left coronary artery, LPS: lipopolysaccharide, SHR: spontaneously hypertensive rats, SHRSP:  spontaneously hypertensive stroke prone rat,

WT: wild-type

↑, ↓ and ↔ stand for increase, decrease and no significant variation respectively

_♥_ expression in the heart

**∇+⊗**: effect of ARB and ACEI used in combination

When reported or easily computable from available data, ACE/ACE2 ratio (mRNA, protein expression, or activity) is also reported

^1^ results shown for cortex tissue (no modification of ACE2 mRNA after partial nephrectomy)

^2^results reported here regarding enzymatic activity are measured in bronchoalveolar lavage fluid

^3^ACE2 activity was indirectly assessed from Ang1-7 protein concentration in aorta tissues

^4^ACE2 activity was indirectly assessed from Ang1-7 protein concentration in cardiac tissues

# Supplementary Table 2. Influence of the pharmacological blockade of the renin-angiotensin system on the expression of ACE2: human studies

| **Study** | **Population** | **Tissue(s)** | **Disease** | **Results / Comment** |
| --- | --- | --- | --- | --- |
| Lely – 2004  J Pathol  (Lely et al., 2004) | Renal biopsies (n=58)  Normal parts of kidney from nephrectomy for renal tumors as controls (n=18) | Renal tissues | Primary and secondary renal diseases | Expression distribution of ACE2 protein was uniform across renal disorders:  Tubules and blood vessels ACE2 ↔ compared to controls  Glomeruli epithelium ACE2 ↑ compared to controls  Glomerular capillary endothelium and peritubular capillaries ACE2 ↑ compared to controls  Among membranous glomerulopathies:  **Effect of ACEI on ACE2 protein expression: ↔** |
| Epelman – 2008  JACC  (Epelman et al., 2008) | Patients suspected of heart failure (n=258) | Plasma | Heart failure | Disease effect on ACE2 plasma activity: ↑  **Effect of ACEI/ARB on ACE2 plasma activity: ↔**  ***Severity of heart failure was strongly associated with plasma ACE2 activity*** |
| Reich – 2008  Kidney Int  (Reich et al., 2008) | Renal biopsies from diabetic patients (n=13, routine clinical diagnostic investigation), chronic allograft nephropathy / primary focal segmental glomerulosclerosis (n=22)  Healthy controls (n=8, living donors) | Renal tubules and glomeruli | Diabetes | mRNA expression  Disease effect in tubules and glomeruli: ACE2 ↓  Disease effect in tubules and glomeruli: ACE ↑  **Effect of ACEI/ARB on ACE2: ↔**  Protein expression  Disease effect in tubules: ACE2↓  Disease effect in glomeruli: protein levels lower than the limit of detection in both groups (controls and diabetic patients)  *NB: all diabetic patients had history of hypertension* |
|  |  |  | Chronic allograft nephropathy / Primary focal segmental glomerulosclerosis | mRNA expression  Disease effect in tubules and glomeruli: ACE2 ↔ / ACE ↔  **Effect of ACEI/ARB on ACE2: ↑** (in tubules only)  Protein expression  Disease effect in tubules and glomeruli: ACE2 ↔ / ACE ↔ |
| Mizuiri – 2011  Nephrology  (Mizuiri et al., 2011) | CKD patients (n=190) and healthy controls (n=36) | Urine | CKD | Disease effect on protein concentration in urine   - CKD effect compared to healthy donors: ACE2 ↑ - Diabetic nephropathy compared to others CKD: ACE2 ↑   **ACEI/ARB effect on protein concentration in urine: ACE2 ↔** |
| Soro-Paavonen – 2012  J Hypertension  (Soro-Paavonen et al., 2012) | Type 1 diabetic patients (n=859) – healthy controls (n=204) | Plasma | Diabetes | Gender effect on serum ACE2 activity  Men > Women (even among healthy controls)  Disease effect on serum ACE2 activity   - Without microalbuminuria: ACE2 ↔ - With micro- or macroalbuminuria: ACE2 ↑ only among men - With associated coronary heart disease: ACE2↑   **Effect of ACEI on ACE2 serum activity among diabetic patients: ↑**  **Effect of ARBs on ACE2 serum activity among diabetic patients: ↑only among women** |
| Ortiz-Perez – 2013  PLoS One  (Ortiz-Pérez et al., 2013) | Patients with STEMI (n=88)  Controls without cardiovascular disease (n=22) | Plasma | STEMI | Disease effect on ACE2 serum activity  STEMI at baseline: ACE2 ↑  STEMI at day 7 compared to baseline: ACE2 ↑  **Effect of ACEI on ACE2 serum activity at baseline and at day 7: ↔** |
| Vuille-dit-Bille – 2015  Amino Acids  (Vuille-dit-Bille et al., 2015) | Humans with long term-use of ACEI (n=9) or ARB (n=13) and controls (n=24) | Intestine | Healthy | **Effect of ACEI on ACE2 mRNA expression in duodenum: ↑**  **Effect of ARB on ACE2 mRNA expression in duodenum: ↔** |
| Anguiano – 2015  Nephrol Dial Transplant  (Anguiano et al., 2015) | CKD patients without history of cardiovascular disease (except hypertension) (n=2572) including controls (n=568, eGFR>60mL/min) | Plasma | CKD | Disease effect on plasma activity  Decreased eGFR: ACE2 ↓  Decreased eGFR: ACE ↑  **Effect of ACEI on ACE and ACE2 plasma activities: ACE2 ↔ / ACE** ↓  **Effect of ARB on ACE and ACE2 plasma activities depending on eGFR**  **>60 mL/min: ACE2 ↑ / ACE ↑**  **<60 mL/min not requiring dialysis: ACE2 ↔ / ACE ↑**  **<60 mL/min requiring dialysis: ACE2 ↑ / ACE ↔** |
| Furuhashi – 2015  Am J Hypertens  (Furuhashi et al., 2015) | Hypertensive patients (n=100)  Controls declared as comorbidity and medication free (n=101) | Urine | Hypertension | Anti-hypertension medication effect on protein concentration in urine  **Olmesartan (ARB): ACE2 ↑**  **Other ARBs (losartan, candesartan, valsartan, telmisartan): ACE2 ↔**  **ACEI (enalapril): ACE2 ↔**  Calcium channel blocker: ACE2 ↔ |
| Liang – 2015  Kidney Blood Press Res  (Liang et al., 2015) | Diabetes mellitus type 2 (n=132)  Healthy controls, volunteers (n=34) | Urine | Diabetes  Hypertension | Disease effect on protein concentration (and enzymatic activity) in urine  Diabetes: ACE2 ↑  Associated hypertension: ACE2 ↑  Importance of proteinuria: ACE2 ↔  Elevated HbA1C ↑ ACE2  **Effect of ACEI/ARB on ACE2 protein concentration in urine among hypertensive diabetic patients: ↓**  *78/132 diabetic patients had a history of hypertension* |
| Mariana – 2016  Int Urol Nephrol  (Mariana et al., 2016) | Diabetic patients (n=75) | Urine | Diabetes | **Effect of ACEI/ARB on ACE2 protein concentration in urine**: **↔**  *most patients had preserved eGFR (>80 mL/min)*  *77.3% were treated with an ACEI/ARB* |
| Úri – 2016  J Renin Angiotensin Aldosterone Syst  (Uri et al., 2016) | Heart failure patients (n=188)  Hypertensive patients without heart failure (n=239)  Healthy controls (n=45) | Plasma | Hypertension  Heart failure | Disease effect on serum ACE 2 activity  Hypertension: ACE2 ↑  Systolic left ventricular dysfunction ACE2 ↑  **ACEI/ARB effect on serum ACE2 activity: ↔** |
| Walters – 2016  Europace  (Walters et al., 2016) | Patients with atrial fibrillation (n=78) and controls without atrial fibrillation (n=25) | Plasma | Atrial fibrillation | Disease effect on plasma activity: ACE2 ↑  **Effect of ACEI/ARB on plasma ACE2 activity: ↔** |
| Ramchand – 2018  PLoS One  (Ramchand et al., 2018) | Patients with coronary artery disease (n=79) | Plasma | Coronary heart disease | Gender effect on plasmatic ACE2 activity  Men > Women  **Effect of ACEI/ARB on ACE2 plasma activity: ↔**  *Elevated plasma ACE2 activity was associated with an increased risk of major cardiovascular events* |
| Chirinos - 2020  Hypertension  (Chirinos et al., 2020) | Patients with chronic heart failure (n=2248) | Plasma | Heart failure | **Effect of ACEI/ARB on ACE2 plasma concentration: ↔**  In Heart failure, low ACE2 plasma concentration was associated with: older age, male sex, diabetes mellitus, lower renal function, worse NYHA class, higher pro-BNP |
| Jiang – 2020  Eur Heart J  (Jiang et al., 2020) | Human kidney samples from different datasets (n=534)  Non-renal tissue samples from GTEx project database | Renal tissues | General population | Transcriptome analysis (RNA-sequencing) of renal ACE2 gene expression:  Gender effect on ACE2 mRNA expression in kidney tissues: men > women  Effect of age on ACE2 mRNA expression in kidney and lung tissues: ↑  Decreased eGFR on ACE2 mRNA expression in kidney tissues: ↑  Effect of hypertension, diabetes, obesity on ACE2 mRNA expression in kidney tissues: ↔  **Effect of ACEI/ARB on ACE2 mRNA expression in kidney tissues: ↔** |
| Narula – 2020  Lancet  (Narula et al., 2020) | Subcohort of hypertensive patients from the PURE cohort (n=5216) | Plasma | Hypertension | **Association of ACEI or ARB with circulating ACE2 plasma concentration: ↔** |
| Ramchand – 2020  JACC Cardiovasc Imaging  (Ramchand et al., 2020) | Patients with aortic stenosis (n=127) | Plasma | Aortic stenosis | Gender effect on plasmatic ACE2 activity  Men > Women  **Effect of ACEI/ARB on ACE2 plasma activity: ACE2 ↔**  *NB myocardial tissue was available in 22 patients*  *ACE2 plasmatic activity inversely correlated with myocardial ACE2 mRNA expression* |
| Stegbauer – 2020  Hypertension  (Stegbauer Johannes et al., 2020) | Patients with severe aortic stenosis (n=41), severe mitral regurgitation (and controls, n=17) | Myocardial samples (obtained during valvular surgery) | Severe aortic stenosis  Severe mitral regurgitation | Effect of severe aortic stenosis (compared to healthy controls) on ACE2 protein expression in cardiomyocytes: ACE2 ↑  Effect of severe aortic stenosis (compared to healthy controls) on ACE2 mRNA expression in cardiomyocytes: ACE2 ↑  *ACE2 mRNA expression was corelated to protein expression among patients with severe aortic stenosis*  Effect of severe mitral regurgitation (compared to healthy controls) on ACE2 protein expression in cardiomyocytes: ACE2 ↔  **Effect of ACEI on ACE2 protein expression in cardiomyocytes: ACE2 ↑**  **Effect of ARB on ACE2 protein expression in cardiomyocytes: ACE2 ↔** |
| Sama – 2020  Eur Heart J  (Sama et al., 2020) | Heart failure patients (2 cohorts: index, n=2022 and validation, n=1698) | Plasma | Heart failure | Index cohort  **Effect of ACEI on ACE2 plasma concentration: ACE2 ↔**  **Effect of ARB on ACE2 plasma concentration: ACE2 ↔**  Validation cohort  **Effect of ACEI on ACE2 plasma concentration: ACE2 ↓**  **Effect of ARB on ACE2 plasma concentration: ACE2 ↓** |

CKD: chronic kidney disease, eGFR: estimated glomerular filtration rate, STEMI: ST elevation myocardial infarction, ACEI: angiotensin converting enzyme inhibitor, ARB: Angiotensin II receptor blocker

# Supplementary Table 3. Representative selection of large-scaled studies on the association between cardiovascular or metabolic comorbidities and outcome in COVID-19

| **Study** | **Country** | **Number of patients** | **Age**  **(years)** | **Outcome** | **Hypertension** | **Diabetes** | **Chronic Kidney Disease** | **Other cardiovascular disease** |
| --- | --- | --- | --- | --- | --- | --- | --- | --- |
| Albitar – 2020  Diab Res Clin Pract  (Albitar et al., 2020) | Open access database | 828 | 49 (Mn) | Death | 10.9 %  univariate p<0.001  aOR 3.58 [1.69 ; 7.55] | 7.5 %  univariate p<0.001  aOR 12.23 [4.13 ; 32.27] | 1.9 %  univariate p<0.001  aOR ns | 2.8 %  univariate p<0.001  aOR ns |
| Berenguer – 2020  Clin Microbiol Infect  (Berenguer et al., 2020) | Spain | 4035 | 70 (Md) | Death | 51.2 %  uHR 2.55 [2.23 ; 2.91]  aHR 1.22 [1.05 ; 1.40] | 21.8 %  aHR ns | 5.0 %  uHR 2.7 [2.36 ; 3.09]  aHR 1.41 [1.22 ; 1.63] | Chronic Heart Disease 23.3 %  aHR ns |
| Boulle – 2020  Clin Infect Dis  (Boulle et al., 2020) | South Africa | 15 203 | - | Hospitalization | 21.5 %  aHR 1.02 [0.84 ; 1.24] | 13.6 %  aHR at least 2.02 [1.47 ; 2.76] | 2.5 %  aHR 1.92 [1.51 ; 2.45] | - |
|  |  | 2978 |  | Death | 42.5 %  aHR 1.05 [0.88 ; 1.27] | 40.4 %  aHR at least 1.13 [0.83 . 1.55] | 9.0 %  aHR 1.51 [1.20 ; 1.89] | - |
| Bravi – 2020  PLoS One  (Bravi et al., 2020) | Italy | 1603 | 58 (Mn) | Death | 33.9 %  uOR NA  aOR 1.39 [0.94 ; 2.05] | 12.1 %  uOR NA  aOR 1.58 [1.06 ; 2.34] | 5.4 %  uOR NA  aOR 1.13 [0.64 ; 1.99] | 16.1 %  uOR NA  aOR 1.39 [0.71 ; 1.56] |
| Cariou – 2020  Diabetologia  (Cariou et al., 2020) | France | 1317 | 69.8 (Md) | Death^1^ | 77.2 %  uOR 1.82 [1.11 ; 2.98]  aOR ns | 100 % (diabetic patients cohort)  NA | 33.3 %  uOR 3.19 [2.09 ; 4.87]  aOR 2.14 [1.16 ; 3.94] | CAD 26.9 %  uOR 2.65 [1.84 ; 3.82]  aOR 2.54 [1.44 ; 4.50] |
| Cen – 2020  Clin Microbiol Infect  (Cen et al., 2020) | China | 1007 | 61 (Md) | Progression in severity of the disease according to Interim Guidelines for COVID-19 of China (6^th^ edition) | 26.8 %  uHR 2.540 [2.01 ; 3.21]  aHR 1.44 [1.11 ; 1.88] | 11.8 %  uHR 2.92 [2.22 ; 3.86]  aHR 1.82 [1.35 ; 2.44] | 1.4 %  uHR 3.06 [ 1.51 ; 6.17]  aHR ns | CAD 6.5 %  uHR 2.46 [1.72 ; 3.52]  aHR 1.83 [1.26 ; 2.66] |
| Chen – 2020  Clin Infect Dis  (Chen et al., 2020a) | China | 3309 | 62 (Md) | Death | 29.9 %  uOR 1.53 [1.20 ; 1.95]  aOR 1.14 [0.87;1.50] | 14 %  uOR 0.97 [0.70 ; 1.36]  aOR– | 1.7 %  uOR 2.67 [1.40 ; 5.11]  aOR 2.85 [1.42 ; 5.73] | 7.3%  uOR 1.94 [1.34 ; 2.80]  aOR 1.41 [0.94 ; 2.13] |
| Chen – 2020  Leukemia  (Chen et al., 2020b) | China | 1859 | 59 (Md) | Death | 31 %  uHR 2.21 [1.68 ; 2.90]  aHR ns | 14 %  uHR 2.47 [1.82 ; 3.34]  aHR ns | 2 %  uHR ns | 14 %  uHR 2.56 [1.90 ; 3.45]  aHR ns |
| Cunningham – 2020  JAMA Intern Med  (Cunningham et al., 2020) | USA | 3222 | 28 (Mn) | Death or ventilation | 16.1 %  up<0.001  aOR 2.36 [1.79 ; 3.12] | 18.2 %  up<0.001  aOR 1.31 [0.99 ; 1.73] | - | - |
| Fried – 2020  Clin Infect Dis  (Fried et al., 2020) | USA | 11721 |  | Mechanical ventilation | 46.7 %  aOR 0.82 [0.72 ; 0.92] | 27.8 %  aOR 0.97 [0.80 ; 1.16] | 12.2 %  aOR 1.22 [1.05 ; 1.43] | 18.6 %  aOR 1.22 [1.06 ; 1.41] |
|  |  |  |  | Death | aOR 0.78 [0.69 ; 0.88] | aOR 0.91 [0.77 ; 1.08] | aOR 1.66 [1.45 ; 1.91] | aOR 1.44 [1.27 ; 1.63] |
| Giorgi Rossi – 2020  PLoS One  (Giorgi Rossi et al., 2020) | Italy | 2653 | 63 (Mn) | Hospitalization | 18.1 %  aHR 1.4 [1.2 ; 1.6] | 12.0 %  aHR 1.5 [1.3 ; 1.9] | 2.5 %  aHR 1.9 [1.3 ; 2.9] | CAD 7.1 %  1.3 [1.0 ;1.7]  CHF 5.8 %  aHR 1.6 [1.2 ; 2.1] |
|  |  |  |  | Death | aHR 1.6 [1.2 ; 2.1] | aHR 1.6 [1.1 ; 2.2] | aHR 1.5 [0.9 ; 2.6] | CAD  aHR 1.7 [1.2 ; 2.5]  CHF  aHR 2.3 [2.6 ; 3.2] |
| Gottlieb – 2020  Acad Emerg Med  (Gottlieb et al., 2020) | USA | 8673 | 41 (Md) | Hospitalization | 14.6 %  aOR 1.84 [1.53 ; 2.22] | 22.1 %  aOR 1.77 [1.46 ;2.16] | 4.4 %  aOR 2.60 [1.77 ; 2.61] | CAD 3.7 %  aOR 1.45 [1.03 ; 2.06]  CHF 3.2 %  aOR 1.79 [1.23 ; 2.61] |
|  |  |  |  | Critical Illness | aOR 1.23 [0.91 ; 1.67] | aOR 1.21 [0.93 ; 1.58] | aOR 0.89 [0.60 ; 1.32] | CHF  aOR 1.45 [1.00 ; 2.21] |
| Gupta – 2020  JAMA Intern Med  (Gupta et al., 2020) | USA | 2215 | 60.5 (Mn) | Death | 59.7 %  uNA  aOR 1.06 [0.83 ; 1.06] | 38.9 %  uNA  aOR 1.14 [0.91 ; 1.43] | 10.3 %  uNA  aNA | CAD aOR 1.47 [1.07 ; 2.02]  CHF aOR 1.08 [0.75 ; 1.58] |
| Hernandez-Galdamez – 2020  Arch Med Res  (Hernández-Galdamez et al., 2020) | Mexico | 211 003 | 45.7 (Mn) | ICU admission | 20.12 %  univariate p<0.001  aOR 1.08 [1.01 ; 1.16] | 16.4 %  univariate p<0.001  aOR 1.66 [1.56 ; 1.77] | 2.17 %  univariate p<0.001  aOR 1.12 [0.97 ; 1.29] | 2.35 %  univariate p<0.001  aOR 1.11 [0.97 ; 1.27] |
|  |  |  |  | Death | up<0.001  aOR 1.24 [1.20 ; 1.28] | up<0.001  aOR 1.69 [1.63 ; 1.74] | up<0.001  aOR 2.31 [2.15 ; 2.48] | up<0.001  aOR 0.93 [0.87 ; 1.00] |
| Iaccarino – 2020  Hypertension  (Iaccarino et al., 2020) | Italy | 1591 | 66.5 (Mn) | Death | 54.9 %  univariate p=0.0001  adjusted analysis ns | 16.9 %  univariate p=0.0001  adjusted analysis p=0.004 | 5.5 %  univariate p=0.0001  adjusted analysis p=0.004 | CAD 13.6 %  univariate p=0.0001 / adjusted analysis ns  CHF 11.8 %  univariate p=0.0001 / adjusted analysis ns |
| Ioannou – 2020  JAMA Netw Open  (Ioannou et al., 2020) | USA | 10 131 | 64 (Mn) | Hospitalization | 62.1 %  aHR 1.15 [1.05 ; 1.26] | 38.1 %  aHR 1.17 [1.08 ; 1.26] | 18.4 %  aHR 1.21 [1.11 ; 1.32] | CAD 21.7 %  aHR 1.04 [0.95 ; 1.13]  CHF 11.1 %  aHR 1.05 [0.95 ; 1.17] |
|  |  |  |  | Mechanical Ventilation | aHR 1.30 [1.03 ; 1.64] | aHR 1.40 [1.18 ; 1.67] | aHR 1.16 [0.96 ; 1.41] | CAD  aHR 0.95 [0.78 ; 1.15]  CHF  aHR 1.08 [0.86 ; 1.36] |
|  |  |  |  | Death | aHR 0.95 [0.81 ; 1.12] | aHR 1.13 [0.99 ; 1.29] | aHR 1.25 [1.08 ; 1.45] | CAD  aHR 1.02 [0.88 ; 1.18]  CHF  aHR 1.30 [1.10 ; 1.54] |
| Kim – 2020  Clin Infect Dis  (Kim et al., 2020) | USA | 2490 | 62 (Md) | ICU admission | 57.4 %  uRR 1.13 [1.01 ; 1.27]  aRR 0.92 [0.79 ; 1.07] | 39.2 %  uRR 1.20 [1.08 ; 1.34]  aRR 1.13 [1.03 ; 1.24] | 15.5 %  uRR 1.23 [1.11 ; 1.37]  aRR 1.05 [0.94 ; 1.16] | 34.6 %  uRR 1.17 [1.09 ; 1.26]  aRR 0.98 [0.88 ; 1.09] |
|  |  |  |  | Death | uRR 2.18 [1.66 ; 2.86]  aRR 1.07 [0.79 ; 1.45] | uRR 1.44 [1.20 ; 1.42]  aRR 1.19 [1.01 ; 1.40] | uRR 2.45 [2.04 ; 2.93]  aRR 1.33 [1.10 ; 1.61] | uRR 2.85 [2.42 ; 3.36]  aRR 1.28 [1.03 ; 1.58] |
| Liu – 2020  EClinicalMedicine  (Liu et al., 2020) | China | 2044 | 62 (Md) | Critical disease (mechanical ventilation, ICE admission, shock) | 39.7 %  Male  uOR 2.76 [1.97 ; 3.86]  aOR 2.05 [1.42 ; 2.95]  Female  uOR 1.42 [0.92 ; 2.18]  aOR 0.90 [0.56 ; 1.45] | 16.7 %  Male  uOR 1.39 [0.94 ; 2.06]  aOR –  Female  uOR 1.61 [0.94 ; 2.76]  aOR 1.32 [0.74 ; 2.35] | 1.6 %  Male  uOR 1.36 [0.44 ; 4.18]  aOR –  Female  uOR 4.28 [1.31 ; 13.92]  aOR 3.58 [0.98 ; 13.06] | CAD 9.8 %  Male  uOR 2.75 [1.76 ; 4.29]  aOR 1.65 [1.02 ; 2.66]  Female  uOR 1.40 [0.72 ; 2.73]  aOR - |
| Mikami – 2020  J Gen Intern Med  (Mikami et al., 2020) | USA | 3708^2^ | 66 (Md) | Death | 34.3 %  univariate NA  aHR 0.91 [0.79 ; 1.07] | 24.3 %  univariate NA  aHR 0.92 [0.73 ; 1.16] | 11.6 %  univariate NA  aHR 1.80 [1.60 ; 2.02] |  |
| Nachtigall – 2020  Clin Microbiol Infect  (Nachtigall et al., 2020) | Germany | 1904 | 73 (Md) | ICU admission | -^3^ | 15.2 %  aHR 1.46 [1.13 ; 1.88] | - | 36.1 %  aHR 1.26 [1.00 ; 1.58] |
|  |  |  |  | Death |  | aHR 1.02 [0.75 ; 1.38] |  | aHR 1.31 [1.02 ; 1.70] |
| Pan – 2020  Hypertension  (Pan et al., 2020) | China | 512^4^ | 68 (Md) | Death | aHR 2.24 [1.36 ; 3.70] | aHR 1.34 [0.78 ; 2.29] | aHR 1.09 [0.46 ; 2.59] | CAD  aHR0.90 [0.48 ; 1.70] |
| Parra-Bracamonte – 2020  Ann Epidemiol  (Parra-Bracamonte et al., 2020) | Mexico | 142 693 | 45 (Md) | Death | 20 %  uOR 3.52 [3.40 ; 3.65]  aOR 1.25 [ 1.19 ; 1.30] | 17 %  uOR 1.47 [1.41 ; 1.52] aOR 1.31 [1.25 ; 1.37] | 2 %  uOR 4.53 [4.20 ; 4.87]  aOR 1.85 [1.69 ; 2.03] | Cardiopathy 2 %  uOR 2.84 [2.63 ; 3.07]  aOR 1.01 [0.92 ; 1.11] |
| Perez-Guzman – 2020  Clin Infect Dis  (Perez-Guzman et al., 2020) | UK | 614 | 69 (Md) | Death | -  uOR 1.89 [1.32 ; 2.72]  aOR 1.26 [0.86 ; 1.86] | -  uOR 1.68 [1.16 ; 2.42]  aOR 1.47 [1.00 ; 2.16] | -  uOR2.55 [1.62 ; 4.01]  aOR 1.86 [1.15 ; 3.00] | CAD –  uOR 2.29 [1.39 ; 3.76]  aOR 1.45 [0.86 ; 2.44]  CHF –  uOR 1.99 [0.99 ; 4.00]  aOR 1.25 [0.61: 2.59] |
| Petrilli – 2020  BMJ  (Petrilli et al., 2020) | USA | 5279 | 63 (Md) | Critical Illness^5^ | 62 %  uOR 1.59 [1.34 ; 1.87]  aOR 0.96 [0.77 ; 1.2] | 34.7 %  uOR 1.38 [1.17 ; 1.62]  aOR 1.24 [1.03 ; 1.5]^6^ | 21.2 %  uOR 1.57 [1.30 ; 1.89]  aOR 1.07 [0.85 ; 1.3] | CHD 22 %  uOR 1.69 [1.41 ; 2.03]  aOR 0.96 [0.77-1.2] |
| Reilev – 2020  Int J Epidemiol  (Reilev et al., 2020) | Denmark | 11 122 | 46 (Md) | Hospitalization | 24 %  uOR 6.2 [5.6 ; 6.8]  aOR 1.7 [1.5 ; 1.9] | 7.9 %  uOR 4.6 [4.0 ; 5.3]  aOR 1.8 [1.6 ; 2.2] | 2.6 %  uOR 8.3 [6.5 ; 10.6]  aOR 2.9 [2.2 ; 3.9] | CAD 7.7 %  uOR 4.8 [4.2 ; 5.6]  aOR 1.4 [1.2 ; 1.7]  CHF 2.8 %  uOR 10.1 [7.9 ; 13.0]  aOR 2.6 [2.0 ; 3.4] |
|  |  |  |  | Death | uOR 9.2 [6.6 ; 11.1]  aOR 1.3 [1.1 ; 1.6] | uOR 4.4 [3.6 ; 5.4]  aOR 1.6 [1.3 ; 2.0] | uOR 7.8 [5.9 ; 10.2]  aOR 1.9 [1.4 ; 2.6] | CAD  uOR 5.2 [4.3 ; 6.4]  aOR 1.1 [0.9 ; 1.4]  CHF  uOR 10.2 [7.9 ; 13.2]  aOR 1.8 [1.3 ; 2.4] |
| Sands – 2020  Infect Control Hosp Epidemiol  (Sands et al., 2020) | USA | 6180 | 63 (Md) | Death | 63.4 %  aOR 0.63 [0.48 ; 0.82] | 39.1 %  aOR 1.57 [1.21 ; 2.03] | 19.1 % (including acute kidney injury?)  aOR 0.96 [0.71 ; 1.29] | CHF –  aOR 1.19 [0.90 ; 1.58] |
| Van Gerwen – 2020  J Med Virol  (van Gerwen et al., 2020) | USA | 2015 | 65 (Mn) | Death | 58.6 %  uOR 1.87 [1.53 ; 2.29]  aOR 1.08 [0.85 ; 1.37] | 39.5 %  uOR 1.62 [1.34 ; 1.96]  aOR 1.25 [1.00 ; 1.55] | 14.5 %  uOR 1.63 [1.26 ; 2.11]  aOR 1.16 [0.87 ; 1.56] | CAD 18.5 %  uOR 1.88 [1.40 ; 2.24]  aOR 0.97 [0.74 ; 1.28]  CHF 12.4 %  uOR 2.15 [1.64 ; 2.81]  aOR 1.47 [1.06 ; 2.02] |
| Wang – 2020  Clin Microbiol Infect  (Wang et al., 2020) | China | 1012 | 50 (Md) | Progression in disease severity | 4.5 %  univariate p=0.63  adjusted analysis NA | 2.7 %  univariate p=0.01  adjusted analysis NA | - | 2.5 %  univariate p<0.01  adjusted analysis NA |
| Williamson – 2020  Nature  (Williamson et al., 2020) | UK | 10 926^7^ | - | Death | aHR 1.09 [1.05 ;1.14]  fully aHR 0.89 [0.85 ; 0.93]^8^ | aHR 2.61 [2.46 ; 2.77]  fully aHR 1.95 [1.83 ; 2.08] | aHR 1.56 [1.38 ; 1.63]  fully aHR 1.33 [1.28 ; 1.40] | aHR 1.57 [1.41 ; 1.64]  fully aHR 1.17 [1.12 ; 1.22] |
| Yu – 2020  Am J Prev Med  (Yu et al., 2020) | China | 1663 | 64 (Md) | Death | 20.9 %  uOR 2.42 [1.77-3.32]  aOR 1.08 [0.68-1.72] | 14.4 %  uOR 3.77 [2.70-5.28]  aOR 2.34 [1.45-3.76] | 1.8 %  univariate ns | CHD 8.0 %  univariate ns |

Studies with less than 500 patients were excluded from the selection

uOR and uHR: unadjusted odds ratio and unadjusted hazard ratio respectively

aOR and aHR: adjusted odds ratio and adjusted hazard ratio respectively, RR: risk ratio

For each comorbidity, the prevalence, univariate analysis, and multivariate analysis are indicated.

Age is displayed as mean (Mn) or median (Md) according to available data.

Other cardiovascular diseases include (when detailed): coronary artery disease (CAD), congestive heart failure (CHF)

NS: non-significant (when the exact p-value was available it was provided in the table)

^1^outcome in this study was mortality at day 7. Displayed aOR for CKD and CHD were included in broader categories: microvascular and macrovascular complications respectively (corresponding OR displayed)

^2^the original study included 6493 patients, and among them 3708 were hospitalized. In-hospital death among hospitalized patients is the outcome displayed here

^3^Hypertension rate was not reported, and included in cardiovascular comorbidities

^4^ propensity score-matching cohorts were generated with a matching ratio of 1:1 (with and without hypertension) - each cohort included 256 patients

^5^ Critical illness was defined as a composite criterion including ICU admission, mechanical ventilation, death, and exclusive support care

^6^ NS after further adjustment for vital signs and laboratory analysis

^7^the authors evaluated associations with COVID-19-related death among a nationwide pseudonymized database, comparing COVID-19-death records (N=10 926) with the rest of the population in the database (N=17 278 392), regardless of SARS-CoV-2 testing. Hazard ratios adjusted for age and sex (aHR) were then fully adjusted (fully aHR) for age, sex, BMI, smoking, index of multiple deprivation quintile, and comorbidities

^8^change in hazard ratios for hypertension (from aHR to fully aHR) was investigated: diabetes and obesity were principally responsible for this reduction (HR 0.97 [0.92 ; 1.01] adjusted for age, sex, diabetes and obesity). Given the strong association between blood pressure and age, the authors examined the interaction between these variables; this revealed strong evidence of interaction (P <0.001), with hypertension associated with a higher risk up to the age of 70 years and a lower risk above the age of 70.

# Supplementary Table 4. Observational studies evaluating the impact of ACEIs/ARBs on the risk of a positive COVID-19 test (A) and on the course of the disease in infected patients (B)

## Impact of ACEIs/ARBs on the risk of a positive COVID-19 test

| **Paper** | **Country** | **Number of patients** | **Population** | **Outcome** | **Evaluated drug(s)** | **Reported effect** |
| --- | --- | --- | --- | --- | --- | --- |
| Amat-Santos – 2020  JACC  (Amat-Santos et al., 2020) | Spain | 102 | Patients with aortic stenosis successfully treated with transcatheter aortic valve replacement randomized to receive Ramipril | Positive COVID-19 test | ACEI | uHR 1.15 [0.35 ; 3.77] |
| Chodick – 2020  J Travel Med  (Chodick et al., 2020) | Israel | 14 520^1^ | All patients tested for COVID-19 | Positive COVID-19 test | ACEI | aOR 1.18 [0.87 ; 1.61] |
|  |  |  |  |  | ARB | aOR 1.29 [0.93 ; 1.79] |
|  |  |  |  |  | ACEI/ARB | aOR 1.19 [0.96 ; 1.47] |
| De Abajo – 2020  Lancet  (de Abajo et al., 2020) | Spain | 1139^2^ | All patients tested for COVID-19 | Positive COVID-19 test and requiring hospitalization | ACEI/ARB (versus other antihypertensive drugs) | aOR 0.94 [0.77 ; 1.15]  Restricted to hypertensive patients:  aOR 0.95 [0.75 ; 1.21] |
| Lee – 2020  Korean J Intern Med  (Lee et al., 2020) | Korea | 64 243^3^ | Patients with hypertension tested for COVID-19 | Positive COVID-19 test | ACEI/ARB | uOR 1.25 [1.13 ; 1.39]  aOR 1.22 [1.10 ; 1.36] |
| Mancia – 2020  NEJM  (Mancia et al., 2020) | Italy | 6272^4^ | All patients tested for COVID-19 | Positive COVID-19 test | ACEI | aOR 0.96 [0.87 ; 1.07] |
|  |  |  |  |  | ARB | aOR 0.95 [0.86 ; 1.05] |
| Mehta – 2020  JAMA Cardiol  (Mehta et al., 2020) | USA | 18 472^5^ | All patients tested for COVID-19 | Positive COVID-19 test | ACEI/ARB | PS-weighted OR 0.97 [0.81 ; 1.15] |
| Fosbøl – 2020  JAMA  (Fosbøl et al., 2020) | Denmark | 571^6^ | Patients with hypertension tested for COVID-19 | Positive COVID-19 test | ACEI/ARB | aHR 1.05 [0.80 ; 1.36] |
| Raisi-Estabragh – 2020  Front Cardiovasc Med  (Raisi-Estabragh et al., 2020) | United Kingdom | 7099 | Patients tested for COVID-19 (in a hospital setting) | Positive COVID-19 test | ACEI/ARB | uOR 1.01 [0.88 ; 1.17]  aOR 0.99 [0.83 ; 1.19] |
| Reynolds – 2020  NEJM  (Reynolds et al., 2020) | USA | 12 594^7^ | All patients tested for COVID-19 | Positive COVID-19 test | ACEI/ARB | Likelihood ratio −0.5 [−2.6 ; 3.6] |
| Savarese – 2020  Eur J Heart Fail  (Savarese et al., 2020) | Sweden | 1 387 746 | Patients with a diagnosis of heart failure, hypertension, kidney disease, diabetes or ischemic heart disease in the Swedish National Patient Registry | Incident hospitalization/death for COVID-19 | ACEI/ARB | ACEI or ARB:  uOR 0.79 [0.75 ; 0.83]  aOR 0.85 [0.81 ; 0.91]  ACEI:  uOR 1.00 [0.95 ; 1.05]  aOR 0.97 [0.92 ; 1.03]  ARB:  uOR 0.76 [0.72 ; 0.80]  aOR 0.88 [0.83 ; 0.84] |
| Seo – 2020  Korean J Intern Med  (Seo and Son, 2020) | Korea | 1644^8^ | Patients with hypertension tested for COVID-19 | Positive COVID-19 test | ACEI/ARB | ACEI or ARB exposure:  uOR 0.96 [0.83 ; 1.10]  aOR 0.98 [0.85 ; 1.41]  ACEI exposure:  uOR 0.88 [0.67 ; 1.14]  aOR 1.07 [0.81 ; 1.42]  ARB exposure:  uOR 0.97 [0.85 ; 1.11]  aOR 0.96 [0.84 ; 1.12] |
| Son – 2020  Hypertension  (Son et al., 2020) | South Korea | 950^9^ | Patients with hypertension tested for COVID-19 | Positive COVID-19 test | ACEI/ARB | aOR 1.16 [0.96 ; 1.41] |

uHR: unadjusted hazard ratio

aOR and aHR: adjusted odds ratio and adjusted hazard ratio respectively, PS: propensity score

ACEI: ACE inhibitor, ARB: angiotensin II receptor blocker

^1^amongst 14520 patients tested for COVID-19, 1317 were positive.

^2^patients were matched to 11390 controls

^3^patients were matched to 40 356 controls

^4^patients were matched to 30 759 beneficiaries of the Regional Health Service as controls

^5^amongst 18472 patients tested for COVID-19, 1735 were positive.

^6^patients were age- and sex-matched to 5710 controls with hypertension but not COVID-19

^7^amongst 12,594 patients tested for COVID-19, 5894 were positive

^8^patients were matched to 3288 controls

^9^patients were matched to 15 331 controls with hypertension but not COVID-19

## Impact of ACEIs/ARBs on the course of the disease in infected patients

| **Paper** | **Country** | **Number of patients** | **Population** | **Outcome** | **Evaluated drug(s)** | **Reported effect** | **Comment** |
| --- | --- | --- | --- | --- | --- | --- | --- |
| **Chronic exposure to RAAS-blockers** | | | | | | | |
| Conversano – 2020  Hypertension  (Andrea et al., 2020) | Italy | 191 (96 with hypertension) | Inpatients with COVID-19 | Death | ACEI/ARB | Overall:  uHR 1.8 [1.0 ; 3.3]  Hypertensive subjects:  uHR 0.5 [0.2 ; 1.2] | - |
| Covino – 2020  Intern Med J  (Covino et al., 2020) | Italy | 166 | Inpatients with hypertension and COVID-19 | Composite (Death/ICU admission) | ACEI/ARB | aOR 1.30 [1.58 ; 2.92] | - |
|  |  |  |  | Death |  | aOR 0.78 [0.29 ; 2.09] |  |
| Fosbøl – 2020  JAMA  (Fosbøl et al., 2020) | Denmark | 4480 | In- and outpatients with COVID-19 | Severity or ICU admission | ACEI/ARB | uHR 2.34 [1.97 ; 2.77]  aHR 1.15 [0.95;1.41] | - |
|  |  |  |  | Death or severe disease | ACEI/ARB | uHR 2.49 [2.15 ; 2.88]  aHR 1.04 [0.89 ; 1.23] |  |
|  |  |  |  | Death | ACEI/ARB | uHR 2.65 [2.18 ; 3.23]  aHR 0.83 [0.67 ; 1.03] |  |
| Felice – 2020  Am J Med  (Felice et al., 2020) | Italy | 133 | Patients with hypertension referred to emergency department and diagnosed with COVID-19 | ICU admission | ACEI/ARB | uOR 0.36 [0.17 ; 0.75]  aOR 0.25 [0.09 ; 0.66] | - |
|  |  |  |  | Death |  | uOR 0.41 [0.18 ; 0.92]  aOR 0.56 [0.17 ; 1.83] |  |
| Gao – 2020  Eur Heart J  (Gao et al., 2020) | China | 710 | Inpatients with COVID-19 and treated hypertension | Death | ACEI/ARB | uHR 0.60 [0.20 ; 1.76]  aOR 0.85 [0.28 ; 2.58]  PS-adjusted OR 0.93 [0.31 ; 2.84] | - |
| Giorgi Rossi – 2020  PLoS One  (Giorgi Rossi et al., 2020) | Italy | 425 | Patients with COVID-19 and coronary heart disease, hypertension or heart failure | Hospitalization | ACEI | aHR 1.12 [0.82 ; 1.54] | - |
|  |  |  |  |  | ARB | aHR 1.07 [0.78 ; 1.49] |  |
|  |  | 528 |  | Death | ACEI | aHR 0.8 [0.50 ; 1.3] |  |
|  |  |  |  |  | ARB | aHR 1.1 [0.7 ; 1.8] |  |
| Holt – 2020  J Hypertension  (Holt et al., 2020) | Denmark | 689 | Inpatients with COVID-19 | ICU admission or death | ACEI/ARB | aOR 0.80 [0.52 ; 1.22] | - |
| Iaccarino – 2020  Hypertension  (Iaccarino et al., 2020) | Italy | 1591 | Inpatients with COVID-19 | Death | ACEI | Exposure among dead vs survivors: 33.5% vs 20.3% (p=0.001)  aOR 1.474, p non-significant (95% CI not reported) | - |
|  |  |  |  |  | ARB | Exposure among dead vs survivors: 22.9% vs 18.8% (p non-significant, not reported) |  |
| Inciardi – 2020  Eur Heart J  (Inciardi et al., 2020) | Italy | 99 | Inpatients with cardiac disease and COVID-19 | Death | ACEI/ARB | Standardized mean difference -0.37 [-1.00 ; 0.26] | RAAS blockers were discontinued in 77% of patients during hospitalization |
| Jung – 2020  Clin Infect Dis  (Jung et al., 2020b) | South Korea | 5179 | In- and oupatients with COVID-19 (N=5179, of whom 1157 with hypertension) | Death | ACEI/ARB | Total population:  uOR 3.88 [2.48 ; 6.05]  aOR 0.88 [0.53 ; 1.44]  Hypertensive patients:  uOR 0.74 [0.43 ; 1.28]  aOR 0.71 [0.40 ; 1.26] | - |
| Jung – 2020  Eur Heart J Cardiovasc Pharmacother  (Jung et al., 2020a) | Multinational (38 countries) | 324 | Patients >70 years old with COVID-19 admitted to ICU | Death (in ICU) | ACEI | uOR 0.46 [0.26 ; 0.84]  PS-adjusted OR 0.32 [0.15 ; 0.67] | - |
|  |  |  |  |  | ARB | uOR 0.99 [0.62 ; 1.61]  aOR not reported |  |
| Lafaurie – 2020  Fundam Clin Pharmacol  (Lafaurie et al., 2020) | France | 111 | Inpatients with hypertension or cardiovascular disease and COVID-19 | Composite (ICU admission, mechanical ventilation, death) | ACEI/ARB | Total population:  ACEI or ARB:  uOR 1.43 [0.43 ; 3.19]  aOR 1.40 [0.66 ; 2.99]  ACEI:  uOR 0.84 [0.36 ; 1.94]  aOR 0.93 [0.44 ; 1.98]  ARB:  uOR 1.63 [0.75 ; 3.54]  aOR 1.54 [0.72 ; 3.27]  Hypertensive patients:  ACEI or ARB:  uOR 1.38 [0.57 ; 3.349]  aOR 1.95 [0.596 ; 6.40]  ACEI:  uOR 0.80 [0.34 ; 1.87]  aOR 0.89 [0.41 ; 1.96]  ARB:  uOR 1.58 [0.71 ; 3.53]  aOR 1.42 [0.64; 3.13] | - |
| Lee – 2020  Korean J Intern Med  (Lee et al., 2020) | Korea | 1609 | In- and outpatients with hypertension and COVID-19 | Death | ACEI/ARB | uOR 0.59 [0.43 ; 0.82]  aOR 0.81 [0.56 ; 1.17] | - |
| Li – 2020  J Allergy Clin Immunol  (Li et al., 2020b) | China | 548 | Inpatients with COVID-19 | Severity according to the American Thoracic Society | ACEI/ARB | Exposure among severe vs non-severe:  7.1% vs 8.2% (p=0.748) | - |
| Liaboeuf – 2020  Eur Heart J  (Liabeuf et al., 2020) | France | 268 | Inpatients with COVID-19 | ICU admission or death | ACEI/ARB | uOR: 2.01 [1.21;3.34]  aOR 1.73 [1.02 ; 2.93] | - |
| Matsuzawa – 2020  Hypertens Res  (Matsuzawa et al., 2020) | Japan | 151 | Inpatients with COVID-19 | Composite (death, ICU admission, extracorporeal membrane oxygenation, mechanical ventilation) | ACEI/ARB | uOR 0.43 [0.08 ; 2.09]  aOR 0.37 [0.05 ; 2.29] | - |
|  |  |  |  | In-hospital death |  | uOR 0.53 [0.06 ; 3.57]  aOR 0.36 [0.03 ; 3.53] |  |
| Mehta – 2020  JAMA Cardiol  (Mehta et al., 2020) | USA | 1735 | In- and outpatients with COVID-19 | Severity (hospitalization, ICU admission, mechanical ventilation) | ACEI/ARB | PS-matched OR for  Hospitalization: 1.93 [1.38 ; 2.71]  ICU admission: 1.64 [1.07 ; 2.51]  Mechanical ventilation: 1.32 [0.80 ; 2.18] | - |
| Mancia – 2020 NEJM  (Mancia et al., 2020) | Italy | 6272 | In- and outpatients with COVID-19 | Critical or fatal disease | ACEI | aOR 0.91 [0.69 ; 1.21] | - |
|  |  |  |  |  | ARB | aOR 0.83 [0.63 ; 1.10] |  |
| Negreira-Caamaño – 2020  High Blood Press Cardiovasc Prev  (Negreira-Caamaño et al., 2020) | Spain | 545 | Inpatients with hypertension and COVID-19 | Composite (death or mechanical ventilation) | ACEI/ARB | Percentage of the composite criteria occurrence in ACEI/ARB vs other antihypertensives treated patients:  31.6% vs 41.8% (p=0.024)  aOR 0.64 [0.44 ; 0.95] | - |
|  |  |  |  | Death |  | Percentage of the death in ACEI / ARB vs other antihypertensives treated patients:  30.4% vs 41.2% (p=0.016)  aOR 0.62 [0.42 ; 0.92] |  |
| Pan – 2020  Hypertension  (Pan et al., 2020) | China | 282 | Inpatients with COVID-19 and hypertension | Death | ACEI/ARB | Percentage of outcome in treated vs untreated:  7.3% vs 12.4%, p=0.495 | No adjusted analyses |
|  |  |  |  | ICU admission |  | Percentage of outcome in treated vs untreated:  9.8% vs 26.1%, p=0.037 |  |
| Palazzuoli – 2020  J Am Heart Assoc  (Palazzuoli et al., 2020) | Italy | 781 | Inpatients with COVID-19 | Death | ACEI/ARB | Percentage of the death in treated vs non-treated patients:  15.5% vs 14.9% (p=0.83)  ACEI vs no RAAS-blockers:  uOR 0.98 [0.60 ; 1.60]  aOR 0.55 [0.31 ; 0.98]  ARB vs no RAAS-blockers:  uOR 1.13 [0.07 ; 1.91]  aOR 0.59 [0.32 ; 1.07]  Hypertensive patients:  ACEI uOR 0.49 [0.28 ; 0.86]  ARB uOR 0.57 [0.32 ; 1.01]  (adjusted odd ratios not reported) | - |
| Reynolds – 2020  NEJM  (Reynolds et al., 2020) | USA | 5894 (2573 with hypertension) | In- and outpatients with COVID-19 | Severity (ICU, mechanical ventilation, death) | ACEI/ARB | Median difference [95%CI]  Total population: −0.1 [−3.7 ; 3.5]  Hypertensive patients: -0.5 [-4.3 ; -3.2] | - |
| Richardson – 2020  JAMA  (Richardson et al., 2020) | USA | 2411 patients with COVID19 and available outcome data, of whom 1366 with hypertension | Inpatients with COVID-19 and hypertension | Death | ACEI/ARB | Percentage of death in untreated,  ACE inhibitors and ARBs among  hypertensive subjects:  26.6%, 32.7%, and 30.6%  uOR 1.26 [0.98 ; 1.63] |  |
| Sardu – 2020  J Am Heart Assoc  (Sardu et al., 2020) | Italy | 62 | Inpatients with hypertension and COVID-1 | ICU admission | ACEI/ARB  (CCB: calcium chain blocker, used as comparator) | Percentage of event among ACEI, ARB and CCB treated patients:  16.7% vs 23.8% vs 17.6%  Univariate analysis ACEI vs CCB:  P=0.52 (multivariate analysis not reported)  Univariate analysis ARB vs CCB:  P=0.48 (multivariate analysis not reported) | - |
|  |  |  |  | Mechanical ventilation |  | Percentage of event among ACEI, ARB and CCB treated patients:  41.7% vs 42.9% vs 41.2%  Univariate analysis ACEI vs CCB:  P=0.58 (multivariate analysis not reported)  Univariate analysis ARB vs CCB:  P=0.59 (multivariate analysis not reported) |  |
|  |  |  |  | Death |  | Percentage of event among ACEI, ARB and CCB treated patients:  16.6% vs 14.3% vs 11.8%  Univariate analysis ACEI vs CCB:  P=0.06 (multivariate analysis not reported)  Univariate analysis ARB vs CCB:  P=0.57 (multivariate analysis not reported) |  |
| Savarese – 2020  Eur J Heart Fail  (Savarese et al., 2020) | Sweden | 7 146 | COVID-19 cases detected among the Swedish National Patient Registry | All-cause death |  | ACEI or ARB:  uHR 0.76 [0.71 ; 0.81]  aHR 0.89 [0.82 ; 0.96]  ACEI:  uHR 0.94 [0.87 ; 1.02]  aHR 1.00 [0.92 ; 1.09]  ARB:  uHR 0.75 [0.69 ; 0.81]  aHR 0.87 [0.80 ; 0.95]  Among hypertensive patients (ACEI or ARB):  uHR 0.71 [0.66 ; 0.76]  aHR 0.88 [0.81 ; 0.96] | - |
| Shah – 2020  J Hypertens  (Shah et al., 2020) | USA | 531 | African-American inpatients with COVID-19 | ICU admission | ACEI/ARB | uOR 1.68 [1.12 ; 2.54]  aOR 1.26 [0.74 ; 2.15] | - |
|  |  |  |  | Mechanical Ventilation |  | uOR 1.49 [0.96 ; 2.32]  aOR 1.24 [0.70 ; 2.20] |  |
|  |  |  |  | Death |  | uOR 1.29 [0.81 ; 2.02]  aOR 0.82 [0.45 ; 1.50] |  |
| Seo – 2020  Korean J Intern Med  (Seo and Son, 2020) | Korea | 152 (compared to 271 controls) | Inpatients with hypertension and severe COVID-19 leading to death | Death | ACEI/ARB | Exposure among COVID-19 cases vs non-COVID-19 cases:  31.6% vs 31.7%  ACEI/ARB:  uOR 1.01 [0.67 ; 1.54]  aOR 0.86 [0.55 ; 1.40]  ACEI:  uOR 0.88 [0.42 ; 1.87]  aOR 0.71 [0.32 ; 1.58]  ARB:  uOR 1.14 [0.75 ; 1.73]  aOR 1.02 [0.65 ; 1.62] | - |
| Son – 2020  Hypertension  (Son et al., 2020) | South Korea | 950 | In- and outpatients with hypertension and COVID-19 | ICU admission | ACEI/ARB | uOR 1.50 [0.42 ; 5.40]  aOR 1.52 [0.40 ; 5.70] | - |
|  |  |  |  | Death |  | uOR 1.36 [0.52 ; 3.53]  aOR 1.36 [0.51 ; 3.66] |  |
| Tedeschi – 2020  Clin Infect Dis  (Tedeschi et al., 2020) | Italy | 311 | Inpatients with hypertension and COVID-19 | Death | ACEI/ARB | aHR 0.97 [0.68 ;1.39] | - |
| Trifirò – 2020  Drug Safety  (ITA-COVID-19: RAAS inhibitor group et al., 2020) | Italy | 42926, including: 4663 and 4859 chronic users of ACE and ARB respectively, of which 1194 hypertensive patients included in the analysis of ARB versus non-use, and 1975 in the analysis of ACE inhibitors versus non-use | Inpatients with COVID-19 | Death | ACEI (vs non-use) | Overall:  uHR 2.24 [2.11 ; 2.37]  aHR 1.10 [1.03 ; 1.17]  Hypertensive patients:  aHR 1.12 [0.93 ; 1.31] | - |
|  |  |  |  |  | ARB (vs non-use) | Overall:  uHR 1.99 [1.87 ; 2.11]  aHR 1.12 [1.05 ; 1.20]  Hypertensive patients:  aHR 1.11 [0.95 ; 1.31] |  |
| Yahyavi – 2020  Intern Emerg Med  (Yahyavi et al., 2020) | Iran | 2553 | Inpatients with COVID-19 | Death | ACEI/ARB | uOR 1.3 [1.1 ; 1.7]  aOR 0.5 [0.4 ; 0.7] | - |
| **In-hospital exposure to RAAS-blockers or chronic treatment continued after hospital admission** | | | | | | | |
| Bravi – 2020  PLoS One  (Bravi et al., 2020) | Italy | 543 | In- and outpatients with COVID-19 and hypertension | Composite (admission to hospital, admission to ICU or death) | ACEI/ARB | 0.58 [0.34 ; 1.01]  Percentage of treated in “No  hospital admission” vs “Hospital  admission not ICU” vs “ICU or  death”:  Overall: 19.1% vs 35.9% vs 54.2%  Hypertensive subjects: 88.4% vs  78.7% vs 80.6% | treatment exposure recorded during the previous two years, but “whose medication was not discontinued during the follow-up” |
| Cannata – 2020  Eur Heart J Cardiovasc Pharmacother  (Cannata et al., 2020) | Italy | 397 | Inpatients with COVID-19 | Death | ACEI/ARB | Continued vs interruption/untreated:  uOR 0.54 [0.24 ; 1.25]  aOR 0.14 [0.03 ; 0.66] | - |
| Chaudhri – 2020  Kidney 360  (Chaudhri et al., 2020) | USA | 80 | Inpatients with COVID-19 and prior use of RAAS blockers | ICU admission | ACEI/ARB | Continued vs interruption:  aOR 0.25 [0.08 ; 0.81] | - |
|  |  |  |  | Death |  | Continued vs interruption:  aOR 0.31 [0.08 ; 1.26] |  |
| De Spiegeleer – 2020  J Am Med Dir Assoc  (De Spiegeleer et al., 2020) | Belgium | 154 | All patients with COVID-19 in 2 nursing homes | Composite (death or hospital length of stay > 7 days) | ACEI/ARB | Percentage of outcome in treated vs untreated:  20% vs 25%, p=0.495  uOR 0.79 [0.26 ; 1.95]  aOR 0.72 [0.10 ; 5.46] | - |
| Lam – 2020  J Infect Dis  (Lam et al., 2020) | USA | 614 | Inpatients with hypertension and COVID-19 | Death | ACEI/ARB | Chronic exposure vs non exposed:  Death:  17.31% vs 22.22%  Unadjusted p=0.127  Adjusted p=0.336  ICU admission:  6.10% vs 28.07%  Unadjusted p=0.923  Adjusted p=0.391  Continued vs interruption:  Death:  6.09% vs 28.07%  Unadjusted p=0.001  aOR 0.215 [0.101 ; 0.455]  ICU admission:  12.2% vs 26.3%  Unadjusted p=0.001  Adjusted p=0.001 | When stratified on development of hypotension or acute kidney injury:  Hypotension: continued vs interruption  Death: 26.92% vs 37.94%  Unadjusted p=0.063  aOR 0.22 [0.10 ; 0.46]  ICU admission: 30.8% vs 52.1%  Unadjusted p=0.06  aOR 0.35 [0.12 ; 1.03]  No hypotension: continued vs interruption:  Death: 2.17% vs 13.27%  Unadjusted p=0.001  aOR 0.17 [0.04 ; 0.73]  ICU admission: 8.7% vs 7.1%  Unadjusted p=0.67  aOR 1.06 [0.38 ; 2.98]  Acute kidney injury: continued vs interruption  Death: 16.12% vs 34.65%  Unadjusted p=0.050  aOR 0.42 [0.14 ; 1.24]  ICU admission: 22.6% vs 30.7%  Unadjusted p=0.38  aOR 0.47 [0.17 ; 1.31]  No acute kidney injury: continued vs interruption:  Death: 3.75% vs 18.57%  Unadjusted p=0.001  aOR 0.22 [0.07 ; 0.71]  ICU admission: 9.8% vs 20%  Unadjusted p<0.041  aOR 9.41 [0.18 ; 0.97] |
| Lahens – 2020  J Hypertens  (Lahens et al., 2020) | France | 347 | Inpatients with COVID-19 | Death or Severe disease (need for at least 9 l/min of oxygen (the threshold for high concentration mask in our unit), ICU admission, or death) | ACEI/ARB | Chronic exposure:  Death:  uOR 1.60 [0.82 ; 2.89]  aOR 0.62 [0.25 ; 1.48]  Severe disease:  uOR 0.84 [0.52 ; 1.33]  aOR 0.39 [0.20 ; 0.74]  Inhospital exposure:  Death:  uOR 0.51 [0.20 ; 1.11]  aOR 0.25 [0.09 ; 0.65]  Severe disease:  uOR 0.43 [0.24 ; 0.75]  aOR 0.23 [0.11 ; 0.45] | Our study did not find an association between chronic use of RAAS blockers and mortality in patients with COVID19, while the inverse association with disease severity might reflect a selection bias. In-hospital exposure generates a biased seemingly protective effect of treatment. |
| Li – 2020  JAMA Cardiol  (Li et al., 2020a) | China | 362 | Inpatients with COVID-19 and hypertension | Severe disease (5^th^ Chinese COVID-19 Guidelines) | ACEI/ARB | Percentage in users vs non-users among hypertensive subjects:  49.6% vs 47.0%, p=0.65 | - |
|  |  |  |  | Death |  | Percentage in users vs non-users among hypertensive subjects:  18.3% vs 22.7%, p=0.34 |  |
| Meng – 2020  Em Microb Infect  (Meng et al., 2020) | China | 42 | Inpatients with COVID-19 and treated hypertension | Severe disease (Chinese Guidelines) | ACEI/ARB | Percentage of severe disease in ACEIs or ARBs vs other:  23.5% vs 48% (p non-significant) | - |
| Xu – 2020  Front Med  (Xu et al., 2020) | China | 101 | Patients with hypertension and COVID-19 | ICU admission | ACEI/ARB | uOR 0.65 [0.25 ; 1.70]  aOR 0.68 [0.26 ; 1.81] | Exposure ”either recorded in the medical history or in the prescribed medication chart as standing order during hospitalization” |
|  |  |  |  | Mechanical ventilation |  | uOR 0.87 [0.31 ; 2.43]  aOR 0.92 [0.32 ; 2.63] |  |
|  |  |  |  | Death |  | uOR 0.73 [0.29 ; 1.82]  aOR 0.78 [0.32 ; 1.93] |  |
| Yang – 2020  Hypertension  (Yang et al., 2020) | China | 126 | Inpatients with hypertension and COVID-19 | Critical disease | ACEI/ARB | Percentage in treated vs untreated:  9.3% vs 22.9%, p=0.061 | - |
|  |  |  |  | Death |  | Percentage in treated vs untreated:  4.7% vs 13.3%, p=0283 |  |
| Zhang – 2020  Circ Res  (Zhang et al., 2020) | China | 1128 | Inpatients with hypertension and COVID-19 | Death | ACEI/ARB | ACEI/ARB vs non-use:  uHR 0.37 [0.17 ; 0.79]  PS-matched aHR 0.37 [0.15 ; 0.89]  ACEI/ARB vs other:  PS matched sample: 0.29 (0.12;0.69), p=0.005 | - |
| Zhou – 2020  Hypertension  (Zhou et al., 2020) | China | 3572 | Inpatients with COVID-19 and indication for ACE/ARB | Death | ACEI/ARB | Total population:  aHR 0.39 [0.26 ; 0.58]  Hypertensive patients:  aHR 0.32 [0.15 ; 0.66] | - |
| **Unclear exposure measurement** | | | | | | | |
| Bean – 2020  Eur J Heart Failure  (Bean et al., 2020) | UK | 1200 | Inpatients with COVID-19 | Composite (ICU or death) | ACEI/ARB | Chronic use:  uOR 0.83 [0.64 ; 1.07]  aOR 0.63 [0.47 ; 0.84]^5^  In-hospital use: similar results (not shown) | chronic use, but a sensitivity analysis using only in-hospital medications yielded the same results |
| Guo – 2020  JAMA Cardiol  (Guo et al., 2020) | China | 187 | Inpatients with COVID-19 | Death | ACEI/ARB | Percentage in users vs non-users:  36.8% vs 21.4% (p=0.13) |  |
| Feng – 2020  AJRCCM  (Feng et al., 2020) | China | 476 of whom 113 with hypertension | Inpatients with COVID-19 | Severity (Chinese classification, 5^th^ version) | ACEI/ARB | Among hypertensive patients, percentage of patients receiving an ACEIs or ARBs in moderate vs severe vs critical disease:  87.9% vs 6.1% vs 6.1% (p=0.004) | - |
| Huang – 2020  Ann Transl Med  (Huang et al., 2020) | China | 50 | Inpatients with hypertension and COVID-19 | Death | ACEI/ARB | Percentage in users vs non-users:  0% vs 10%, p=0.265 | - |
| Selçuk – 2020  Clin Exp Hypertens  (Selçuk et al., 2020) | Turkey | 113 | Inpatients with hypertension and COVID-19 | Death | ACEI/ARB | Exposure among dead vs survivors:  88.6% vs 55.1% (p<0.001)  uOR 6.30 [2.03 ; 19.58]  aOR 3.66 [1.11 ; 18.18] | - |

aOR and aHR: adjusted odds ratio and adjusted hazard ratio respectively, PS: propensity score. ACEI: ACE inhibitor, ARB: angiotensin II receptor blocker; ICU: intensive care unit

# Supplementary Table 5. Ongoing clinical trials (*last update: January 25^th^ 2021)*

| **Study coordination** | **Country**  **(Target N)** | **Intervention** | **Primary outcome** | **Status** | **Identifier** |
| --- | --- | --- | --- | --- | --- |
| **Prevention in patients not known to have COVID-19** | | | | | |
| National University of Ireland, Galway, Ireland  (CORONACION) | Ireland (2414) | Switch to an alternative blood pressure medication (specifically calcium chain blocker or thiazide/thiazide-like diuretic) | Number of COVID-19 positive participants who die, require intubation in ICU, or require hospitalization for non-invasive ventilation | Suspended (very low incidence of COVID-19 at Irish study site) | NCT04330300 |
| **Randomized studies on discontinuation or continuation of RAAS blockers in previously treated patients hospitalized for COVID-19** | | | | | |
| University of Pennsylvania  (REPLACECOVID)  (Cohen et al.) | Multinational, 20 hospitals (152) | Suspension or Maintenance of Angiotensin Receptor Blockers and Angiotensin-converting Enzyme Inhibitors | Global rank score in which each participant was ranked against all other participants across four hierarchies of clinical outcomes collected over the duration of the hospitalization (time to death, days on invasive mechanical ventilation or extracorporeal membrane oxygenation, days on renal replacement therapy or pressor/inotropic therapy, and a modified sequential Organ Failure Assessment (SOFA) score) | Published  Treatment effect [95% CI]  Global rank score 8.0 [-13 ; 29] (p=0.61) | NCT04338009 |
| D'Or Institute for Research and Education  (BRACE CORONA)  (Lopes et al., 2021) | Brazil (659) | Suspension or Maintenance of Angiotensin Receptor Blockers and Angiotensin-converting Enzyme Inhibitors | Median days alive and out of the hospital | Published  Discontinuation vs continuation group : 21.9 days [standard deviation 8 days] vs 22.9 days [standard deviation 7.1 days]  Mean ratio 0.95 [0.90 ; 1.01]  Death: OR 0.97 [0.38 ; 2.52] | NCT04364893 |
| Medical University Innsbruck  (ACEI-COVID) | Austria and Germany (208) | Suspension or Maintenance of Angiotensin-converting Enzyme Inhibitors | Composite (maximum SOFA and death at 30 days) | Recruiting | NCT04353596 |
| McGill University Health Centre/Research Institute of the McGill University Health Centre  (RAASCOVID) | Canada (40) | Suspension or Maintenance of Angiotensin-converting Enzyme Inhibitors | Global rank score assessed from baseline to day 7 | Not yet recruiting | NCT04508985 |
| University Hospital, Gentofte, Copenhagen  (RASCOVID-19) | Denmark (215) | Suspension or Maintenance of Angiotensin Receptor Blockers and Angiotensin-converting Enzyme Inhibitors | Days alive and out of hospital within 14 days after recruitment | Recruiting | NCT04351581 |
| Assistance Publique - Hôpitaux de Paris  (ACORES-2) | France (554) | Suspension or Maintenance of Angiotensin Receptor Blockers and Angiotensin-converting Enzyme Inhibitors | Time to clinical improvement from day 0 to day 28 (improvement of two points on a seven-category ordinal scale, or live discharge from the hospital, whichever comes first) | Recruiting | NCT04329195 |
| University of Sao Paulo  (SWITCH-COVID) | Brazil (240) | Suspension or Maintenance of Angiotensin Receptor Blockers and Angiotensin-converting Enzyme Inhibitors | Need for ICU or mortality at 30 days | Recruiting | NCT04493359 |
| **Randomized studies on the effect of RAAS-blockers versus placebo in patients infected with COVID-19*** | | | | | |
| Hospital Regional de Alta Especialidad de Zumpango  (STAR-COVID) | Mexico (60) | Effectiveness and Safety of Telmisartan in Acute Respiratory Failure Due to COVID-19 | Composite (death and occurrence of mechanical ventilation at 30 days) | Recruiting | NCT04510662 |
| University of Minnesota | USA (200) | Effectiveness of Losartan 50 mg daily for 7 days (versus placebo, blinded) for patients with COVID-19 requiring hospitalization | Difference in Estimated (PEEP adjusted) P/F Ratio at 7 days | Recruiting | NCT04312009 |
| University of Minnesota | USA (580) | Effectiveness of Losartan 25 mg daily (versus placebo, blinded) for outpatients with COVID-19 | Hospital admission within 15 days of randomization | Active, not recruiting | NCT04311177 |
| University of Kansas Medical Center | USA (50) | Safety of Losartan in patients with respiratory failure due to COVID-19 | Number of participants with treatment-related adverse events as assessed by protocol definition of adverse effects | Completed | NCT04335123 |
| Bassett Healthcare  (COVIDMED group 3) | USA (4000) | Comparison between Lopinavir/ritonavir – Losartan (25 mg daily for 5-14 days) and Placebo for COVID-19 hospitalized patients | National Institute of Allergy and Infectious Diseases COVID-19 Ordinal Severity Scale at 60 days | Recruiting | NCT04328012 |
| Sharp HealthCare | USA (200) | Effectiveness of Losartan 12.5mg bid for up to 10 days (versus no losartan, open label) | Transfer into ICU for mechanical ventilation due to respiratory failure (time frame 45 days) | Recruiting | NCT04340557 |
| University of Hawaii | USA (40) | Telmisartan (40 mg daily for 21 days) in COVID-19 outpatients | Maximum clinical severity of disease based on a modified World Health Organization COVID-19 7-point ordinal scale on 21 days period | Recruiting | NCT04360551 |
| Laboratorio Elea Phoenix S.A. | Argentina (400) | Effectiveness of Telmisartan (80mg twice daily) in hospitalized COVID-19 patients | Serum C reactive protein levels at day 5 and 8  Interim analysis from 68 patients (target 400 patients) in preprint (Duarte et al MedRXiv 2020) | Recruiting | NCT04355936 |
| The George Institute  (CLARITY) | Australia (605) | Effectiveness of ARB in COVID-19 inpatients and outpatients with additional criteria | 7-Point National Institute of Health Clinical Health Score within 28 days from randomization | Recruiting | NCT04394117 |
| Radboud University  (PRAETORIAN COVID) | Netherlands (651) | Effectiveness of Valsartan (up to 160 mg bid for up to 14 days, versus placebo, blinded) in hospitalized COVID-19 patients | First occurrence of intensive care unit admission, mechanical ventilation or death within 14 days | Recruiting | NCT04335786 |
| University Hospital, Strasbourg  (COVID-Aging) | France (1600) | Comparison between several treatments including Telmisartan (40 mg bid for 14 days, open label) among hospitalized COVID-19 elderly patients | Two-week survival rate | Recruiting | NCT04359953 |
| University Hospital, Bordeaux  (COVERAGE) | France (338) | Comparison between several treatments including Telmisartan (20 mg daily for 10 days) among COVID-19 elderly outpatients | Composite of hospitalization or death at day 14 | Recruiting | NCT04356495 |
| Hospital Universitario Dr. Jose E. Gonzalez | Mexico (20) | Effectiveness of Chloroquine ± Losartan (25mg bid for 10 days) in hospitalized COVID-19 patients | All-cause mortality up to 28 days after randomization | Recruiting | NCT04428268 |
| Instituto do Cancer do Estado de São Paulo  (TITAN) | Brazil (176) | Effectiveness of Ivermectin and Losartan (50mg daily for 15 days) vs placebo (double blind) in cancer patients diagnosed COVID-19 | Incidence of Severe Acute Respiratory Syndrome (defined SpO2 < 93%) at 28 days | Recruiting | NCT04447235 |
| Medical University of Vienna  (ACOVACT Sub-study B | Austria (500) | Comparison between several treatments including Candesartan (4 mg daily titration to normotension) among COVID-19 in and outpatients | Time to clinical improvement which is defined as time from randomization to an (sustained) improvement of at least one category on two consecutive days compared to the status at randomization measured on a seven-category ordinal scale (proposed by WHO). | Recruiting | NCT04351724 |
| University of California, San Diego  (RAMIC) | USA (560) | Evaluate the efficacy of Ramipril to prevent ICU admission, need for mechanical ventilation or death in persons hospitalized with COVID-19 | Composite of mortality or need for ICU admission or ventilator use (at 14 days) | Enrolling on invitation | NCT04366050 |
| Kafrelsheikh University | Egypt (360) | Efficacy of Aerosol Combination Therapy of 13 Cis Retinoic Acid and Captopril (nebulization 25mg daily, open label) for patients admitted to ICU for Covid-19 | Lung injury score at day 7 | Not yet recruiting | NCT04578236 |
| Tanta University | Egypt (60) | Efficacy of ACEI (Captopril or Enalapril) vs chloroquin (open label) on COVID-19 outpatients | Number of patients with virological cure at 6 months | Not yet recruiting | NCT04345406 |
| London School of Hygiene and Tropical Medicine  (CRASH-19) | Multinational (10 000) | Comparison between several treatments including Losartan 100 mg daily among hospitalized COVID-19 elderly patients | Death up to 28 days after randomization | Withdrawn (grant not obtained) | NCT04343001 |
| Inserm-ANRS  (INTENSE-COV) | Ivory Coast (294) | Comparison between several treatments including Telmisartan 40mg daily for 10 days among hospitalized COVID-19 elderly patients | Proportion of patients with undetectable nasopharyngeal swab SARS-CoV-2 PCR and C-reactive protein (CRP) < 27 mg/L at Day 11 | Recruiting | NCT04466241 |
| Assistance Publique - Hôpitaux de Paris  (CAPTOCOVID) | France (230) | Effectiveness of nebulized 25 mg Captopril in hospitalized COVID-19 patients (open label) | ventilation-free survival at 14 days | Not Yet Recruiting | NCT04355429 |

*in most cases patients with chronic use of ACEIs/ARBs are excluded from these trials of RAAS blocker initiation

**REFERENCES FOR SUPPLEMENTARY MATERIAL**

Agata, J., Ura, N., Yoshida, H., Shinshi, Y., Sasaki, H., Hyakkoku, M., et al. (2006). Olmesartan Is an Angiotensin II Receptor Blocker with an Inhibitory Effect on Angiotensin-Converting Enzyme. *Hypertens Res* 29, 865–874. doi:10.1291/hypres.29.865.

Albitar, O., Ballouze, R., Ooi, J. P., and Sheikh Ghadzi, S. M. (2020). Risk factors for mortality among COVID-19 patients. *Diabetes Research and Clinical Practice* 166, 108293. doi:10.1016/j.diabres.2020.108293.

Amat-Santos, I. J., Santos-Martinez, S., López-Otero, D., Nombela-Franco, L., Gutiérrez-Ibanes, E., Del Valle, R., et al. (2020). Ramipril in High-Risk Patients With COVID-19. *Journal of the American College of Cardiology* 76, 268–276. doi:10.1016/j.jacc.2020.05.040.

Andrea, C., Francesco, M., Antonio, N., Evgeny, F., Marzia, S., Fabio, C., et al. (2020). Renin-Angiotensin-Aldosterone System Inhibitors and Outcome in Patients With SARS-CoV-2 Pneumonia: A Case Series Study. *Hypertension* 76. doi:10.1161/HYPERTENSIONAHA.120.15312.

Anguiano, L., Riera, M., Pascual, J., Valdivielso, J. M., Barrios, C., Betriu, A., et al. (2015). Circulating angiotensin-converting enzyme 2 activity in patients with chronic kidney disease without previous history of cardiovascular disease. *Nephrol. Dial. Transplant.* 30, 1176–1185. doi:10.1093/ndt/gfv025.

Bean, D. M., Kraljevic, Z., Searle, T., Bendayan, R., Kevin, O., Pickles, A., et al. (2020). Angiotensin‐converting enzyme inhibitors and angiotensin II receptor blockers are not associated with severe COVID‐19 infection in a multi‐site UK acute hospital trust. *Eur J Heart Fail* 22, 967–974. doi:10.1002/ejhf.1924.

Berenguer, J., Ryan, P., Rodríguez-Baño, J., Jarrín, I., Carratalà, J., Pachón, J., et al. (2020). Characteristics and predictors of death among 4035 consecutively hospitalized patients with COVID-19 in Spain. *Clinical Microbiology and Infection*, S1198743X20304316. doi:10.1016/j.cmi.2020.07.024.

Boulle, A., Davies, M.-A., Hussey, H., Ismail, M., Morden, E., Vundle, Z., et al. (2020). Risk factors for COVID-19 death in a population cohort study from the Western Cape Province, South Africa. *Clin Infect Dis*. doi:10.1093/cid/ciaa1198.

Bravi, F., Flacco, M. E., Carradori, T., Volta, C. A., Cosenza, G., De Togni, A., et al. (2020). Predictors of severe or lethal COVID-19, including Angiotensin Converting Enzyme inhibitors and Angiotensin II Receptor Blockers, in a sample of infected Italian citizens. *PLoS ONE* 15, e0235248. doi:10.1371/journal.pone.0235248.

Burchill, L. J., Velkoska, E., Dean, R. G., Griggs, K., Patel, S. K., and Burrell, L. M. (2012). Combination renin–angiotensin system blockade and angiotensin-converting enzyme 2 in experimental myocardial infarction: implications for future therapeutic directions. *Clinical Science* 123, 649–658. doi:10.1042/CS20120162.

Burrell, L. M., Burchill, L., Dean, R. G., Griggs, K., Patel, S. K., and Velkoska, E. (2012). Chronic kidney disease: cardiac and renal angiotensin-converting enzyme (ACE) 2 expression in rats after subtotal nephrectomy and the effect of ACE inhibition: Angiotensin-converting enzyme 2 and chronic kidney disease. *Experimental Physiology* 97, 477–485. doi:10.1113/expphysiol.2011.063156.

Burrell, L. M., Risvanis, J., Kubota, E., Dean, R. G., MacDonald, P. S., Lu, S., et al. (2005). Myocardial infarction increases ACE2 expression in rat and humans. *European Heart Journal* 26, 369–375. doi:10.1093/eurheartj/ehi114.

Cannata, F., Chiarito, M., Reimers, B., Azzolini, E., Ferrante, G., My, I., et al. (2020). Continuation versus discontinuation of ACE inhibitors or angiotensin II receptor blockers in COVID-19: effects on blood pressure control and mortality. *European Heart Journal - Cardiovascular Pharmacotherapy*, pvaa056. doi:10.1093/ehjcvp/pvaa056.

Cariou, B., Hadjadj, S., Wargny, M., Pichelin, M., Al-Salameh, A., Allix, I., et al. (2020). Phenotypic characteristics and prognosis of inpatients with COVID-19 and diabetes: the CORONADO study. *Diabetologia* 63, 1500–1515. doi:10.1007/s00125-020-05180-x.

Cen, Y., Chen, X., Shen, Y., Zhang, X.-H., Lei, Y., Xu, C., et al. (2020). Risk factors for disease progression in patients with mild to moderate coronavirus disease 2019—a multi-centre observational study. *Clinical Microbiology and Infection* 26, 1242–1247. doi:10.1016/j.cmi.2020.05.041.

Chaudhri, I., Koraishy, F. M., Bolotova, O., Yoo, J., Marcos, L. A., Taub, E., et al. (2020). Outcomes Associated with the Use of Renin-Angiotensin-Aldosterone System Blockade in Hospitalized Patients with SARS-CoV-2 Infection. *Kidney360* 1, 801–809. doi:10.34067/KID.0003792020.

Chen, J., Bai, H., Liu, J., Chen, G., Liao, Q., Yang, J., et al. (2020a). Distinct Clinical Characteristics and Risk Factors for Mortality in Female Inpatients With Coronavirus Disease 2019 (COVID-19): A Sex-stratified, Large-scale Cohort Study in Wuhan, China. *Clinical Infectious Diseases*. doi:10.1093/cid/ciaa920.

Chen, L., Yu, J., He, W., Chen, L., Yuan, G., Dong, F., et al. (2020b). Risk factors for death in 1859 subjects with COVID-19. *Leukemia* 34, 2173–2183. doi:10.1038/s41375-020-0911-0.

Chirinos, J. A., Cohen, J. B., Zhao, L., Hanff, T., Sweitzer, N., Fang, J., et al. (2020). Clinical and Proteomic Correlates of Plasma ACE2 (Angiotensin-Converting Enzyme 2) in Human Heart Failure. *Hypertension* 76, 1526–1536. doi:10.1161/HYPERTENSIONAHA.120.15829.

Chodick, G., Nutman, A., Yiekutiel, N., and Shalev, V. (2020). Angiotensin-converting enzyme inhibitors and angiotensin-receptor blockers are not associated with increased risk of SARS-CoV-2 infection. *Journal of Travel Medicine* 27. doi:10.1093/jtm/taaa069.

Cohen, J. B., Hanff, T. C., William, P., Sweitzer, N., Rosado-Santander, N. R., Medina, C., et al. Continuation versus discontinuation of renin–angiotensin system inhibitors in patients admitted to hospital with COVID-19: a prospective, randomised, open-label trial. *The Lancet Respiratory Medicine*. doi:10.1016/S2213-2600(20)30558-0.

Covino, M., De Matteis, G., Santoro, M., Sabia, L., Simeoni, B., Candelli, M., et al. (2020). Clinical characteristics and prognostic factors in COVID‐19 patients aged ≥80 years. *Geriatr. Gerontol. Int.* 20, 704–708. doi:10.1111/ggi.13960.

Cunningham, J. W., Vaduganathan, M., Claggett, B. L., Jering, K. S., Bhatt, A. S., Rosenthal, N., et al. (2020). Clinical Outcomes in Young US Adults Hospitalized With COVID-19. *JAMA Internal Medicine*. doi:10.1001/jamainternmed.2020.5313.

de Abajo, F. J., Rodríguez-Martín, S., Lerma, V., Mejía-Abril, G., Aguilar, M., García-Luque, A., et al. (2020). Use of renin–angiotensin–aldosterone system inhibitors and risk of COVID-19 requiring admission to hospital: a case-population study. *The Lancet* 395, 1705–1714. doi:10.1016/S0140-6736(20)31030-8.

De Spiegeleer, A., Bronselaer, A., Teo, J. T., Byttebier, G., De Tré, G., Belmans, L., et al. (2020). The Effects of ARBs, ACEis, and Statins on Clinical Outcomes of COVID-19 Infection Among Nursing Home Residents. *Journal of the American Medical Directors Association* 21, 909-914.e2. doi:10.1016/j.jamda.2020.06.018.

Epelman, S., Tang, W. H. W., Chen, S. Y., Van Lente, F., Francis, G. S., and Sen, S. (2008). Detection of Soluble Angiotensin-Converting Enzyme 2 in Heart Failure. *Journal of the American College of Cardiology* 52, 750–754. doi:10.1016/j.jacc.2008.02.088.

Felice, C., Nardin, C., Di Tanna, G. L., Grossi, U., Bernardi, E., Scaldaferri, L., et al. (2020). Use of RAAS Inhibitors and Risk of Clinical Deterioration in COVID-19: Results From an Italian Cohort of 133 Hypertensives. *American Journal of Hypertension*, hpaa096. doi:10.1093/ajh/hpaa096.

Feng, Y., Ling, Y., Bai, T., Xie, Y., Huang, J., Li, J., et al. (2020). COVID-19 with Different Severities: A Multicenter Study of Clinical Features. *Am J Respir Crit Care Med* 201, 1380–1388. doi:10.1164/rccm.202002-0445OC.

Ferrario, C. M., Jessup, J., Chappell, M. C., Averill, D. B., Brosnihan, K. B., Tallant, E. A., et al. (2005a). Effect of Angiotensin-Converting Enzyme Inhibition and Angiotensin II Receptor Blockers on Cardiac Angiotensin-Converting Enzyme 2. *Circulation* 111, 2605–2610. doi:10.1161/CIRCULATIONAHA.104.510461.

Ferrario, C. M., Jessup, J., Gallagher, P. E., Averill, D. B., Brosnihan, K. B., Ann Tallant, E., et al. (2005b). Effects of renin-angiotensin system blockade on renal angiotensin-(1-7) forming enzymes and receptors. *Kidney International* 68, 2189–2196. doi:10.1111/j.1523-1755.2005.00675.x.

Fosbøl, E. L., Butt, J. H., Østergaard, L., Andersson, C., Selmer, C., Kragholm, K., et al. (2020). Association of Angiotensin-Converting Enzyme Inhibitor or Angiotensin Receptor Blocker Use With COVID-19 Diagnosis and Mortality. *JAMA* 324, 168. doi:10.1001/jama.2020.11301.

Fried, M. W., Crawford, J. M., Mospan, A. R., Watkins, S. E., Munoz Hernandez, B., Zink, R. C., et al. (2020). Patient Characteristics and Outcomes of 11,721 Patients with COVID19 Hospitalized Across the United States. *Clin Infect Dis*. doi:10.1093/cid/ciaa1268.

Furuhashi, M., Moniwa, N., Mita, T., Fuseya, T., Ishimura, S., Ohno, K., et al. (2015). Urinary Angiotensin-Converting Enzyme 2 in Hypertensive Patients May Be Increased by Olmesartan, an Angiotensin II Receptor Blocker. *American Journal of Hypertension* 28, 15–21. doi:10.1093/ajh/hpu086.

Gao, C., Cai, Y., Zhang, K., Zhou, L., Zhang, Y., Zhang, X., et al. (2020). Association of hypertension and antihypertensive treatment with COVID-19 mortality: a retrospective observational study. *European Heart Journal* 41, 2058–2066. doi:10.1093/eurheartj/ehaa433.

Giorgi Rossi, P., Marino, M., Formisano, D., Venturelli, F., Vicentini, M., and Grilli, R. (2020). Characteristics and outcomes of a cohort of COVID-19 patients in the Province of Reggio Emilia, Italy. *PLoS One* 15, e0238281. doi:10.1371/journal.pone.0238281.

Gottlieb, M., Sansom, S., Frankenberger, C., Ward, E., and Hota, B. (2020). Clinical Course and Factors Associated With Hospitalization and Critical Illness Among COVID-19 Patients in Chicago, Illinois. *Acad Emerg Med*. doi:10.1111/acem.14104.

Guo, T., Fan, Y., Chen, M., Wu, X., Zhang, L., He, T., et al. (2020). Cardiovascular Implications of Fatal Outcomes of Patients With Coronavirus Disease 2019 (COVID-19). *JAMA Cardiol* 5, 811. doi:10.1001/jamacardio.2020.1017.

Gupta, S., Hayek, S. S., Wang, W., Chan, L., Mathews, K. S., Melamed, M. L., et al. (2020). Factors Associated With Death in Critically Ill Patients With Coronavirus Disease 2019 in the US. *JAMA Intern Med*. doi:10.1001/jamainternmed.2020.3596.

Hamming, I., Van Goor, H., Turner, A. J., Rushworth, C. A., Michaud, A. A., Corvol, P., et al. (2008). Differential regulation of renal angiotensin-converting enzyme (ACE) and ACE2 during ACE inhibition and dietary sodium restriction in healthy rats: Renal ACE and ACE2 during ACE inhibition and low salt. *Experimental Physiology* 93, 631–638. doi:10.1113/expphysiol.2007.041855.

Han, S.-X., He, G.-M., Wang, T., Chen, L., Ning, Y.-Y., Luo, F., et al. (2010). Losartan attenuates chronic cigarette smoke exposure-induced pulmonary arterial hypertension in rats: Possible involvement of angiotensin-converting enzyme-2. *Toxicology and Applied Pharmacology* 245, 100–107. doi:10.1016/j.taap.2010.02.009.

Hernández-Galdamez, D. R., González-Block, M. Á., Romo-Dueñas, D. K., Lima-Morales, R., Hernández-Vicente, I. A., Lumbreras-Guzmán, M., et al. (2020). Increased Risk of Hospitalization and Death in Patients with COVID-19 and Pre-existing Noncommunicable Diseases and Modifiable Risk Factors in Mexico. *Archives of Medical Research*, S0188440920307220. doi:10.1016/j.arcmed.2020.07.003.

Holt, A., Mizrak, I., Lamberts, M., and Lav Madsen, P. (2020). Influence of inhibitors of the renin–angiotensin system on risk of acute respiratory distress syndrome in Danish hospitalized COVID-19 patients. *Journal of Hypertension* Publish Ahead of Print. doi:10.1097/HJH.0000000000002515.

Huang, Z., Cao, J., Yao, Y., Jin, X., Luo, Z., Xue, Y., et al. (2020). The effect of RAS blockers on the clinical characteristics of COVID-19 patients with hypertension. *Ann Transl Med* 8, 430–430. doi:10.21037/atm.2020.03.229.

Iaccarino, G., Grassi, G., Borghi, C., Ferri, C., Salvetti, M., Volpe, M., et al. (2020). Age and Multimorbidity Predict Death Among COVID-19 Patients: Results of the SARS-RAS Study of the Italian Society of Hypertension. *Hypertension* 76, 366–372. doi:10.1161/HYPERTENSIONAHA.120.15324.

Igase, M., Strawn, W. B., Gallagher, P. E., Geary, R. L., and Ferrario, C. M. (2005). Angiotensin II AT _1_ receptors regulate ACE2 and angiotensin-(1–7) expression in the aorta of spontaneously hypertensive rats. *American Journal of Physiology-Heart and Circulatory Physiology* 289, H1013–H1019. doi:10.1152/ajpheart.00068.2005.

Inciardi, R. M., Adamo, M., Lupi, L., Cani, D. S., Di Pasquale, M., Tomasoni, D., et al. (2020). Characteristics and outcomes of patients hospitalized for COVID-19 and cardiac disease in Northern Italy. *Eur Heart J* 41, 1821–1829. doi:10.1093/eurheartj/ehaa388.

Ioannou, G. N., Locke, E., Green, P., Berry, K., O’Hare, A. M., Shah, J. A., et al. (2020). Risk Factors for Hospitalization, Mechanical Ventilation, or Death Among 10 131 US Veterans With SARS-CoV-2 Infection. *JAMA Netw Open* 3, e2022310. doi:10.1001/jamanetworkopen.2020.22310.

Ishiyama, Y., Gallagher, P. E., Averill, D. B., Tallant, E. A., Brosnihan, K. B., and Ferrario, C. M. (2004). Upregulation of Angiotensin-Converting Enzyme 2 After Myocardial Infarction by Blockade of Angiotensin II Receptors. *Hypertension* 43, 970–976. doi:10.1161/01.HYP.0000124667.34652.1a.

ITA-COVID-19: RAAS inhibitor group, Trifirò, G., Massari, M., Da Cas, R., Menniti Ippolito, F., Sultana, J., et al. (2020). Renin–Angiotensin–Aldosterone System Inhibitors and Risk of Death in Patients Hospitalised with COVID-19: A Retrospective Italian Cohort Study of 43,000 Patients. *Drug Saf*. doi:10.1007/s40264-020-00994-5.

Jessup, J. A., Gallagher, P. E., Averill, D. B., Brosnihan, K. B., Tallant, E. A., Chappell, M. C., et al. (2006). Effect of angiotensin II blockade on a new congenic model of hypertension derived from transgenic Ren-2 rats. *American Journal of Physiology-Heart and Circulatory Physiology* 291, H2166–H2172. doi:10.1152/ajpheart.00061.2006.

Jiang, X., Eales, J. M., Scannali, D., Nazgiewicz, A., Prestes, P., Maier, M., et al. (2020). Hypertension and renin-angiotensin system blockers are not associated with expression of angiotensin-converting enzyme 2 (ACE2) in the kidney. *European Heart Journal* 41, 4580–4588. doi:10.1093/eurheartj/ehaa794.

Jung, C., Bruno, R. R., Wernly, B., Joannidis, M., Oeyen, S., Zafeiridis, T., et al. (2020a). Inhibitors of the renin–angiotensin–aldosterone system and COVID-19 in critically ill elderly patients. *European Heart Journal - Cardiovascular Pharmacotherapy*, pvaa083. doi:10.1093/ehjcvp/pvaa083.

Jung, S.-Y., Choi, J. C., You, S.-H., and Kim, W.-Y. (2020b). Association of Renin-angiotensin-aldosterone System Inhibitors With Coronavirus Disease 2019 (COVID-19)- Related Outcomes in Korea: A Nationwide Population-based Cohort Study. *Clinical Infectious Diseases*. doi:10.1093/cid/ciaa624.

Karram, T., Abbasi, A., Keidar, S., Golomb, E., Hochberg, I., Winaver, J., et al. (2005). Effects of spironolactone and eprosartan on cardiac remodeling and angiotensin-converting enzyme isoforms in rats with experimental heart failure. *American Journal of Physiology-Heart and Circulatory Physiology* 289, H1351–H1358. doi:10.1152/ajpheart.01186.2004.

Kim, L., Garg, S., O’Halloran, A., Whitaker, M., Pham, H., Anderson, E. J., et al. (2020). Risk Factors for Intensive Care Unit Admission and In-hospital Mortality among Hospitalized Adults Identified through the U.S. Coronavirus Disease 2019 (COVID-19)-Associated Hospitalization Surveillance Network (COVID-NET). *Clinical Infectious Diseases*. doi:10.1093/cid/ciaa1012.

Lafaurie, M., Martin-Blondel, G., Delobel, P., and Charpentier, S. (2020). Outcome of patients hospitalized for COVID‐19 and exposure to angiotensin‐converting enzyme inhibitors and angiotensin‐receptor blockers in France: results of the ACE‐CoV study. *Clinical Pharmacology*, 10.

Lahens, A., Mullaert, J., Gressens, S., Gault, N., Flamant, M., Deconinck, L., et al. (2020). Association between renin–angiotensin–aldosterone system blockers and outcome in coronavirus disease 2019: analysing in-hospital exposure generates a biased seemingly protective effect of treatment. *Journal of Hypertension* Publish Ahead of Print. doi:10.1097/HJH.0000000000002658.

Lam, K. W., Chow, K. W., Vo, J., Hou, W., Li, H., Richman, P. S., et al. (2020). Continued In-Hospital Angiotensin-Converting Enzyme Inhibitor and Angiotensin II Receptor Blocker Use in Hypertensive COVID-19 Patients Is Associated With Positive Clinical Outcome. *The Journal of Infectious Diseases* 222, 1256–1264. doi:10.1093/infdis/jiaa447.

Lee, J. Y., Kim, H. A., Huh, K., Hyun, M., Rhee, J.-Y., Jang, S., et al. (2020). Risk Factors for Mortality and Respiratory Support in Elderly Patients Hospitalized with COVID-19 in Korea. *J Korean Med Sci* 35, e223. doi:10.3346/jkms.2020.35.e223.

Lely, A., Hamming, I., van Goor, H., and Navis, G. (2004). Renal ACE2 expression in human kidney disease. *J. Pathol.* 204, 587–593. doi:10.1002/path.1670.

Lezama-Martinez, D., Flores-Monroy, J., Fonseca-Coronado, S., Hernandez-Campos, M. E., Valencia-Hernandez, I., and Martinez-Aguilar, L. (2018). Combined Antihypertensive Therapies That Increase Expression of Cardioprotective Biomarkers Associated With the Renin–Angiotensin and Kallikrein–Kinin Systems: *Journal of Cardiovascular Pharmacology* 72, 291–295. doi:10.1097/FJC.0000000000000629.

Li, J., Wang, X., Chen, J., Zhang, H., and Deng, A. (2020a). Association of Renin-Angiotensin System Inhibitors With Severity or Risk of Death in Patients With Hypertension Hospitalized for Coronavirus Disease 2019 (COVID-19) Infection in Wuhan, China. *JAMA Cardiol* 5, 825. doi:10.1001/jamacardio.2020.1624.

Li, X., Xu, S., Yu, M., Wang, K., Tao, Y., Zhou, Y., et al. (2020b). Risk factors for severity and mortality in adult COVID-19 inpatients in Wuhan. *J Allergy Clin Immunol* 146, 110–118. doi:10.1016/j.jaci.2020.04.006.

Li, Y., Zeng, Z., Li, Y., Huang, W., Zhou, M., Zhang, X., et al. (2015). Angiotensin-Converting Enzyme Inhibition Attenuates Lipopolysaccharide-Induced Lung Injury by Regulating the Balance Between Angiotensin-Converting Enzyme and Angiotensin-Converting Enzyme 2 and Inhibiting Mitogen-Activated Protein Kinase Activation: *Shock* 43, 395–404. doi:10.1097/SHK.0000000000000302.

Liabeuf, S., Moragny, J., Bennis, Y., Batteux, B., Brochot, E., Schmit, J. L., et al. (2020). Association between renin–angiotensin system inhibitors and COVID-19 complications. *European Heart Journal - Cardiovascular Pharmacotherapy*, pvaa062. doi:10.1093/ehjcvp/pvaa062.

Liang, Y., Deng, H., Bi, S., Cui, Z., A, L., Zheng, D., et al. (2015). Urinary Angiotensin Converting Enzyme 2 Increases in Patients With Type 2 Diabetic Mellitus. *Kidney Blood Press Res* 40, 101–110. doi:10.1159/000368486.

Liu, D., Cui, P., Zeng, S., Wang, S., Feng, X., Xu, S., et al. (2020). Risk factors for developing into critical COVID-19 patients in Wuhan, China: A multicenter, retrospective, cohort study. *EClinicalMedicine* 25, 100471. doi:10.1016/j.eclinm.2020.100471.

Lopes, R. D., Macedo, A. V. S., de Barros E Silva, P. G. M., Moll-Bernardes, R. J., dos Santos, T. M., Mazza, L., et al. (2021). Effect of Discontinuing vs Continuing Angiotensin-Converting Enzyme Inhibitors and Angiotensin II Receptor Blockers on Days Alive and Out of the Hospital in Patients Admitted With COVID-19: A Randomized Clinical Trial. *JAMA* 325, 254–264. doi:10.1001/jama.2020.25864.

Mancia, G., Rea, F., Ludergnani, M., Apolone, G., and Corrao, G. (2020). Renin–Angiotensin–Aldosterone System Blockers and the Risk of Covid-19. *N Engl J Med* 382, 2431–2440. doi:10.1056/NEJMoa2006923.

Mariana, C. P., Ramona, P. A., Ioana, B. C., Diana, M., Claudia, R. C., Stefan, V. D., et al. (2016). Urinary angiotensin converting enzyme 2 is strongly related to urinary nephrin in type 2 diabetes patients. *Int Urol Nephrol* 48, 1491–1497. doi:10.1007/s11255-016-1334-8.

Matsuzawa, Y., Ogawa, H., Kimura, K., Konishi, M., Kirigaya, J., Fukui, K., et al. (2020). Renin–angiotensin system inhibitors and the severity of coronavirus disease 2019 in Kanagawa, Japan: a retrospective cohort study. *Hypertens Res* 43, 1257–1266. doi:10.1038/s41440-020-00535-8.

Mehta, N., Kalra, A., Nowacki, A. S., Anjewierden, S., Han, Z., Bhat, P., et al. (2020). Association of Use of Angiotensin-Converting Enzyme Inhibitors and Angiotensin II Receptor Blockers With Testing Positive for Coronavirus Disease 2019 (COVID-19). *JAMA Cardiol* 5, 1020. doi:10.1001/jamacardio.2020.1855.

Meng, J., Xiao, G., Zhang, J., He, X., Ou, M., Bi, J., et al. (2020). Renin-angiotensin system inhibitors improve the clinical outcomes of COVID-19 patients with hypertension. *Emerging Microbes & Infections* 9, 757–760. doi:10.1080/22221751.2020.1746200.

Mikami, T., Miyashita, H., Yamada, T., Harrington, M., Steinberg, D., Dunn, A., et al. (2020). Risk Factors for Mortality in Patients with COVID-19 in New York City. *J GEN INTERN MED*. doi:10.1007/s11606-020-05983-z.

Mizuiri, S., Aoki, T., Hemmi, H., Arita, M., Sakai, K., and Aikawa, A. (2011). Urinary angiotensin-converting enzyme 2 in patients with CKD: Urinary ACE2 in CKD. *Nephrology* 16, 567–572. doi:10.1111/j.1440-1797.2011.01467.x.

Nachtigall, I., Lenga, P., Jóźwiak, K., Thürmann, P., Meier-Hellmann, A., Kuhlen, R., et al. (2020). Clinical course and factors associated with outcomes among 1904 patients hospitalized with COVID-19 in Germany: an observational study. *Clin Microbiol Infect*. doi:10.1016/j.cmi.2020.08.011.

Narula, S., Yusuf, S., Chong, M., Ramasundarahettige, C., Rangarajan, S., Bangdiwala, S. I., et al. (2020). Plasma ACE2 and risk of death or cardiometabolic diseases: a case-cohort analysis. *The Lancet* 396, 968–976. doi:10.1016/S0140-6736(20)31964-4.

Negreira-Caamaño, M., Piqueras-Flores, J., Martínez-DelRio, J., Nieto-Sandoval-Martin-DeLaSierra, P., Aguila-Gordo, D., Mateo-Gomez, C., et al. (2020). Impact of Treatment with Renin-Angiotensin System Inhibitors on Clinical Outcomes in Hypertensive Patients Hospitalized with COVID-19. *High Blood Press Cardiovasc Prev*. doi:10.1007/s40292-020-00409-7.

Ocaranza, M. P., Godoy, I., Jalil, J. E., Varas, M., Collantes, P., Pinto, M., et al. (2006). Enalapril Attenuates Downregulation of Angiotensin-Converting Enzyme 2 in the Late Phase of Ventricular Dysfunction in Myocardial Infarcted Rat. *Hypertension* 48, 572–578. doi:10.1161/01.HYP.0000237862.94083.45.

Ortiz-Pérez, J. T., Riera, M., Bosch, X., De Caralt, T. M., Perea, R. J., Pascual, J., et al. (2013). Role of Circulating Angiotensin Converting Enzyme 2 in Left Ventricular Remodeling following Myocardial Infarction: A Prospective Controlled Study. *PLoS ONE* 8, e61695. doi:10.1371/journal.pone.0061695.

Palazzuoli, A., Mancone, M., De Ferrari, G. M., Forleo, G., Secco, G. G., Ruocco, G. M., et al. (2020). Antecedent Administration of Angiotensin‐Converting Enzyme Inhibitors or Angiotensin II Receptor Antagonists and Survival After Hospitalization for COVID‐19 Syndrome. *JAHA* 9. doi:10.1161/JAHA.120.017364.

Pan, W., Zhang, J., Wang, M., Ye, J., Xu, Y., Shen, B., et al. (2020). Clinical Features of COVID-19 in Patients With Essential Hypertension and the Impacts of Renin-angiotensin-aldosterone System Inhibitors on the Prognosis of COVID-19 Patients. *Hypertension* 76, 732–741. doi:10.1161/HYPERTENSIONAHA.120.15289.

Parra-Bracamonte, G. M., Lopez-Villalobos, N., and Parra-Bracamonte, F. E. (2020). Clinical characteristics and risk factors for mortality of patients with COVID-19 in a large data set from Mexico. *Annals of Epidemiology*, S1047279720302866. doi:10.1016/j.annepidem.2020.08.005.

Perez-Guzman, P. N., Daunt, A., Mukherjee, S., Crook, P., Forlano, R., Kont, M. D., et al. (2020). Clinical characteristics and predictors of outcomes of hospitalized patients with COVID-19 in a multi-ethnic London NHS Trust: a retrospective cohort study. *Clinical Infectious Diseases*, ciaa1091. doi:10.1093/cid/ciaa1091.

Petrilli, C. M., Jones, S. A., Yang, J., Rajagopalan, H., O’Donnell, L., Chernyak, Y., et al. (2020). Factors associated with hospital admission and critical illness among 5279 people with coronavirus disease 2019 in New York City: prospective cohort study. *BMJ*, m1966. doi:10.1136/bmj.m1966.

Raisi-Estabragh, Z., McCracken, C., Ardissino, M., Bethell, M. S., Cooper, J., Cooper, C., et al. (2020). Renin-Angiotensin-Aldosterone System Blockers Are Not Associated With Coronavirus Disease 2019 (COVID-19) Hospitalization: Study of 1,439 UK Biobank Cases. *Front. Cardiovasc. Med.* 7, 138. doi:10.3389/fcvm.2020.00138.

Ramchand, J., Patel, S. K., Kearney, L. G., Matalanis, G., Farouque, O., Srivastava, P. M., et al. (2020). Plasma ACE2 Activity Predicts Mortality in Aortic Stenosis and Is Associated With Severe Myocardial Fibrosis. *JACC: Cardiovascular Imaging* 13, 655–664. doi:10.1016/j.jcmg.2019.09.005.

Ramchand, J., Patel, S. K., Srivastava, P. M., Farouque, O., and Burrell, L. M. (2018). Elevated plasma angiotensin converting enzyme 2 activity is an independent predictor of major adverse cardiac events in patients with obstructive coronary artery disease. *PLoS ONE* 13, e0198144. doi:10.1371/journal.pone.0198144.

Reich, H. N., Oudit, G. Y., Penninger, J. M., Scholey, J. W., and Herzenberg, A. M. (2008). Decreased glomerular and tubular expression of ACE2 in patients with type 2 diabetes and kidney disease. *Kidney International* 74, 1610–1616. doi:10.1038/ki.2008.497.

Reilev, M., Kristensen, K. B., Pottegård, A., Lund, L. C., Hallas, J., Ernst, M. T., et al. (2020). Characteristics and predictors of hospitalization and death in the first 11 122 cases with a positive RT-PCR test for SARS-CoV-2 in Denmark: a nationwide cohort. *Int J Epidemiol*. doi:10.1093/ije/dyaa140.

Reynolds, H. R., Adhikari, S., Pulgarin, C., Troxel, A. B., Iturrate, E., Johnson, S. B., et al. (2020). Renin–Angiotensin–Aldosterone System Inhibitors and Risk of Covid-19. *N Engl J Med* 382, 2441–2448. doi:10.1056/NEJMoa2008975.

Richardson, S., Hirsch, J. S., Narasimhan, M., Crawford, J. M., McGinn, T., Davidson, K. W., et al. (2020). Presenting Characteristics, Comorbidities, and Outcomes Among 5700 Patients Hospitalized With COVID-19 in the New York City Area. *JAMA* 323, 2052. doi:10.1001/jama.2020.6775.

Sama, I. E., Ravera, A., Santema, B. T., van Goor, H., ter Maaten, J. M., Cleland, J. G. F., et al. (2020). Circulating plasma concentrations of angiotensin-converting enzyme 2 in men and women with heart failure and effects of renin–angiotensin–aldosterone inhibitors. *European Heart Journal* 41, 1810–1817. doi:10.1093/eurheartj/ehaa373.

Sands, K. E., Wenzel, R. P., McLean, L. E., Korwek, K. M., Roach, J. D., Miller, K. M., et al. (2020). Patient characteristics and admitting vital signs associated with coronavirus disease 2019 (COVID-19)-related mortality among patients admitted with noncritical illness. *Infect Control Hosp Epidemiol*, 1–7. doi:10.1017/ice.2020.461.

Sardu, C., Maggi, P., Messina, V., Iuliano, P., Sardu, A., Iovinella, V., et al. (2020). Could Anti‐Hypertensive Drug Therapy Affect the Clinical Prognosis of Hypertensive Patients With COVID‐19 Infection? Data From Centers of Southern Italy. *JAHA* 9. doi:10.1161/JAHA.120.016948.

Savarese, G., Benson, L., Sundström, J., and Lund, L. H. (2020). Association between Renin‐Angiotensin‐Aldosterone system inhibitor use and COVID‐19 Hospitalization and death: A 1,4 million patient Nation‐Wide registry analysis. *Eur J Heart Fail*, ejhf.2060. doi:10.1002/ejhf.2060.

Selçuk, M., Çınar, T., Keskin, M., Çiçek, V., Kılıç, Ş., Kenan, B., et al. (2020). Is the use of ACE inb/ARBs associated with higher in-hospital mortality in Covid-19 pneumonia patients? *Clinical and Experimental Hypertension* 42, 738–742. doi:10.1080/10641963.2020.1783549.

Seo, J., and Son, M. (2020). Update on association between exposure to renin-angiotensin-aldosterone system inhibitors and coronavirus disease 2019 in South Korea. *The Korean journal of internal medicine*. doi:10.3904/kjim.2020.380.

Shah, P., Owens, J., Franklin, J., Jani, Y., Kumar, A., and Doshi, R. (2020). Baseline use of angiotensin-converting enzyme inhibitor/AT1 blocker and outcomes in hospitalized coronavirus disease 2019 African-American patients. *Journal of Hypertension* Publish Ahead of Print. doi:10.1097/HJH.0000000000002584.

Soler, M. J., Ye, M., Wysocki, J., William, J., Lloveras, J., and Batlle, D. (2009). Localization of ACE2 in the renal vasculature: amplification by angiotensin II type 1 receptor blockade using telmisartan. *American Journal of Physiology-Renal Physiology* 296, F398–F405. doi:10.1152/ajprenal.90488.2008.

Son, M., Seo, J., and Yang, S. (2020). Association Between Renin-Angiotensin-Aldosterone System Inhibitors and COVID-19 Infection in South Korea. *Hypertension* 76, 742–749. doi:10.1161/HYPERTENSIONAHA.120.15464.

Soro-Paavonen, A., Gordin, D., Forsblom, C., Rosengard-Barlund, M., Waden, J., Thorn, L., et al. (2012). Circulating ACE2 activity is increased in patients with type 1 diabetes and vascular complications: *Journal of Hypertension* 30, 375–383. doi:10.1097/HJH.0b013e32834f04b6.

Stegbauer, J., Kraus, M., Nordmeyer, S., Kirchner, M., Ziehm, M., Dommisch, H.,et al. (2020). Proteomic analysis reveals upregulation of ACE2 (Angiotensin-Converting Enzyme 2), the putative SARS-CoV-2 receptor in pressure–but not volume-overloaded human hearts. Hypertension 76, e41–e43. doi: 10.1161/HYPERTENSIONAHA.120.16261.

Sukumaran, V., Veeraveedu, P. T., Gurusamy, N., Yamaguchi, K., Lakshmanan, A. P., Ma, M., et al. (2011). Cardioprotective Effects of Telmisartan against Heart Failure in Rats Induced By Experimental Autoimmune Myocarditis through the Modulation of Angiotensin-Converting Enzyme-2/Angiotensin 1-7/ *Mas* Receptor Axis. *Int. J. Biol. Sci.* 7, 1077–1092. doi:10.7150/ijbs.7.1077.

Sukumaran, V., Veeraveedu, P. T., Lakshmanan, A. P., Gurusamy, N., Yamaguchi, K., Ma, M., et al. (2012). Olmesartan medoxomil treatment potently improves cardiac myosin-induced dilated cardiomyopathy via the modulation of ACE-2 and ANG 1–7 mas receptor. *Free Radical Research* 46, 850–860. doi:10.3109/10715762.2012.684878.

Takeda, Y., Zhu, A., Yoneda, T., Usukura, M., Takata, H., and Yamagishi, M. (2007). Effects of Aldosterone and Angiotensin II Receptor Blockade on Cardiac Angiotensinogen and Angiotensin-Converting Enzyme 2 Expression in Dahl Salt-Sensitive Hypertensive Rats. *American Journal of Hypertension* 20, 1119–1124. doi:10.1016/j.amjhyper.2007.05.008.

Tedeschi, S., Giannella, M., Bartoletti, M., Trapani, F., Tadolini, M., Borghi, C., et al. (2020). Clinical Impact of Renin-angiotensin System Inhibitors on In-hospital Mortality of Patients With Hypertension Hospitalized for Coronavirus Disease 2019. *Clinical Infectious Diseases* 71, 899–901. doi:10.1093/cid/ciaa492.

Uri, K., Fagyas, M., Kertesz, A., Borbely, A., Jenei, C., Bene, O., et al. (2016). Circulating ACE2 activity correlates with cardiovascular disease development. *Journal of the Renin-Angiotensin-Aldosterone System* 17, 17/4/1470320316668435. doi:10.1177/1470320316668435.

van Gerwen, M., Alsen, M., Little, C., Barlow, J., Genden, E., Naymagon, L., et al. (2020). Risk factors and outcomes of COVID-19 in New York City; a retrospective cohort study. *Journal of Medical Virology* n/a. doi:10.1002/jmv.26337.

Velkoska, E., Dean, R. G., Burchill, L., Levidiotis, V., and Burrell, L. M. (2010). Reduction in renal ACE2 expression in subtotal nephrectomy in rats is ameliorated with ACE inhibition. *Clinical Science* 118, 269–279. doi:10.1042/CS20090318.

Vuille-dit-Bille, R. N., Camargo, S. M., Emmenegger, L., Sasse, T., Kummer, E., Jando, J., et al. (2015). Human intestine luminal ACE2 and amino acid transporter expression increased by ACE-inhibitors. *Amino Acids* 47, 693–705. doi:10.1007/s00726-014-1889-6.

Walters, T. E., Kalman, J. M., Patel, S. K., Mearns, M., Velkoska, E., and Burrell, L. M. (2016). Angiotensin converting enzyme 2 activity and human atrial fibrillation: increased plasma angiotensin converting enzyme 2 activity is associated with atrial fibrillation and more advanced left atrial structural remodelling. *Europace*, euw246. doi:10.1093/europace/euw246.

Wang, G., Zhang, Q., Yuan, W., Wu, J., and Li, C. (2016). Enalapril protects against myocardial ischemia/reperfusion injury in a swine model of cardiac arrest and resuscitation. *International Journal of Molecular Medicine* 38, 1463–1473. doi:10.3892/ijmm.2016.2737.

Wang, X., Fang, J., Zhu, Y., Chen, L., Ding, F., Zhou, R., et al. (2020). Clinical characteristics of non-critically ill patients with novel coronavirus infection (COVID-19) in a Fangcang Hospital. *Clinical Microbiology and Infection* 26, 1063–1068. doi:10.1016/j.cmi.2020.03.032.

Williamson, E. J., Walker, A. J., Bhaskaran, K., Bacon, S., Bates, C., Morton, C. E., et al. (2020). Factors associated with COVID-19-related death using OpenSAFELY. *Nature* 584, 430–436. doi:10.1038/s41586-020-2521-4.

Wösten-van Asperen, R. M., Lutter, R., Specht, P. A., Moll, G. N., van Woensel, J. B., van der Loos, C. M., et al. (2011). Acute respiratory distress syndrome leads to reduced ratio of ACE/ACE2 activities and is prevented by angiotensin-(1-7) or an angiotensin II receptor antagonist: ARDS leads to reduced ratio of ACE/ACE2 activities. *J. Pathol.* 225, 618–627. doi:10.1002/path.2987.

Wu C., Ye Dien, Mullick A. E., Li Z., Danser A.H. J., Daugherty A. et al. (2020). Effects of Renin-Angiotensin Inhibition on ACE2 (Angiotensin-Converting Enzyme 2) and TMPRSS2 (Transmembrane Protease Serine 2) Expression. *Hypertension* 76, e29–e30. doi:10.1161/HYPERTENSIONAHA.120.15782.

Wysocki, J., Lores, E., Ye, M., Soler, M. J., and Batlle, D. (2020). Kidney and Lung ACE2 Expression after an ACE Inhibitor or an Ang II Receptor Blocker: Implications for COVID-19. *JASN* 31, 1941–1943. doi:10.1681/ASN.2020050667.

Xu, J., Huang, C., Fan, G., Liu, Z., Shang, L., Zhou, F., et al. (2020). Use of angiotensin-converting enzyme inhibitors and angiotensin II receptor blockers in context of COVID-19 outbreak: a retrospective analysis. *Front Med*, 1–12. doi:10.1007/s11684-020-0800-y.

Yahyavi, A., Hemmati, N., Derakhshan, P., Banivaheb, B., Karimi Behnagh, A., Tofighi, R., et al. (2020). Angiotensin enzyme inhibitors and angiotensin receptor blockers as protective factors in COVID-19 mortality: a retrospective cohort study. *Intern Emerg Med*. doi:10.1007/s11739-020-02523-9.

Yang, G., Tan, Z., Zhou, L., Yang, M., Peng, L., Liu, J., et al. (2020). Effects of Angiotensin II Receptor Blockers and ACE (Angiotensin-Converting Enzyme) Inhibitors on Virus Infection, Inflammatory Status, and Clinical Outcomes in Patients With COVID-19 and Hypertension: A Single-Center Retrospective Study. *Hypertension* 76, 51–58. doi:10.1161/HYPERTENSIONAHA.120.15143.

Yang, Z., Yu, X., Cheng, L., Miao, L., Li, H., Han, L., et al. (2013). Effects of enalapril on the expression of cardiac angiotensin-converting enzyme and angiotensin-converting enzyme 2 in spontaneously hypertensive rats. *Archives of Cardiovascular Diseases* 106, 196–201. doi:10.1016/j.acvd.2013.01.004.

Zhang, Y., Li, B., Wang, B., Zhang, J., Wu, J., and Morgan, T. (2014). Alteration of cardiac ACE2/Mas expression and cardiac remodelling in rats with aortic constriction. Chinese J. Physiol. 57, 335–342. doi: 10.4077/CJP.2014.BAD268.

Yu, C., Lei, Q., Li, W., Wang, X., Liu, W., Fan, X., et al. (2020). Clinical Characteristics, Associated Factors, and Predicting COVID-19 Mortality Risk: A Retrospective Study in Wuhan, China. *American Journal of Preventive Medicine* 59, 168–175. doi:10.1016/j.amepre.2020.05.002.

Zhang, P., Zhu, L., Cai, J., Lei, F., Qin, J.-J., Xie, J., et al. (2020). Association of Inpatient Use of Angiotensin-Converting Enzyme Inhibitors and Angiotensin II Receptor Blockers With Mortality Among Patients With Hypertension Hospitalized With COVID-19. *Circ Res* 126, 1671–1681. doi:10.1161/CIRCRESAHA.120.317134.

Zhou, F., Liu, Y.-M., Xie, J., Li, H., Lei, F., Yang, H., et al. (2020). Comparative Impacts of ACE (Angiotensin-Converting Enzyme) Inhibitors Versus Angiotensin II Receptor Blockers on the Risk of COVID-19 Mortality. *Hypertension* 76. doi:10.1161/HYPERTENSIONAHA.120.15622.
